# Supplementary material for: TCF7L2 rs7903146 polymorphism association with diabetes and obesity in an elderly cohort from Brazil
Source: PeerJ. 2021 May 5;9:e11349. doi: 10.7717/peerj.11349 (PMC8106398; doi:10.7717/peerj.11349)
Supplement: Supplemental Information 1 — Data are presented as N° (%); Total means all together, including volunteers both with and without type 2 diabetes mellitus. BMI classification criteria: Normal-weight (18.5–24.9 kg/m2), overweight (25.0–29.9 kg/m2), obesity (≥ 30.0 kg/m2). Non-T2D, without type 2 diabetes mellitus; T2D, with type 2 diabetes mellitus. [file peerj-09-11349-s001.docx]

**Supplemental Table 1**
Genotypic and allelic distributions by body mass index status and by glycemic status.

| Glycemic Status | Genotype/Allele | Normal-weight (N=280)  N (%) | Overweight (N=424)  N (%) | Obesity (N=319)  N (%)  N (%) |  |
| --- | --- | --- | --- | --- | --- |
| Non-T2D | CC | 106 (46) | 136 (44) | 120 (53) |  |
|  | CT | 105 (46) | 148 (48) | 90 (40) |  |
|  | TT | 18 (08) | 23 (08) | 17 (07) |  |
|  | C | 317 (69) | 420 (68) | 330 (73) |  |
|  | T | 141 (31) | 194 (32) | 124 (27) |  |
|  |  |  |  |  |  |
| T2D | CC | 21 (41) | 47 (40) | 46 (50) |  |
|  | CT | 19 (37) | 54 (46) | 38 (41) |  |
|  | TT | 11 (22) | 16 (14) | 08 (09) |  |
|  | C | 61 (60) | 148 (63) | 130 (71) |  |
|  | T | 41 (40) | 86 (27) | 54 (29) |  |
|  |  |  |  |  |  |
| Total | CC | 127 (46) | 183 (43) | 166 (52) |  |
|  | CT | 124 (44) | 202 (48) | 128 (40) |  |
|  | TT | 29 (10) | 39 (09) | 25 (08) |  |
|  | C | 378 (68) | 568 (67) | 460 (72) |  |
|  | T | 182 (32) | 280 (33) | 178 (30) |  |
| Data are presented as Nº (%); Total means all together, including volunteers both with and without type 2 diabetes mellitus.  BMI classification criteria: Normal-weight (18.5–24.9 kg/m^2^), overweight (25.0–29.9 kg/m^2^), obesity (≥ 30.0 kg/m^2^). Non-T2D, without type 2 diabetes mellitus; T2D, with type 2 diabetes mellitus. | | | | | |
