# Supplementary material for: TCF7L2 rs7903146 polymorphism association with diabetes and obesity in an elderly cohort from Brazil
Source: PeerJ. 2021 May 5;9:e11349. doi: 10.7717/peerj.11349 (PMC8106398; doi:10.7717/peerj.11349)
Supplement: Supplemental Information 2 — *Mann-whitney test **Kruskall-Wallis test Ancestries abbreviation: EUR. European; AFR. African; NAM. Native American; EAS. East Asia [file peerj-09-11349-s002.docx]

| **Supplemental Table 2.** Ancestries frequency included in the elderly cohort. | | | | | | |
| --- | --- | --- | --- | --- | --- | --- |
| Classification criteria | Group | N | Avarage frequency of ancestries (%) | | | |
|  |  |  | EUR | AFR | NAM | EAS |
| T2DM presence | T2DM | 260 | 0*.*69 | 0*.*21 | 0*.*04 | 0*.*07 |
|  | non-T2DM | 763 | 0*.*74 | 0*.*17 | 0*.*03 | 0*.*07 |
| *P*-value* |  |  | **0*.*020** | 0*.*057 | 0*.*242 | 0*.*996 |
| Obesity Status | non-obese | 704 | 0*.*73 | 0*.*17 | 0*.*04 | 0*.*07 |
|  | obese | 319 | 0*.*72 | 0*.*19 | 0*.*02 | 0*.*07 |
| *P*-value* |  |  | 0*.*639 | 0*.*122 | 0*.*272 | 0*.*780 |
| BMI status | Normal weight | 280 | 0*.*72 | 0*.*16 | 0*.*06 | 0*.*07 |
|  | overweight | 424 | 0*.*73 | 0*.*18 | 0*.*02 | 0*.*07 |
|  | obesity | 319 | 0*.*72 | 0*.*19 | 0*.*02 | 0*.*07 |
| *P*-value** |  |  | 0*.*890 | 0*.*077 | 0*.*361 | 0*.*946 |
| Genotype | CC | 476 | 0*.*70 | 0*.*18 | 0*.*05 | 0*.*07 |
|  | CT | 454 | 0*.*75 | 0*.*18 | 0*.*01 | 0*.*07 |
|  | TT | 93 | 0*.*78 | 0*.*17 | 0*.*00 | 0*.*06 |
| *P*-value* |  |  | **0*.*007** | 0*.*667 | 0*.*060 | 0*.*120 |
| Genetic Model | CC+CT | 930 | 0*.*72 | 0*.*18 | 0*.*03 | 0*.*07 |
|  | TT | 93 | 0*.*78 | 0*.*17 | 0*.*00 | 0*.*06 |
| *P*-value* |  |  | 0*.*060 | 0*.*454 | 0*.*958 | 0*.*066 |
| Total Population |  | 1023 | 0*.*73 | 0*.*18 | 0*.*03 | 0*.*07 |
| *Mann-whitney test  **Kruskall-Wallis test Ancestries abbreviation: EUR. European; AFR. African; NAM. Native American; EAS. East Asia | | | | | | |
