# Supplementary material for: TCF7L2 rs7903146 polymorphism association with diabetes and obesity in an elderly cohort from Brazil
Source: PeerJ. 2021 May 5;9:e11349. doi: 10.7717/peerj.11349 (PMC8106398; doi:10.7717/peerj.11349)
Supplement: Supplemental Information 3 — Genotypes are stratified under the recessive genetic model for T allele (CC+CT Vs TT). Data are presented as mean ± SD for the most variables; P-value with Mann-Whitney test for quantitative variables and Chi-square test for qualitative data. P, P-value; T2DM, Type 2 diabetes mellitus; M/F, Male/Female; BMI, Body mass index; LDL, low density lipoprotein; HDL, high density lipoprotein. [file peerj-09-11349-s003.docx]

**Supplemental Table 3**Anthropometric and biochemical characteristics stratified according to genotypes.

| Variable | Unit | CC+CT (N=930) | TT (N=93) | P |
| --- | --- | --- | --- | --- |
|  |  |  |  |  |
| Age | years old | 72.49 ± 0.30 | 72.62 ± 0.88 | 0.7403 |
| BMI | kg/m² | 28.12 ± 0.17 | 27.74 ± 0.49 | 0.4739 |
| Waist circumference | cm | 94.26 ± 0.40 | 94.13 ± 1.11 | 0.8894 |
| Hip circumference | cm | 103.20 ± 0.36 | 102.60 ± 1.04 | 0.6124 |
| Hip-waist ratio | cm/cm | 0.91 ± 0.01 | 0.92 ± 0.01 | 0.4722 |
| Systolic pressure | mmHg | 140.80 ± 0.73 | 139.00 ± 2.46 | 0.4819 |
| Diastolic pressure | mmHg | 79.84 ± 0.39 | 78.59 ± 1.24 | 0.3123 |
| Plasma glucose | mg/dL | 98.22 ± 1.24 | 101.40 ± 3.26 | 0.0556 |
| Glycated hemoglobin | % | 6.10 ± 0.04 | 6.20 ± 0.13 | 0.5500 |
| Total cholesterol | mg/dL | 205.10 ± 1.35 | 204.30 ± 4.42 | 0.7883 |
| Fasting triglyceride | mg/dL | 134.90 ± 2.93 | 135.00 ± 6.62 | 0.4566 |
| LDL cholesterol | mg/dL | 128.80 ± 1.15 | 128.90 ± 3.71 | 0.9696 |
| HDL cholesterol | mg/dL | 49.77 ± 0.46 | 48.42 ± 1.41 | 0.4741 |
| Gender M/F | N/N | 330/600 | 35/58 | 0.6798 |
| BMI ≥30kg/m² | N (%) | 294 (34) | 25 (27) | 0.3477 |
| Hypertension | N (%) | 633 (68) | 66 (71) | 0.5661 |
| T2DM | N (%) | 225 (24) | 35 (38) | **0.0045** |

Genotypes are stratified under the recessive genetic model for T allele (CC+CT Vs TT).

Data are presented as mean ± SD for the most variables; P-value with Mann-Whitney test for quantitative variables and Chi-square test for qualitative data.
P, P-value; T2DM, Type 2 diabetes mellitus; M/F, Male/Female; BMI, Body mass index; LDL, low density lipoprotein; HDL, high density lipoprotein.
