# Supplementary material for: TCF7L2 rs7903146 polymorphism association with diabetes and obesity in an elderly cohort from Brazil
Source: PeerJ. 2021 May 5;9:e11349. doi: 10.7717/peerj.11349 (PMC8106398; doi:10.7717/peerj.11349)
Supplement: Supplemental Information 4 — Volunteers with type 2 diabetes and/or fasting glucose above 125 mg/dL were considered as the case group (N=278) and the others were included in the control group (N=745). *P-values are from logistic regression models adjusted for age and gender. [file peerj-09-11349-s004.docx]

| **Suplemental Table 4**. Association between hyperglycemic status and the rs7903146 T allele. | | | | |  |
| --- | --- | --- | --- | --- | --- |
| Genetic Model | | *P-*value* | Odds Ratio | 95% Confidence Interval | |
| Dominant (CC *Vs* TT+CT) | 0.004 | | 1.77 | 2.61 – 1.20 | |
| Recessive (CC+CT *Vs* TT) | 0.067 | | 1.78 | 3.28 – 0.96 | |
| Log-additive (0, 1, 2) | 0.002 | | 1.56 | 2.09 – 1.17 | |
| Volunteers with type 2 diabetes and/or fasting glucose above 125 mg/dL were considered as the case group (N=278) and the others were included in the control group (N=745).  **P*-values are from logistic regression models adjusted for age and gender. | | | | |  |
