# Supplementary material for: TCF7L2 rs7903146 polymorphism association with diabetes and obesity in an elderly cohort from Brazil
Source: PeerJ. 2021 May 5;9:e11349. doi: 10.7717/peerj.11349 (PMC8106398; doi:10.7717/peerj.11349)
Supplement: Supplemental Information 5 — P-values are from logistic regression models adjusted for BMI, age and gender. Abbreviations: OR, odds ratio; CI, confidence interval. [file peerj-09-11349-s005.docx]

| **Supplemental Table 5**  Association of *TCF7L2* rs7903146 C allele with T2DM risk. | | | |
| --- | --- | --- | --- |
| Genetic Model | Clustering Model | *P*-value | OR (95% CI) |
| Dominant | TT *Vs* CC+CT | 0.003 | 0.51 (0.32 - 0.80) |
| Recessive | TT+CT *Vs* CC | 0.260 | 0.85 (0.64 - 1.13) |
| Additive | TT *Vs* CC | 0.004 | 0.50 (0.31 - 0.81) |
| Allelic | T *Vs* C | 0.031 | 0.79 (0.64 - 0.98) |
| *P*-values are from logistic regression models adjusted for BMI, age and gender. Abbreviations: OR, odds ratio; CI, confidence interval. | | | |
