# Supplementary material for: TCF7L2 rs7903146 polymorphism association with diabetes and obesity in an elderly cohort from Brazil
Source: PeerJ. 2021 May 5;9:e11349. doi: 10.7717/peerj.11349 (PMC8106398; doi:10.7717/peerj.11349)
Supplement: Supplemental Information 6 — P, P-value; 95% CI, 95% confidence interval; OR, odds ratio OR adjusted for sex, type 2 diabetes presence and age. BMI classification criteria: normal-weight (18.5–24.9 kg/m2), overweight (25.0–29.9 kg/m2), obesity (≥ 30.0 kg/m2). [file peerj-09-11349-s006.docx]

**Supplemental Table 6**

Association of the rs7903146 T allele with the Body Mass Index Status.

| Control group | Case group | Dominant model  (CC Vs TT+CT), | |  | Recessive Model  (CC+CT Vs TT), | |  | Additive Model  (CC Vs TT) | |  | T Allele  (C Vs T) | |
| --- | --- | --- | --- | --- | --- | --- | --- | --- | --- | --- | --- | --- |
|  |  | OR (95% CI) | P |  | OR (95% CI) | P |  | OR (95% CI) | P |  | OR (95% CI) | P |
| Normal weight | overweight | 1.09 (0.80 - 1.48 ) | 0.603 |  | 0.80 (0.48 - 1.35 ) | 0.408 |  | 0.87 (0.50 - 1.50 ) | 0.615 |  | 1.00 (0.80 - 1.26 ) | 0.980 |
| Normal weight | obesity | 0.71 (0.50 - 1.00 ) | 0.050 |  | 0.62 (0.34 - 1.14 ) | 0.124 |  | 0.58 (0.31 - 1.07 ) | 0.079 |  | **0.74 (0.57 - 0.97 )** | **0.028** |
| Normal weight | obesity + overweight | 0.93 (0.70 - 1.23 ) | 0.615 |  | 0.75 (0.47 - 1.21 ) | 0.247 |  | 0.76 (0.46 - 1.25 ) | 0.285 |  | 0.91 (0.73 - 1.12 ) | 0.376 |
| Normal weight + overweight | obesity | **0.71 (0.54 - 0.94 )** | **0.016** |  | 0.77 (0.47 - 1.26 ) | 0.301 |  | 0.67 (0.40 - 1.12 ) | 0.126 |  | **0.78 (0.63 - 0.97 )** | **0.022** |

P, P-value; 95% CI, 95% confidence interval; OR, odds ratio OR adjusted for sex, type 2 diabetes presence and age.
BMI classification criteria: normal-weight (18.5–24.9 kg/m^2^), overweight (25.0–29.9 kg/m^2^), obesity (≥ 30.0 kg/m^2^).
