# Supplementary material for: TCF7L2 rs7903146 polymorphism association with diabetes and obesity in an elderly cohort from Brazil
Source: PeerJ. 2021 May 5;9:e11349. doi: 10.7717/peerj.11349 (PMC8106398; doi:10.7717/peerj.11349)
Supplement: Supplemental Information 7 — Comparing the first versus second tertile, significant values were found for the T allele on additive genetic model (OR 5.13; 95% CI 1.40-18.93; P=0.009) and on recessive model (OR 5.13; 95% CI 1.43-18.37; P=0.010) using Fisher’s exact test [file peerj-09-11349-s007.docx]

**Supplemental Table 7**

Genotypic distributions by tertile intervals from ∆BMI values of volunteers without type 2 diabetes mellitus.

| rs7903146 Genotype | No (%) of participants per ∆BMI’s tertile interval | | |
| --- | --- | --- | --- |
|  | T1 (-6.8 − -0.6) | T2 ( -0.6 − 1.4) | T3 (1.4 – 8.4) |
| CC | 55 (34) | 50 (31) | 57 (35) |
| CT | 56 (36) | 51 (32) | 50 (32) |
| TT | 03 (13) | 14 (58)* | 07 (29) |
| Comparing the first versus second tertile, significant values were found for the T allele on additive genetic model (OR 5.13; 95% CI 1.40-18.93; *P=*0.009) and on recessive model (OR 5.13; 95% CI 1.43-18.37; *P=*0.010) using Fisher's exact test. | | | |
