# Supplementary material for: TCF7L2 rs7903146 polymorphism association with diabetes and obesity in an elderly cohort from Brazil
Source: PeerJ. 2021 May 5;9:e11349. doi: 10.7717/peerj.11349 (PMC8106398; doi:10.7717/peerj.11349)
Supplement: Supplemental Information 8 — No significant differences were found between genotypes. [file peerj-09-11349-s008.docx]

**Supplemental Table 8**

Genotypic distributions by tertile intervals from ∆BMI values of volunteers with type 2 diabetes mellitus.

| rs7903146 Genotype | No (%) of participants per ∆BMI’s tertile interval | | |
| --- | --- | --- | --- |
|  | T1 (-6.6 − -1.8) | T2 (-1.8 – 0.7) | T3 (0.8 – 7.6) |
| CC | 22 (34) | 24 (37) | 19 (29) |
| CT | 20 (36) | 14 (25) | 22 (39) |
| TT | 06 (26) | 10 (43) | 07 (30) |
| No significant differences were found between genotypes. | | | |
