# Supplementary material for: TCF7L2 rs7903146 polymorphism association with diabetes and obesity in an elderly cohort from Brazil
Source: PeerJ. 2021 May 5;9:e11349. doi: 10.7717/peerj.11349 (PMC8106398; doi:10.7717/peerj.11349)
Supplement: Supplemental Information 9 — SABE survey is fully available in the original language (Portuguese)—http://hygeia3.fsp.usp.br/sabe/quetionario.php. A translation of the sections and questions used can be shared. [file peerj-09-11349-s009.pdf]

QUESTIONÁRIO C10

DATA DESTA ENTREVISTA: Dia I___I___I Mês I___I___I 201__

Nome da entrevistadora: ____________________

Hora de início: |___|___|:|___|___| Hora de término: |___|___|:|___|___|

ESTUDO SABE - SAUDE, BEM ESTAR E ENVELHECIMENTO

NUMERO DO QUESTIONÁRIO | | | | | | | | | | |

Nome completo do entrevistado: ____________________

Sexo: Mulher 1 Homem 2

Telefone do(a) idoso(a): ____________________

Endereço: (rua, numero externo e interno, CEP)

1. Nome: ____________________ Parentesco com idoso: __________

   Telefones de contato: ____________________

2. Nome: ____________________ Parentesco com idoso: __________

   Telefones de contato: ____________________

3. Nome: ____________________ Parentesco com idoso: __________

   Telefones de contato: ____________________

4. Nome: ____________________ Parentesco com idoso: __________

   Telefones de contato: ____________________

ATENÇÃO: PREENCHER

ATENÇÃO: REVISAR AO FINAL DA ENTREVISTA CÓD. RESULTADOS

Resultado* | | |

* Códigos de Resultados:

01 Entrevista completa ( ) 1 dia ( ) + de um dia
02 Entrevista completa com informante auxiliar
03 Entrevista completa com informante substituto
04 Entrevista incompleta (anote em observações)
05 Entrevista adiada (anote em observações)
06 Ausente temporário
07 Nunca encontrou a pessoa
08 Recusou-se
09 Incapacitado e sem informante
10 Outros (anote em observações)

Nome do informante: ____________________ Parentesco com idoso: __________

Caso não seja familiar, há quanto tempo conhece o(a) idoso(a): ____________________

Entregue	Crítica	Revisto	P/digit	Checagem	Codificação	Digitação	REGISTRO EPIDATA
___ __/__/__	___ __/__/__	___ __/__/__	___ __/__/__	___ __/__/__	___ __/__/__	___ __/__/__	___

Observações: ____________________

SEÇÃO A - INFORMAÇÕES PESSOAIS

HORA DE INÍCIO: |__|__| : |__|__|

DECLARAÇÃO VOLUNTÁRIA

Antes de começar, gostaria de assegurar-lhe que esta entrevista é completamente voluntária e confidencial. Se houver alguma pergunta que o Sr (a) não deseje responder, simplesmente me avise e seguiremos para a próxima pergunta.

Pergunta	Resposta
A.1a<br>**Em que dia, mês e ano o(a) Sr(a) nasceu?**<br>**A. 01**	DIA E MÊS...|____|____|..........|____|____| DIA MÊS<br>ANO.. .|____|____|____|____|
A.1b<br>**Quantos anos completos o(a) Sr.(a) tem?**<br>**A. 02**	Nº DE ANOS..|____|____|____|
A.2<br>**O(a) Sr(a) nasceu no Brasil?**<br>**A. 03**	SIM .... **1** → **A. 06**<br>NÃO .... **2**<br>NS .... **8**<br>NR **9**
A.3<br>**Em que país o(a) Sr(a) nasceu?**<br>**A. 04**	País ____________________<br>____________________<br>____________________
A.4a<br>**No total, há quantos anos o(a) Sr(a). vive no Brasil?**<br>**A. 05**	IDADE..|____|____|____| NS .... 998<br>NR 999
A.4b<br>**Desde que o Sr.(a) nasceu até os 15 anos, viveu no campo por 5 anos ou mais?**<br>**A. 06**	SIM .... 1<br>NÃO .... 2<br>NS .... 8<br>NR 9
A.5a<br>**O(a) Sr.(a) sabe ler e escrever um recado?**<br>**A. 07**	SIM .... 1<br>NÃO........................ 2<br>NR 9
A.5b<br>**O(a) Sr.(a) foi à escola?**<br>**A. 08**	SIM .... 1<br>NÃO .... 2<br>NS .... 8<br>NR .... 9<br>(2, 8, 9) → **A.10**

SEÇÃO A - INFORMAÇÕES PESSOAIS

A.6

Qual a última série (e de que grau) da escola em que o Sr(a) obteve aprovação?

ANOTE A SÉRIE DO ÚLTIMO GRAU APROVADO

A.09

ÚLTIMA SÉRIE(ano escolar) CURSADA PELO (A) IDOSO(A):

____

Primeiro grau (ou primário + ginásio) .... 01 |____|

Segundo grau (antigo clássico e científico) .... 02 |____|

Técnico de nível médio (ex: técnico em contabilidade, laboratório) .... 04 |____|

Magistério - segundo grau (antigo normal) ...... 06 |____|

Graduação (nível superior) .... 07 |____|

Pós-graduação .... 08 |____|

Supletivo/Madureza....................................... 09 |____|

NS.. 988

NR.. 999

A.7

Atualmente o(a) Sr(a). vive sozinho(a) ou acompanhado(a)

A.10

SOZINHO 1

ACOMPANHADO 2

NR 9

A.7a

Há 5 anos, o Sr(a) morava: - exatamente com as MESMAS PESSOAS com quem mora agora OU SOZINHO?

UTILIZE A REDAÇÃO CORRESPONDENTE SEGUNDO O CASO

A.11

SIM .... 1 → **A. 15**

NÃO 2

NR 9

A.7b

Há 5 anos o(a) Sr.(a) vivia...

LEIA CADA OPÇÃO E CIRCULE TODAS AS AFIRMATIVAS

A.12

SÓ?	**10**
COM ESPOSO(A) OU COMPANHEIRO (A)?	**20**
COM UM OU MAIS FILHO(A)(S) CASADO(A)(S) ?	**30**
COM UM OU MAIS FILHO(A)(S) SOLTEIRO(A)(S) ?	**40**
COM OUTRO FAMILIAR?	**50**
COM OUTRO NÃO FAMILIAR REMUNERADO QUE O AJUDAVA?	**61**
COM OUTRO NÃO FAMILIAR NÃO REMUNERADO?	**62**
COM EMPREGADA DOMÉSTICA?	**70**
NS	**98**
NR	**99**

A.7c

Por que o(a) Sr(a) já não mora com as mesmas pessoas com quem morava há 5 anos?

ANOTE TODAS AS OPÇÕES MENCIONADAS ESPONTANEAMENTE

A. 13

RAZÕES RELACIONADAS AOS FILHOS DO(A) IDOSO(A):

ECONÔMICAS **01**

SEPARAÇÃO CONJUGAL **02**

UNIÃO CONJUGAL **03**

PARA CUIDAR DAS CRIANÇAS **04**

RAZÕES RELACIONADAS COM O(A) IDOSO(A):

ECONÔMICAS **05**

SEPARAÇÃO CONJUGAL **06**

UNIÃO CONJUGAL **13**

FALECIMENTO **07**

____
Especifique

SE SENTIA SÓ **08**

PRECISAVA DE AJUDA **09**

____
Especifique

OUTRO **11**

____
Especifique

ACOLHIMENTO DE PARENTE (neto, sobrinho, etc)) **12**

NS **98**

NR **99**

A.9

Se o(a) Sr(a) pudesse escolher, preferiria morar...

LEIA AS OPÇÕES E ANOTE TODAS AS AFIRMATIVAS

A. 15

SÓ?.......... **10**

COM ESPOSO(A) OU COMPANHEIRO (A)?.......... **20**

COM UM OU MAIS FILHO(A)(S) CASADO(A)(S) ?.......... **30**

COM UM OU MAIS FILHO(A)(S) SOLTEIRO(A)(S) ?.......... **31**

COM NETO.......... **40**

COM OUTRO FAMILIAR?.......... **50**

COM OUTRO NÃO FAMILIAR REMUNERADO QUE O AJUDAVA?.......... **61**

COM OUTRO NÃO FAMILIAR NÃO REMUNERADO? **62**

EMPREGADA DOMÉSTICA?.......... **70**

NS.......... **98**

NR.......... **99**

A.10a

Há 5 anos, o(a) Sr.(a) morava nesta mesma casa?

A. 16

SIM	1 → **A. 18**
NÃO	2
NR	9

A.10b

Qual a principal razão pela qual o(a) Sr.(a) se mudou?

ANOTE SOMENTE **UMA** RESPOSTA

A. 17

ESTAR PERTO DE OU COM O(A) FILHO(A)	01
ESTAR PERTO DE OU COM FAMILIARES OU AMIGOS	02
ESTAR PERTO DOS SERVIÇOS DE SAÚDE	03
PARA SE SENTIR MAIS SEGURO (MEDO DA VIOLÊNCIA)	04
FALECIMENTO DO(A) ESPOSO(A)/COMPANHEIRO(A)	05
POR SEPARAÇÃO CONJUGAL (do(a) entrevistado(a)	06
POR UNIÃO CONJUGAL (do(a) entrevistado(a)	07
DIMINUIR DESPESAS	08
PELAS CONDIÇÕES DA MORADIA (BARREIRAS ARQUITETÔNICAS)	10
QUESTÕES IMOBILIÁRIAS	11
OUTRO *ESPECIFIQUE*	09
NS	98
NR	99

A.11a

Qual é sua religião/credo?

A. 18

CATÓLICA	10
PROTESTANTE	21
EVANGÉLICA	22
JUDAICA	30
ESPÍRITA KARDECISTA	71
BUDISTA	72
OUTRO ______ *Especifique*	50
NENHUMA	60 → **A. 26**
NS	98
NR	99

A.11b

Qual a importância da religião em sua vida?

A. 19

IMPORTANTE	1
REGULAR (+ ou -)	2
NADA IMPORTANTE	3
NS	8
NR	9

A.11c

Com que freqüência o(a) Sr.(a) vai a igreja ou ao serviço religioso?

A. 20

NUNCA	1
VÁRIAS VEZES AO ANO	2
UMA OU DUAS VEZES POR MÊS	3
QUASE TODA SEMANA	4
MAIS DE UMA VEZ POR SEMANA	5
NS	8
NR	9

A.11d

Quanto, na sua opinião, a sua religião lhe dá FORÇAS PARA ENFRENTAR as dificuldades?

A. 21

COMPLETAMENTE	1
MUITO	2
NÃO MUITO	3
NADA	4
NS	8
NR	9

A.11e

Quanto, na sua opinião, sua religião o(a) AJUDA A ENTENDER as dificuldades na vida?

A. 22

COMPLETAMENTE	1
MUITO	2
NÃO MUITO	3
NADA	4
NS	8
NR	9

A.11f

Na sua opinião, sua religião DÁ SENTIDO à sua vida?

A. 23

TOTALMENTE	1
MUITO	2
ÀS VEZES	3
NUNCA	4
NS	8
NR	9

A.11g

Quanto o(a) Sr.(a) SE CONSIDERA religioso(a)?

A. 24

MUITO	1
UM POUCO	2
NÃO MUITO	3
NADA	4
NS	8
NR	9

A.11h

Com que freqüência o(a) Sr(a) reza (ora) ou realiza suas práticas religiosas?

A. 25

VÁRIAS VEZES AO DIA	1
UMA VEZ AO DIA	2
VÁRIAS VEZES POR SEMANA	3
SOMENTE EM OCASIÕES ESPECIAIS	4
QUASE NUNCA OU NUNCA	5
NS	8
NR	9

A.12

Qual destas opções o descreve melhor?

A. 26

BRANCO (de origem européia)	1
PARDO / MULATO / MORENO (combinação de branco e preto) .	3
PRETO	4
INDÍGENA	5
AMARELO	6
OUTRA ______________________ *Especifique*	7
NS	8
NR	9

A.12a

O(A) Sr(a) tem algum parente: (mãe ou pai), (avó/avô) ou (bisavó/bisavô) nascido no Japão?

A. 27

SIM

- 1ª geração (mãe / pai) 1
- 2ª geração (avó / avô)........................... 2
- 3ª geração (bisavó / bisavô) 3

NÃO 4

NÃO SABE 8

NÃO RESPONDE 9

HISTÓRIA DE UNIÕES

A.13a

Com relação ao seu estado marital, alguma vez o(a) Sr.(a) foi casado(a) ou teve uma união livre?

A. 28

SIM 1

NÃO .... 2 → A. 32

NR 9

A.13b

No total, quantas vezes, o(a) Sr.(a) esteve casado(a) ou em união?

A. 29

Nº DE VEZES ....|___|___|

A.13c

Qual o seu estado marital hoje?

A. 30

DIVORCIADO..1

SEPARADO.. 2

VIÚVO.. . 3

CASADO.. . 4 → A. 32

AMASIADO. .... 5 → A. 32

SOLTEIRO .... 6 → A. 32

NS.. . 8 → A. 32

NR.. . 9 → A. 32

A.13d

Há quanto tempo o(a) Sr(a) é divorciado(a)/ separado(a) / viúvo(a)?

UTILIZE A REDAÇÃO CONFORME A SITUAÇÃO

A. 31

MESES ....|___|___|

ANOS ....|___|___|

SEÇÃO A - INFORMAÇÕES PESSOAIS

AGORA QUERO FAZER-LHE ALGUMAS PERGUNTAS SOBRE SUA HISTÓRIA FAMILIAR:

A.18a

Quantos filhos e filhas nascidos vivos o(a) Sr.(a) TEVE?

NÚMERO DE FILHOS: .... |___|___| → **SE 0 (ZERO) VÁ PARA A. 34**

Desses filhos, quantos são PRÓPRIOS ....|___|___|

ENTEADOS.. |___|___|

ADOTIVOS...|___|___|

NS **98**

NR **99**

A. 32

A.18b

Quantos filhos e filhas vivos o(a) Sr.(a) TEM HOJE?

(coloque o número total de filhos e, em seguida, especifique por 'tipo')

NÚMERO TOTAL DE FILHOS VIVOS HOJE: |___|___|

Quantos desses filhos são:

Próprios |___|___|

Enteados |___|___|

Adotivos |___|___|

NS..**98**

NR..**99**

A. 33

A.30

As perguntas A.1 a A 33 foram respondidas...

PELO PRÓPRIO IDOSO **1**

IDOSO + INFORMANTE AUXILIAR **2**

INFORMANTE SUBSTITUTO **3**

A. 34

SEÇÃO B - AVALIAÇÃO COGNITIVA

A SEÇÃO B SÓ DEVE SER RESPONDIDA PELO(A) IDOSO(A).

NESTE ESTUDO ESTAMOS INVESTIGANDO COMO O(A) SR(A) SE SENTE A RESPEITO DE ALGUNS PROBLEMAS DE SAÚDE. GOSTARIAMOS DE COMEÇAR COM ALGUMAS PERGUNTAS SOBRE SUA MEMÓRIA.

B.1

Como o(a) sr(a) avalia sua memória atualmente? (leia as opções)

B. 01

EXCELENTE **1**
MUITO BOA **2**
BOA **3**
REGULAR **4**
MÁ **5**
NS **8**
NR **9**

B.2

Comparando com um ano atrás, o(a) sr(a) diria que agora sua memória está melhor, igual ou pior?

B. 02

MELHOR **1**
IGUAL **2**
PIOR **3**
NS **8**
NR **9**

B.2a

Durante um minuto, diga o nome de todos os animais que conseguir lembrar.

(ANOTE TUDO O QUE O IDOSO FALAR NO PERÍODO DE UM MINUTO)

B. 03

____
____
____
____
____
____
____
____
____
____
____

NR .... **99** [ ]

B.3

Por favor me diga a data de hoje.

PERGUNTE MÊS, DIA, ANO, E DIA DA SEMANA.ANOTE UM PONTO EM CADA RESPOSTA CORRETA

B. 04

		Correto
DIA DO MÊS	|___|___|	[ ]
MÊS	|___|___|	[ ]
ANO	|___|___|	[ ]
DIA DA SEMANA	|___|___|	[ ]
HORA APROXIMADA	|___|___| |___|___|	[ ] → NÃO SOMAR HORA

Segunda feira ... 01
Terça feira 02
Quarta feira 03
Quinta feira 04
Sexta feira 05
Sábado. 06
Domingo 07

TOTAL [ ]

SEÇÃO B - AVALIAÇÃO COGNITIVA

B.4

Agora vou lhe dar o nome de três objetos. Quando eu terminar lhe pedirei que repita em voz alta todas as palavras que puder lembrar, em qualquer ordem. Guarde quais são porque vou voltar a perguntar mais adiante. O Sr. tem alguma pergunta?

LEIA OS NOMES DOS OBJETOS DEVAGAR E DE FORMA CLARA SOMENTE UMA VEZ E ANOTE.

SE O TOTAL É DIFERENTE DE "3":

1) REPITA TODOS OS OBJETOS ATÉ QUE O ENTREVISTADO OS APRENDA. MÁXIMO DE REPETIÇÕES: 5 VEZES
2) ANOTE O NUMERO DE REPETIÇÕES QUE TEVE QUE FAZER
3) NUNCA CORRIJA A PRIMEIRA PARTE
4) ANOTA-SE UM PONTO POR CADA OBJETO LEMBRADO E ZERO PARA OS NÃO LEMBRADOS

B. 05

	Correto
ÁRVORE	
MESA	
CACHORRO	
TOTAL	

Lembrou..........1

Não lembrou....0

NÚMERO DE REPETIÇÕES: .... |______|

(FALE DEVAGAR E OLHANDO DE FRENTE PARA O(A) IDOSO(A).)

B.5

Agora vou dizer alguns números e gostaria que o Sr. repetisse de trás para frente:

1 3 5 7 9

ANOTE A RESPOSTA NO ESPAÇO CORRESPONDENTE.

B. 06

RESPOSTA DO ENTREVISTADO:

9	7	5	3	1

RESPOSTA CORRETA:

ACERTOS

Número de dígitos na ordem correta: [ ]

B.6

Vou lhe dar um papel e quando eu o entregar, apanhe o papel com sua mão direita, dobre-o na metade com as duas mãos e coloque-o sobre suas pernas.

PASSE O PAPEL E ANOTE 1 PONTO PARA CADA AÇÃO CORRETA.

(FALE DEVAGAR E OLHANDO DE FRENTE PARA O(A) IDOSO(A).)

B. 07

AÇÃO CORRETA = 1   AÇÃO INCORRETA = 0

	CORRETO
PEGA O PAPEL COM A MÃO DIREITA.........................	
DOBRA NA METADE COM AS DUAS MÃOS................	
COLOCA O PAPEL SOBRE AS PERNAS.......................	
TOTAL	

B.7

Há alguns minutos, lí uma série de 3 palavras e o(a) sr(a) repetiu as que lembrou. Por favor, diga-me agora quais ainda se lembra.

ANOTE 1 PONTO POR RESPOSTA CORRETA EM QUALQUER ORDEM.

B. 08

	Correto
ÁRVORE	
MESA	
CACHORRO	
TOTAL	

Lembrou........1

Não lembrou....0

SEÇÃO B - AVALIAÇÃO COGNITIVA

B.8

Por favor, copie este desenho.

ENTREGUE AO ENTREVISTADO O DESENHO COM OS CÍRCULOS QUE SE CRUZAM. A AÇÃO ESTÁ CORRETA SE OS CÍRCULOS NÃO SE CRUZAM MAIS DO QUE A METADE. ANOTE UM PONTO SE O DESENHO ESTIVER CORRETO

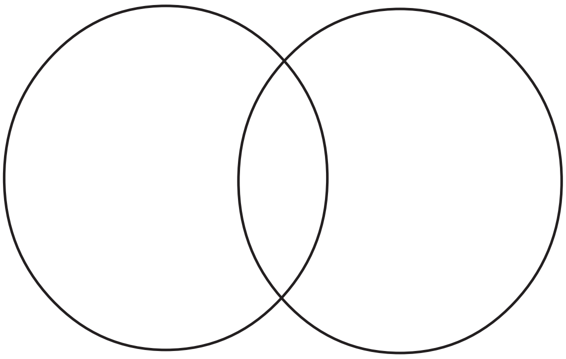

B. 09

Correto [ ]

TOTAL [ ]

B.9 FILTRO

SOME AS RESPOSTAS CORRETAS ANOTADAS NAS PERGUNTAS B.04 A B.09 E ANOTE O TOTAL NA CASELA.

(A PONTUAÇÃO MÁXIMA É 19)

B. 10

TOTAL [ ]

A SOMA É 13 OU MAIS .... **1** → **VÁ PARA B.12**

A SOMA É 12 OU MENOS **2**

B.9a

Se em **B10** a soma **for 12 ou menos** pergunte se há outra pessoa na casa que possa ajudar a responder o questionário a partir da **SEÇÃO C**.

B. 11

HÁ UM INFORMANTE AUXILIAR?

SIM 1

NÃO 2

ANOTE O NOME DO INFORMANTE E APLIQUE A ELE A ESCALA PFEFFER: ______________________

NOME

OBS. AS PERGUNTAS B31, B32 e B12 A B16 DEVEM SER RESPONDIDAS SÓ PELA PESSOA IDOSA.

B.12

Por favor me diga O LOCAL em que o(a) Sr(a) está

ANOTE UM PONTO EM CADA RESPOSTA CORRETA

B. 31

	Correto	
LOCAL DA CASA		(quarto, sala, etc)
LOCAL (CASA OU APTO)		
ENDEREÇO		
CIDADE		
ESTADO		
**TOTAL**		

B.13

Por favor, gostaria que o(a) Sr(a) subtraisse 7 de 100 por cinco vezes.

ANOTE A RESPOSTA NO ESPAÇO CORRESPONDENTE

B. 32

RESPOSTA DO ENTREVISTADO:					
RESPOSTA CORRETA:	93	86	79	72	65

respostas corretas:

OBS. AS PERGUNTAS B12 A B16 DEVEM SER RESPONDIDAS SÓ PELA PESSOA IDOSA.

B.14

Mostrar uma caneta e um relógio e pedir para o(a) idoso(a) nomeá-los

B. 12

Caneta: ☐

Relógio: ☐

CADA RESPOSTA CORRETA = 1 ponto

Total de pontos: ☐

B.15

Agora, vou dizer uma frase e gostaria que, em seguida, o(a) Sr(a) a repetisse:

"NEM AQUI, NEM ALI, NEM LÁ"

FALE DEVAGAR E OLHANDO DE FRENTE PARA O(A) IDOSO(A).

B. 13

RESPOSTA CORRETA = 1 ponto

Total de pontos: ☐

B.16

Agora, vou lhe mostrar uma frase e gostaria que, em seguida, o(a) Sr(a) fizesse o que leu:

Mostre a frase **"FECHE OS OLHOS"**

B. 14

RESPOSTA CORRETA = 1 ponto

Total de pontos: ☐

B.17

Agora, vou lhe **PEDIR PARA ESCREVER** uma frase qualquer:

B. 15

____

____

____

____

Total de pontos: ☐

B.18

Por favor, copie este desenho.

ENTREGUE AO ENTREVISTADO O DESENHO COM OS PENTÁGONOS QUE SE CRUZAM. A AÇÃO ESTÁ CORRETA SE OS PENTÁGONOS NÃO SE CRUZAM MAIS DO QUE A METADE. ANOTE UM PONTO SE O DESENHO ESTIVER CORRETO.

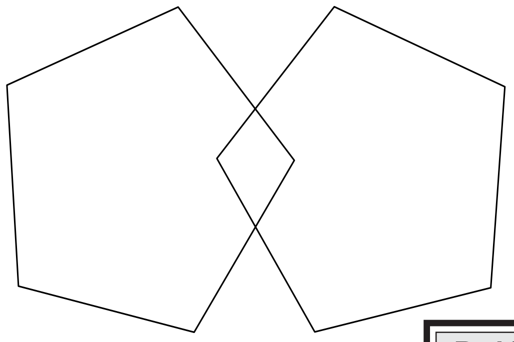

B. 16

Correto ☐

TOTAL ☐

TESTE DO RELÓGIO

ESTE CÍRCULO É UM RELÓGIO. DESENHE OS NÚMEROS QUE HÁ NO RELÓGIO MARCANDO 11 HORAS E 10 MINUTOS.

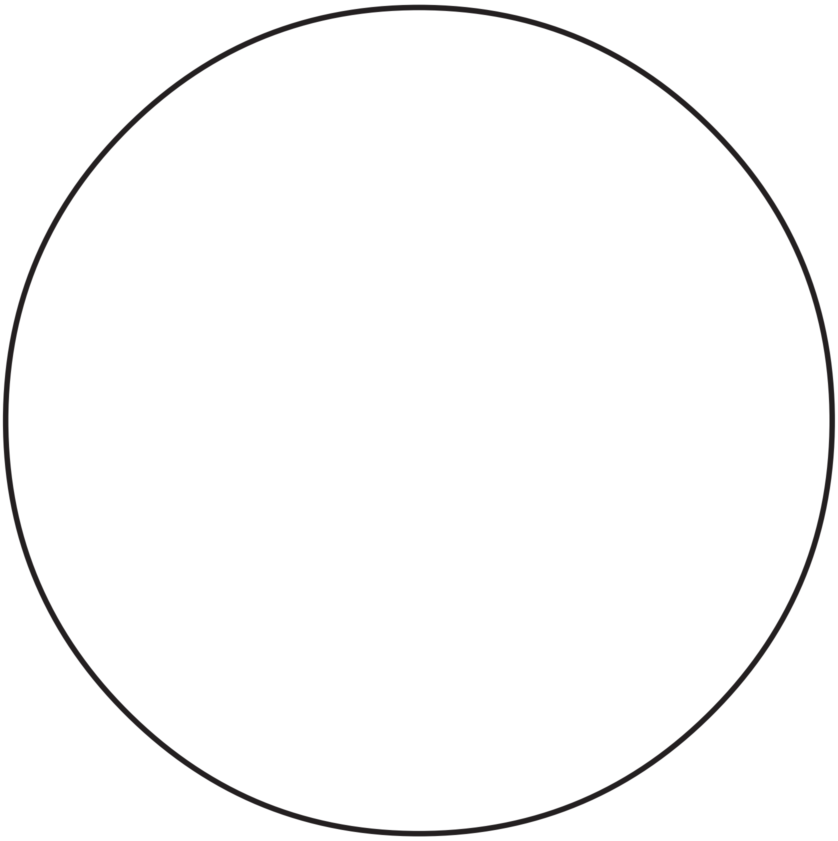

0	Inabilidade absoluta para representar o relógio.
1	O desenho tem algo a ver com o relógio mas com desorganização visuoespacial grave.
2	Desorganização visuoespacial moderada que leva à uma marcação de hora incorreta, perseverança, confusão esquerda-direita, números faltando, números repetidos, sem ponteiros, com ponteiros em excesso.
3	Distribuição visuoespacial correta com marcação errada da hora.
4	Pequenos erros espaciais com digítos e hora corretos.
5	Relógio perfeito.

RESULTADO ☐

OBSERVAÇÕES:

SEÇÃO B - AVALIAÇÃO COGNITIVA (ESCALA PFEFFER)

SE O(A) IDOSO(A) OBTEVE 12 PONTOS OU MENOS NA QUEST. B10 APLICAR ESTA ESCALA.

MOSTRE AO INFORMANTE A SEGUINTE CARTELA COM AS OPÇÕES E LEIA AS PERGUNTAS. ANOTE A PONTUAÇÃO COMO SEGUE:

SIM É CAPAZ	**0**
NUNCA O FEZ, MAS PODERIA FAZER AGORA	**0**
COM ALGUMA DIFICULDADE, MAS FAZ	**1**
NUNCA FEZ E TERIA DIFICULDADE AGORA	**1**
NECESSITA DE AJUDA	**2**
NÃO É CAPAZ	**3**

			Pontuação
B.10a	**(NOME) é capaz de cuidar do seu próprio dinheiro?**	**B. 17**	
B.10b	**(NOME) é capaz de fazer as compras sozinho (por exemplo de comida e roupa)?**	**B. 18**	
B.10c	**(NOME) é capaz de esquentar água para café ou chá e apagar o fogo?**	**B. 19**	
B.10d	**(NOME) é capaz de preparar comida?**	**B. 20**	
B.10e	**(NOME) é capaz de manter-se a par dos acontecimentos e do que se passa na vizinhança?**	**B. 21**	
B.10f	**(NOME) é capaz de prestar atenção, entender e discutir um programa de radio, televisão ou um artigo do jornal?**	**B. 22**	
B.10g	**(NOME) é capaz de lembrar de compromissos e acontecimentos familiares?**	**B. 23**	
B.10h	**(NOME) é capaz de cuidar de seus próprios medicamentos?**	**B. 24**	
B.10i	**(NOME) é capaz de andar pela vizinhança e encontrar o caminho de volta para casa?**	**B. 25**	
B.10j	**(NOME) é capaz de cumprimentar seus amigos adequadamente?**	**B. 26**	
B.10k	**(NOME) é capaz de ficar sozinho(a) em casa sem problemas?**	**B. 27**	

B.11 **FILTRO**

Some os pontos das perguntas B.17 a B.27 e anote na casela denominada "TOTAL".

B. 28

TOTAL

A soma é 6 ou mais.......................... **1** → **CONTINUE A ENTREVISTA COM AJUDA DO INFORMANTE SUBSTITUTO. REVISE A SEÇÃO A**

A soma é 5 ou menos........................ **2** → **CONTINUE A ENTREVISTA COM O ENTREVISTADO. CASO A PESSOA NECESSITE DE AJUDA PARA RESPONDER ALGUMAS PERGUNTAS, CONTINUE COM UM INFORMANTE AUXILIAR**

SEÇÃO C - ESTADO DE SAÚDE

OBSERVAÇÃO:
Somente o(a) idoso(a) pode responder as perguntas C1, C2 e C3. Se ele não puder responder assinale "NR"

C.1

Agora gostaria de lhe fazer algumas perguntas sobre a sua saúde. O(a) Sr(a) diria que sua saúde é ...

C. 01

MUITO BOA	2
BOA	3
REGULAR	4
RUIM	6
MUITO RUIM	7
NS	8
NR	9

C.2

Comparando sua saúde de **HOJE** com a de **DOZE MESES ATRÁS**, o(a) Sr(a) diria que agora sua saúde está...

C. 02

MELHOR	1
IGUAL	2
PIOR	3
NS	8
NR	9

C.3

Comparando-se com outras pessoas de sua idade, o(a) Sr(a) diria que sua saúde é ...

C. 03

MELHOR	1
IGUAL	2
PIOR	3
NS	8
NR	9

C.3a

Pense nas **DUAS ÚLTIMAS SEMANAS** e me diga por quantos dias o(a) Sr(a) deixou de realizar suas atividades habituais por motivo de saúde:

C. 04

NÚMERO DE DIAS	|___|___|
NS	98
NR	99

C.3c

Pense nas **DUAS ÚLTIMAS SEMANAS** e me diga por quantos dias o Sr(a) esteve acamado?

C. 05

NÚMERO DE DIAS	|___|___|
NS	98
NR	99

HIPERTENSÃO

C.4

Alguma vez um médico ou enfermeiro lhe disse que o(a) Sr(a) tem pressão sangüínea alta, quer dizer, HIPERTENSÃO?

C. 06

SIM	1
QUANDO? __________ (ANO)	
NÃO	2
NS	8
NR	9

(NÃO, NS, NR) → **C. 13**

C.4g

Para baixar ou controlar sua pressão sangüínea, o(a) Sr(a):

ASSINALE TODAS AS RESPOSTAS DO(A) IDOSO(A).

C. 07

NÃO FAZ NADA	1
TOMA MEDICAMENTO	2
FAZ DIETA / COME COM POUCO SAL	3
PERDEU PESO	4
FAZ EXERCÍCIOS / ATIV. FÍSICA	5
OUTRO	6
_______________ *Especifique*	
NS	8
NR	9

(2 a 9) **Vá para C. 9**

C.4h

Por quê?

C. 08

NÃO SENTE NECESSIDADE	1
NUNCA FOI ORIENTADO / NÃO SABIA	2
FOI ORIENTADO MAS NÃO GOSTA / NÃO CONSEGUE	3
OUTRO	4
_______________ *Especifique*	
NS	8
NR	9

C.4e

O(a) Sr.(a) costuma ir ao serviço de saúde para controle de sua HIPERTENSÃO ?

C. 09

NUNCA	1
SIM, UMA OU MAIS VEZES AO MÊS	2
SIM, A CADA DOIS OU 3 MESES	3
SIM, A CADA 6 MESES	4
SIM, PELO MENOS UMA VEZ POR ANO	5
QUANDO NÃO ME SINTO BEM	6
NS	8
NR	9

C.4c

Na sua opinião, sua pressão sangüínea geralmente está controlada, ou seja, nos níveis considerados normais?

C. 10

SIM	1
NÃO	2
NS	8
NR	9

C.4d

Em comparação com 12 meses atrás, na sua opinião, seu problema de pressão alta está:

C. 11

MELHOR	1
IGUAL	2
PIOR	3
NS	8
NR	9

C.4f

Alguma vez, o(a) Sr.(a) precisou ser hospitalizado, por PELO MENOS UMA NOITE por causa de sua hipertensão?

C. 12

NUNCA	1
SIM, UMA VEZ	2
SIM, DUAS OU 3 VEZES	3
SIM, MAIS DE 3 VEZES	4
NS	8
NR	9

DIABETES

C.5

Alguma vez um médico ou enfermeiro lhe disse que o(a) Sr(a) tem DIABETES, quer dizer, níveis altos de açúcar no sangue?

C. 13

SIM	1
QUANDO? ______ (ANO)	
NÃO	2
NS	8
NR	9

(NÃO, NS, NR) → **C.32**

C.5a3

Para controlar seu diabetes ou ajudar a baixar os níveis de açúcar no sangue o(a) Sr(a):

C.14

NÃO FAZ NADA	1
FAZ DIETA	2
NÃO INGERE AÇÚCAR / DOCES	3
PERDEU PESO	4
FAZ EXERCÍCIOS / ATIVIDADE FÍSICA	5
OUTRO	6
______ *Especifique*	
NS	8
NR	9

C.5a1

Para tratar seu diabetes o(a) Sr(a) toma...

C. 15

NADA	1
MEDICAMENTO ORAL	2
INSULINA	3
MEDICAÇÃO ORAL E INSULINA	4
NS	8
NR	9

C.5d9

O(a) Sr.(a) costuma verificar/medir sua glicemia, ou seja, o nível de açúcar no seu sangue?

C. 16

SIM:

FAÇO EXAME DE SANGUE NO LABORATÓRIO	1
VERIFICO COM FITAS DOSADORAS NO SANGUE QUE TIRO DO DEDO DA MÃO	3
OUTRO ______ *Especifique*	5
NÃO COSTUMO VERIFICAR	4 → **C.18**
NS	8
NR	9

C.5d10

Com que frequencia o(a) Sr.(a) costuma verificar o nível de seu açucar no sangue?

C. 17

ANTES DE IR À CONSULTA MÉDICA	1	C. 19
QUANDO NÃO ME SI NTO BEM	2	C. 19
DIARIAMENTE	3	C. 19
OUTROS ____ ESPECIFIQUE	4	C. 19
NS	8	C. 19
NR	9	C. 19

C.5d11

Por que o(a) Sr(a) não costuma verificar sua glicemia ou seja os níveis de açúcar no seu sangue?

C. 18

NÃO ACHO NECESSÁRIO	1
NUNCA FUI ORIENTADO / NÃO SABIA	6
FUI ORIENTADO MAS NÃO GOSTA /NÃO CONSEGUE	7
OUTRO ____ Especifique	5
NS	8
NR	9

C.5d

Na sua opinião, seu diabetes, geralmente, está controlado?

C. 19

SIM	1
NÃO	2
NS	8
NR	9

C.5e

Em comparação com 12 meses atrás, na sua opinião, seu diabetes está ...

C.20

MELHOR	1
IGUAL	2
PIOR	3
NS	8
NR	9

C.5e1

Como conseqüência do seu diabetes o(a) Sr.(a) tem (teve) algum problema/ complicação?

ASSINALE TODAS AS ALTERNATIVAS REFERIDAS PELO(A) IDOSO(A).

C. 21

SIM, NOS RINS	1	
SIM, NOS OLHOS	2	C. 23
SIM, DE CIRCULAÇÃO	3	C. 23
SIM, NOS PÉS	4	C. 23
SIM, OUTROS: ____ ESPECIFIQUE	5	C. 23
NÃO	6	C. 23
NS	8	C. 23
NR	9	C. 23

C.5h

O Sr.(a) faz (fez) tratamento para seu problema nos rins?

ANOTAR TODAS AS RESPOSTAS

C. 22

SIM, MEDICAÇÃO/DIETA	1
SIM, DIALISE PERITONIAL	2
SIM, HEMODIÁLISE	3
SIM, TRANSPLANTE	4
NÃO	5
NS	8
NR	9

C.5m

O(a) Sr.(a) sabe que o diabetes pode afetar a sua visão?

C. 23

SIM	1
NÃO	2
NS	8
NR	9

C.5n

Por causa do diabetes o (a) Sr.(a) foi encaminhado ao oculista?

C. 24

SIM	1
NÃO	2
NS	8
NR	9

C.5j

Como conseqüência do seu diabetes o(a) Sr.(a) teve que amputar alguma parte do seu corpo?

C. 25

SIM	1
QUAL? ______________________________	
NÃO	2
NS	8
NR	9

C.5k

Com que frequência o(a) Sr.(a) vai aos serviços de saúde para controlar o seu diabetes?

C. 26

NUNCA	1
SIM, UMA OU MAIS VEZES POR MÊS	2
SIM, A CADA DOIS OU TRÊS MESES	3
SIM, A CADA SEIS MESES.	4
SIM, PELO MENOS UMA VEZ POR ANO	5
SIM, QUANDO ACHO NECESSÁRIO	6
NS	8
NR	9

C.5l

Alguma vez o(a) Sr.(a) precisou ser hospitalizado por **PELO MENOS UMA NOITE** por causa de seu diabetes?

C. 27

NUNCA	1
SIM, UMA VEZ	2
SIM, DUAS OU TRÊS VEZES	3
SIM, MAIS DE TRÊS VEZES	4
NS	8
NR	9

C.5d1

O(A) Sr(a) já apresentou hipoglicemia ou açúcar baixo no sangue?

C. 28

SIM	1	
NÃO	2 →	**C. 30**
NS	8	
NR	9	

C.5d4

O que o(a) Sr(a) fez na última vez que apresentou esse(s) sintomas ?

ANOTAR ATÉ 3 RESPOSTAS.

C. 29

NADA	1
TOMOU LÍQUIDOS COM AÇUCAR OU CHUPOU BALA	2
PROCUROU ASSISTÊNCIA MÉDICA (HOSPITAL, PRONTO-SOCORRO OU POSTO DE SAÚDE	4
ALIMENTOU-SE	3
OUTROS ... *ESPECIFIQUE*	5
NS	8
NR	9

C.5d5

O(A) Sr(a) já apresentou hiperglicemia ou açúcar elevado no sangue?

C. 30

SIM	1	
NÃO	2 →	**C. 32**
NS	8	
NR	9	

C.5d8

O que o(a) Sr(a) fez na última vez que apresentou esse(s) sintomas ?

C. 31

NADA	1
TOMOU MEDICAÇÃO (ORAL OU INSULINA)	2
PROCUROU ASSISTÊNCIA MÉDICA (HOSPITAL, PRONTO-SOCORRO OU POSTO DE SAÚDE	4
OUTROS ... *ESPECIFIQUE*	5
NS	8
NR	9

AGORA GOSTARIA DE LHE FAZER ALGUMAS PERGUNTAS SOBRE SEUS PULMÕES:

C.7

Alguma vez um médico ou enfermeiro lhe disse que tem **ALGUMA DOENÇA CRÔNICA DO PULMÃO**, como **asma, bronquite ou enfisema**?

OBS.: PNEUMONIA e GRIPE NÃO SÃO doenças crônicas.

C. 32

SIM 1

QUAL? ____________ QUANDO? ________ (ANO)

QUAL? ____________ QUANDO? ________ (ANO)

NÃO	2	} **C. 38**
NS	8	
NR	9	

C.7a1

O(a) Sr(a) recebe algum medicamento ou está recebendo algum outro tratamento para sua doença crônica do pulmão?

ASSINALE TODAS AS ALTERNATIVAS CITADAS PELO(A) IDOSO(A).

C. 33

SIM
- OXIGÊNIO CONTÍNUO **1**
- OXIGÊNIO QUANDO NECESSÁRIO **2**

TOMA MEDICAMENTO **3**

FAZ FISIOTERAPIA **4**

OUTRO ______________ **5**
Especifique

NÃO **6**

NS **8**

NR **9**

C.7b

Em comparação com doze meses atrás, sua doença pulmonar está...

C. 34

MELHOR **1**

IGUAL **2**

PIOR **3**

NS **8**

NR **9**

C.7e

Na sua opinião, quanto sua doença pulmonar limita suas atividades diárias como trabalhar ou fazer suas tarefas domésticas?

C. 35

MUITO **1**

POUCO **2**

NÃO INTERFERE **3**

NS **8**

NR **9**

C.7g

Com que frequência o(a) Sr (a) vai aos serviços de saúde por causa de sua doença crônica do pulmão?

C. 36

NUNCA **1**

SIM, VÁRIAS VEZES NA SEMANA **2**

SIM, UMA VEZ NO MÊS **3**

SIM, A CADA DOIS OU TRÊS MESES **4**

SIM, A CADA SEIS MESES **5**

SIM, PELO MENOS UMA VEZ POR ANO. **6**

SIM, QUANDO NECESSÁRIO.. .... **7**

NS **8**

NR **9**

C.7h

Alguma vez o(a) Sr(a) precisou ser hospitalizado por pelo **MENOS UMA NOITE** por causa da sua doença crônica do pulmão?

C. 37

NUNCA **1**

SIM, UMA VEZ.......... **2**

SIM, DUAS OU TRÊS VEZES.......... **3**

SIM, MAIS DE TRÊS VEZES.......... **4**

NS **8**

NR **9**

AGORA GOSTARIA DE LHE FAZER ALGUMAS PERGUNTAS SOBRE SEU CORAÇÃO:

C.8
Alguma vez um médico ou enfermeiro lhe disse que o(a) Sr(a) tem **ALGUM PROBLEMA ou DOENÇA NO CORAÇÃO**, como doença congestiva, doença coronariana ou infarto (ataque do coração)?

QUAL: ___________________________

OBS: excluir dessa pergunta a **HIPERTENSÃO**.

C. 38

SIM	1
QUANDO? ____________________ (ANO) (PARA DOENÇA)	
QUANDO? ____________________ (ANO) (PARA INFARTO)	
NÃO	2
NS	8
NR	9

(NÃO, NS, NR) → **C. 45**

C.8b
Nos últimos 12 meses seu problema de coração está ....

C. 39

MELHOR	1
IGUAL	2
PIOR	3
NS	8
NR	9

C.8c
O(a) Sr(a) toma algum medicamento para seu problema cardíaco?

C. 40

SIM	1
NÃO	2
NS	8
NR	9

C.8d
Com que frequência o(a) Sr (a) vai aos serviços de saúde por causa de seu problema de coração?

C. 41

NUNCA	2
SIM, UMA OU MAIS VEZES POR MÊS	3
SIM, A CADA DOIS OU TRÊS MESES	4
SIM, A CADA SEIS MESES	5
SIM, PELO MENOS UMA VEZ POR ANO	6
SIM, QUANDO NECESSÁRIO	7
NS	8
NR	9

C.8e
Alguma vez o(a) Sr.(a) precisou ser hospitalizado por **PELO MENOS UMA NOITE** por causa de seu problema no coração?

C. 42

NUNCA	6
SIM, UMA VEZ	3
SIM, DUAS OU TRÊS VEZES	4
SIM, MAIS DE TRÊS VEZES	5
NS	8
NR	9

C.8f
Na sua opinião, quanto seu problema de coração interfere no seu trabalho ou em suas atividades domésticas?

C. 43

MUITO	1
POUCO	2
NÃO INTERFERE	3
NS	8
NR	9

C.8g

Em conseqüencia de sua doença cardíaca o(a) Sr(a) fez algum tratamento especial?

OBS.: **CATETERISMO** é um exame; se o(a) idoso(a) fez esse exame e dilatou artérias (colocou stent) assinale **ANGIOPLASTIA**.

C. 44

SIM, ANGIOPLASTIA (DILATAÇÃO ARTERIAL)	1
SIM, COLOCOU MARCA PASSO	2
SIM, CIRURGIA (PONTE, REVASCULARIZAÇÃO) ...	3
SIM, TRANSPLANTE	5
NÃO............	4
NS	8
NR	9

DERRAMES / AVC

C.9

Alguma vez algum médico ou enfermeira disse que o(a) Sr(a) teve um **DERRAME** ou **ISQUEMIA** cerebral (também chamado **EMBOLIA** ou **AVC**? (Incluir **ANEURISMA**).

C. 45

SIM	1
QUANDO? _______________ (ANO DO ÚLTIMO)	
NÃO	2
NS	8
NR	9

NÃO / NS / NR → **C. 55**

C.9c

O(a) Sr(a) ficou com alguma seqüela ou problema derivado do(s) derrame(s) cerebral(is)?

C. 46

SIM	1
NÃO	2
NS	8
NR	9

NÃO / NS / NR → **C. 55**

DEPOIS DO DERRAME, O(A) SENHOR(A) PASSOU A APRESENTAR...

C.9d

Alguma fraqueza nos braços e nas pernas ou dificuldade de movimentar ou usar seus braços ou pernas?

C. 47

SIM	1
NÃO	2
NS	8
NR	9

C.9e

Dificuldade para falar?

C. 48

SIM	1
NÃO	2
NS	8
NR	9

C.9e1

Alguma dificuldade para engolir?

C. 49

SIM, QUALQUER ALIMENTO	1
SIM, APENAS LÍQUIDOS	2
SIM, APENAS SÓLIDOS.	3
NÃO	4
NS	8
NR	9

C.9f

Depois do derrame o(a) Sr(a). passou a ter dificuldade **COM SUA VISÃO?**

C. 50

SIM	1
NÃO	2
NS	8
NR	9

C.9g

Depois do derrame o(a) Sr(a). passou a ter dificuldade para **PENSAR** ou **ENCONTRAR** as palavras certas?

C. 51

SIM	1
NÃO	2
NS	8
NR	9

C.9g1

Depois do derrame o(a) Sr(a). passou a ter dificuldade com a sua **MEMÓRIA**?

C. 52

SIM	1
NÃO	2
NS	8
NR	9

C.9j

Quanto as sequelas ou complicações após o derrame interferem em suas atividades diárias?

C. 53

MUITO	1
POUCO	2
NADA	3
NS	8
NR	9

C.9i

O(a) Sr(a). recebe ou recebeu algum tipo de **TRATAMENTO DE REABILITAÇÃO** por causa do derrame ou suas complicações?

ANOTAR ATÉ 3 RESPOSTAS.

C. 54

SIM, FISIOTERAPIA	3
SIM, TERAPIA OCUPACIONAL	4
SIM, FONOAUDIOLOGIA	5
NÃO	2
NS	8
NR	9

DOENÇA ARTICULAR

C.10

Alguma vez um médico ou enfermeira disse que o(a) Sr(a). tem alguma **DOENÇA ARTICULAR** (nas articulações ou juntas) tais como: artrite, reumatismo, artrose?

OBS.: **Articulação** é a parte do corpo que "**junta**" um osso a outro. Como ela "**dobra**", permite o movimento (ex: **joelhos, cotovelos, tornozelos, punhos**, etc)

C. 55

SIM	1
QUANDO ? __________ (ANO)	
QUE DOENÇA O SR(A) TEM? __________	
NÃO	2
NS	8
NR	9

(NÃO, NS, NR → **C. 62**)

C.10b

Em comparação com 12 meses atrás, sua **DOENÇA ARTICULAR** está.....

C. 56

MELHOR	1
IGUAL	2
PIOR	3
NS	8
NR	9

C.10c

Nos últimos 12 meses o(a) Sr(a) consultou um médico especificamente por causa de sua **DOENÇA ARTICULAR**?

C. 57

SIM	1
NÃO	2
NS	8
NR	9

C.10d

O(a) Sr(a) está tomando algum medicamento ou está recebendo tratamento para sua **DOENÇA ARTICULAR**?

C. 58

SIM	1
NÃO	2 → C.60
NS	8 → C.60
NR	9 → C.60

C.10d1

Foi prescrito pelo médico?

C. 59

SIM	1
NÃO	2
NS	8
NR	9

C.10f

O(a) Sr.(a) sofreu alguma cirurgia por causa de sua **DOENÇA ARTICULAR**?

C. 60

SIM	1
NÃO	2 → C.62
NS	8 → C.62
NR	9 → C.62

C.10g

Qual articulação foi operada?

C. 61

QUADRIL/BACIA	1
JOELHOS	2
TORNOZELOS/PÉS	4
MÃOS / DEDOS	5
OMBRO(S)	6
OUTRO LOCAL ____________ *ESPECIFIQUE*	3
NS	8
NR	9

C.10a2

O(a) Sr.(a) sente **DOR** ou **DESCONFORTO** quando faz algum **ESFORÇO FÍSICO** ou **MOVIMENTO** como por exemplo levantar e andar?

C. 62

SIM	1
DESDE QUANDO? ____________ (ANO)	
NÃO	2 → C.64
NS	8 → C.64
NR	9 → C.64

C.10a3

Em que local o(a) Sr(a). sente dor ou desconforto quando faz esforço físico ou se movimenta?

OBS.: Anotar até no máximo 3 alternativas (as mais importantes).

C. 63

NAS COSTAS (lombalgia/ dor lombar)	1
NO QUADRIL/BACIA	2
NOS JOELHOS	3
NOS TORNOZELOS/PÉS	4
NAS MÃOS	5
NO CORPO TODO	6
NO(S) OMBRO(S)	7
OUTRO LOCAL ____________ *ESPECIFIQUE*	8
NS	98
NR	99

C.10e

Quanto sua **doença articular** e/ou **essa dor** ou **desconforto** interferem em suas atividades diárias ?

C.64

MUITO	1
POUCO	2
NADA	3
NS	8
NR	9

NESTE ESTUDO TAMBÉM ESTAMOS INVESTIGANDO A PRESENÇA DE OUTRAS DORES
<u>QUE NÃO AS RELACIONADAS ÀS ARTICULAÇÕES.</u>
AS PERGUNTAS A SEGUIR DIZEM RESPEITO A QUALQUER OUTRO TIPO DE DOR QUE O(A) SR(A) SINTA.

C.10h

O(a) Sr(a) tem alguma **<u>DOR</u>** há mais de 3 meses, que dói continuamente ou que vai e vem pelo menos uma vez por mês?

<u>EXCLUIR A DOR ARTICULAR</u>

C.65

SIM	1	
NÃO	2	C. 74
NS	8	C. 74
NR	9	C. 74

C.10i

Em que local(ais) o(a) Sr(a) tem essas dores?

OBS.: Anotar até no máximo 5 alternativas (as mais importantes).

C. 66

CABEÇA, FACE E/OU BOCA	1
PESCOÇO	2
OMBROS E BRAÇOS	3
PEITO	4
COSTAS (acima da cintura)	5
COSTAS (na cintura e abaixo-região lombar)	6
ABDOMEM (barriga)	7
PERNAS	8
PELVE	9
REGIÃO ANAL E GENITAL	10
NS	98
NR	99

SE RESPONDER <u>SÓ UMA</u> VÁ PARA C.68

C.10j

De todos esses locais, qual é o **<u>LOCAL DA DOR</u>** que **<u>MAIS</u>** o(a) incomoda?

ASSINALE O MAIS IMPORTANTE (APENAS <u>UM</u> LOCAL)

C. 67

CABEÇA, FACE E/OU BOCA	1
PESCOÇO	2
OMBROS E BRAÇOS	3
PEITO	4
COSTAS (acima da cintura)	5
COSTAS (na cintura e abaixo-região lombar)	6
ABDOMEM (barriga)	7
PERNAS	8
PELVE	9
REGIÃO ANAL E GENITAL	10
NS	98
NR	99

SEÇÃO C - ESTADO DE SAÚDE

C.10k

Há quanto tempo o(a) Sr(a). sente essa dor?

C.68

3 a 6 meses	1
6 meses a 1 ano	2
1 a 2 anos	3
mais de 2 anos	4
NS	8
NR	9

C.10l

Essa dor (a que mais o(a) incomoda) é?

C.69

MUITO FORTE / MUITO INTENSA	1
FORTE / INTENSA	2
MÉDIA / MODERADA	3
FRACA	4
NS	8
NR	9

C.10m

Essa dor (a que mais o(a) incomoda) dói em média?

C.70

QUASE TODOS OS DIAS	1
UMA OU DUAS VEZES POR SEMANA	2
UMA VEZ A CADA 15 DIAS	3
UMA VEZ POR MÊS	4
NS	8
NR	9

C.10n

O que essa **DOR** (a que mais o(a) incomoda) prejudica mais?

OBS.: Anotar até no máximo 3 alternativas (as mais importantes).

C.71

SONO	1
HUMOR	2
ANDAR	3
APETITE	4
LAZER	5
TRABALHO	6
ATIVIDADE SEXUAL	7
RELACIONAMENTO COM AS PESSOAS	8
FAZER COMPRAS / IR AO BANCO	9
CUIDAR DE SI MESMO (tomar banho, vestir-se, alimentar-se, ir ao banheiro)	10
NÃO ATRAPALHA EM NADA	11
OUTROS ______________ *ESPECIFIQUE*	12
NS	98
NR	99

C.10o

Dê uma nota de 0 (zero) a 10 (dez) ao quanto o(a) Sr(a) acha que essa dor o incomoda na atividade citada:

0 (zero) = nenhum prejuízo
10 (dez) = prejuízo total

C.72

NOTA	|___|___|
NS	98
NR	99

C.10p

Para melhorar essa dor (a que mais o incomoda), o(a) Sr(a):

ASSINALE SOMENTE A MAIS IMPORTANTE

C.73

TOMO REMÉDIO(S)	1
APLICO CALOR E FRIO NO LOCAL DA DOR	2
FAÇO MASSAGEM	3
PRATICO EXERCÍCIOS FÍSICOS	4
FAÇO REPOUSO	5
TOMO CHÁ(S)	6
APLICO POMADA(S) / EMPLASTRO	7
OUTRO ______ *ESPECIFIQUE*	8
NS	98
NR	99

OSTEOPOROSE

C.11e

Alguma vez um médico ou enfermeiro disse que o sr. tem **OSTEOPOROSE** (osso fraco)?

C.74

SIM	1	
QUANDO? ______ (ANO)		
NÃO	2	C.76
NS	8	C.76
NR	9	C.76

C. 11e1

O(a) Sr(a) está tomando algum medicamento ou está recebendo tratamento para sua **OSTEOPOROSE?**

C.75

SIM	1
NÃO	2
NS	8
NR	9

DEPRESSÃO

C.20

Alguma vez um médico ou enfermeiro lhe disse que tem algum **PROBLEMA NERVOSO OU PSIQUIÁTRICO**?

(**Excluir depressão**)

C.76

SIM	1	
NÃO	2	C.78
NS	8	C.78
NR	9	C.78

C.20b

O(A) sr(a) está fazendo tratamento para esse problema?

C.77

SIM	1
NÃO	2
NS	8
NR	9

C.20b2

Alguma vez um médico ou enfermeiro lhe disse que tem **DEPRESSÃO**?

C.78

SIM	1	
NÃO	2	C.81
NS	8	C.81
NR	9	C.81

C.20c

Durante os últimos 12 meses, o(a) sr(a) tomou algum remédio para **DEPRESSÃO**?

C.79

SIM	1
NÃO	2
NS	8
NR	9

C.20d

Quanto sua **DEPRESSÃO** interfere em suas atividades do dia-a-dia?

C.80

MUITO	1
POUCO	2
NADA	3
NS	8
NR	9

SEÇÃO C - ESTADO DE SAÚDE - GDS

AGORA PENSE NAS ÚLTIMAS DUAS SEMANAS E DIGA COMO SE SENTIU NA MAIOR PARTE DO TEMPO NESSE PERÍODO...

(APLICAR SOMENTE AO IDOSO)		SIM	NÃO	NS	NR
C.21a O(a) Sr.(a) está basicamente satisfeito com a sua vida?	C.81	1	2	8	9
C.21b Tem diminuído ou abandonado muitos dos seus interesses ou atividades anteriores?	C.82	1	2	8	9
C.21c Sente que sua vida está vazia?	C.83	1	2	8	9
C.21d Tem estado aborrecido freqüentemente?	C.84	1	2	8	9
C.21e Tem estado de bom humor a maior parte do tempo?	C.85	1	2	8	9
C.21f Tem estado preocupado ou tem medo de que alguma coisa ruim vá lhe acontecer?	C.86	1	2	8	9
C.21g Sente-se feliz a maior parte do tempo?	C.87	1	2	8	9
C.21h Com freqüência se sente desamparado ou desvalido?	C.88	1	2	8	9
C.21i Tem preferido ficar em casa em vez de sair e fazer coisas?	C.89	1	2	8	9
C.21j Tem sentido que tem mais problemas com a memória do que outras pessoas de sua idade?	C.90	1	2	8	9
C.21k O(a) sr(a) acredita que é maravilhoso estar vivo?	C.91	1	2	8	9
C.21l Sente-se inútil ou desvalorizado em sua situação atual?	C.92	1	2	8	9
C.21m Sente-se cheio de energia?	C.93	1	2	8	9
C.21n Se sente sem esperança diante da sua situação atual?	C.94	1	2	8	9
C.21o O(a) sr(a) acredita que as outras pessoas estão em situação melhor?	C.95	1	2	8	9

C.21p
As perguntas C81 a C95 foram respondidas C.96

PELO PRÓPRIO IDOSO 1
IDOSO + INFORMANTE AUXILIAR 2
INFORMANTE SUBSTITUTO 3

GOSTARIA AGORA DE LHE FAZER ALGUMAS PERGUNTAS RELACIONADAS À OCORRÊNCIA DE ALGUNS ACIDENTES, ESPECIALMENTE QUEDAS.

C.11_1
O(A) Sr(a) teve alguma queda depois que completou 60 anos? C.97

SIM 1
NÃO 2
NS 8
NR 9

(2, 8, 9 → C.99)

C.11_2<br>**Quantas vezes caiu depois que completou 60 anos?**<br>**C.98**	UMA VEZ .... 1<br>DUAS VEZES .... 2<br>TRÊS VEZES OU MAIS .... 3<br>NS .... 8<br>NR 9
C.11<br>**O(A) Sr(a) teve alguma queda nos últimos 12 meses (último ano)?**<br>**C.99**	SIM .... 1<br>NÃO .... 2<br>NS .... 8<br>NR .... 9<br>(2, 8, 9) → **C. 109**
C.11a<br>**Quantas vezes caiu nos últimos 12 meses (último ano)?**<br>**C.100**	UMA VEZ .... 1<br>DUAS VEZES .... 2<br>TRÊS VEZES OU MAIS .... 3<br>NS .... 8<br>NR 9
C.11a1<br>**Quando foi sua última queda?**<br>**C.101**	HÁ MENOS DE 15 DIAS .... 1<br>15 A 30 DIAS .... 2<br>30 A 90 DIAS .... 3<br>HÁ MAIS DE 90 DIAS .... 4<br>NS .... 8<br>NR 9
C.11a2<br>**Onde o(a) Sr(a) sofreu essa queda?**<br>**C.102**	**EM CASA:** BANHEIRO .... 1<br>QUARTO .... 2<br>COZINHA .... 3<br>SALA .... 4<br>QUINTAL .... 5<br>ESCADA .... 6<br>NA RUA .... 7<br>AO SUBIR/DESCER DO ÔNIBUS/METRÔ .... 8<br>NO INTERIOR DO ÔNIBUS/METRÔ .... 9<br>OUTRO ______ *ESPECIFIQUE* 10<br>NS .... 98<br>NR 99
C.11a3<br>**Como foi essa última queda?**<br>**C.103**	ESCORREGOU .... 1<br>TROPEÇOU .... 2<br>FICOU COM TONTURA .... 3<br>DESMAIOU (SÍNCOPE) .... 4<br>FRAQUEZA NAS PERNAS .... 5<br>FOI EMPURRADO(A) .... 6<br>NÃO LEMBRA .... 7<br>OUTRO ______ *ESPECIFIQUE* 8<br>NS .... 98<br>NR 99

C.11b

Por causa dessa (as) queda (as) o(a) Sr(a) precisou de atendimento médico?

104

SIM	1
NÃO	2
NS	8
NR	9

C.11c

Como conseqüência dessa(s) queda(s) o(a) Sr(a) fraturou o quadril/bacia ou fêmur?
(últimos 12 meses)

C.105

SIM	1
NÃO	2
NS	8
NR	9

C.11d

Como conseqüência dessa(s) queda(s) o(a) Sr(a) fraturou o punho?

C.106

SIM	1
NÃO	2
NS	8
NR	9

C.11d1

Como conseqüência dessa(s) queda(s) o(a) Sr.(a) teve alguma outra fratura?

C.107

SIM	1
ONDE? ________ *ESPECIFIQUE*	
NÃO	2
NS	8
NR	9

C.11d3

Depois de sua(s) queda(s) o(a) Sr(a)?

C.108

FICOU COM MEDO DE CAIR NOVAMENTE	1
SAI MENOS DE CASA POR RECEIO DE CAIR	2
SÓ SAI DE CASA ACOMPANHADO(A)	3
OUTROS ________ *ESPECIFIQUE*	4
NENHUMA DAS ANTERIORES	5
NS	8
NR	9

C.11d2

Desde que o(a) Sr(a) completou 60 anos teve alguma fratura além dessa relatada não relacionada à quedas?
(utilize a redação mais adequada)

C.109

SIM	1
ONDE? ________ *ESPECIFIQUE*	
NÃO	2
NS	8
NR	9

SEÇÃO C - ESTADO DE SAÚDE

MUITAS PESSOAS PERDEM URINA DE VEZ EM QUANDO. ESTAMOS TENTANDO DESCOBRIR, ENTRE AS PESSOAS COM 60 ANOS E MAIS, QUANTAS TÊM ESSE TIPO DE PROBLEMA E O QUANTO ISSO AS ABORRECE. AGORA QUERO QUE O(A) SR(A) ME CONTE SE TEVE/TEM ALGUM PROBLEMA RELACIONADO COM O ATO DE URINAR, PENSANDO NAS ÚLTIMAS QUATRO SEMANAS (ÚLTIMO MÊS):

C.12

Nas últimas 4 semanas, o(a) Sr(a), ALGUMA VEZ, perdeu urina sem querer?

C.110

SIM	1	
NÃO	2	C.115
NS	8	C.115
NR	9	C.115

C.12c1

Com que freqüência o(a) Sr(a) PERDE URINA?

ASSINALE APENAS UMA RESPOSTA

C.111

NUNCA	1	(0 pontos)
UMA VEZ POR SEMANA OU MENOS	2	(1 ponto)
DUAS OU TRÊS VEZES POR SEMANA	3	(2 pontos)
UMA VEZ AO DIA.	4	(3 pontos)
DIVERSAS VEZES AO DIA	5	(4 pontos)
O TEMPO TODO	6	(5 pontos)
NS	8	
NR	9	

C.12c2

Gostariamos de saber a quantidade de urina que o(a) Sr(a) ACREDITA que PERDE.

ASSINALE APENAS UMA RESPOSTA

C.112

NENHUMA	1	(0 pontos)
PEQUENA QUANTIDADE	2	(2 pontos)
MODERADA QUANTIDADE	3	(4 pontos)
GRANDE QUANTIDADE	4	(6 pontos)
NS	8	
NR	9	

C.12c3

Gostariamos de saber o quanto o fato de perder urina interfere em sua vida cotidiana. Para isso gostaria que o(a) Sr(a) desse uma nota de 0(zero) a 10(dez) nessa interferência:

0 (zero) = não interfere em nada
10 (dez) = interfere muito

C.113

CIRCULE A NOTA DADA:

0 1 2 3 4 5 6 7 8 9 10

NS **98**

NR **99**

C.12c18

Por causa de sua perda de urina o(a) Sr(a) necessita usar:

OBS.: Anotar até no máximo 3 alternativas (as mais importantes).

C.114

FRALDA	1
SONDA	2
ABSORVENTES, FORRO DE PROTEÇÃO (uso pessoal)	3
FORRO DE PROTEÇÃO NA CAMA	4
NÃO USA NADA	5
NS	8
NR.	9

C.12c4 **<u>ICQI</u>** ESCORE

Se o(a) idoso responde **"NÃO"** em **C110** marque **00 (zero)** em **C115.**

C.115

SOME OS RESULTADOS DE **C.111 + C.112 + C.113** |___|___|

C.12c7

O(a) Sr(a) tem tido infecções urinárias?

C.116

SIM	1
NÃO	2
NS	8
NR	9

C.12c8

O(a) Sr(a) levanta durante a noite para urinar?

C.117

SIM	1
NÃO	2
DORME COM ABSORVENTE OU FRALDA	3
URINA NA CAMA	4
NS	8
NR	9

AGORA QUERO QUE O(A) SR(A) ME CONTE SE TEVE / TEM ALGUM PROBLEMA RELACIONADO COM O ATO DE EVACUAR:

C.12b

Nos últimos 12 meses, alguma vez perdeu controle dos movimentos intestinais ou das fezes?

C.118

SIM	1	
NÃO	2	C. 121
NS	8	C. 121
NR	9	C. 121

C.12b1

Com que freqüência isso ocorre?

C.119

UMA VEZ POR SEMANA OU MENOS	2
DUAS OU TRÊS VEZES POR SEMANA	1
UMA VEZ AO DIA	3
O TEMPO TODO	4
NS	8
NR	9

C.12b2

Por causa do(s) problema(s) com seu intestino o(a) Sr(a) costuma usar:

OBS.: Anotar até no máximo 3 alternativas (as mais importantes).

C.120

FRALDA	1
FORRO DE PROTEÇÃO (uso pessoal)/ABSORVENTE	3
FORRO DE PROTEÇÃO NA CAMA	4
NÃO USA NADA	5
NS	8
NR.	9

C.12b3

Com que freqüência, quando ficou com vontade de evacuar, o(a) Sr(a) teve dificuldade ou não conseguiu fazê-lo?

C.121

NUNCA	1	→ C.123
UMA VEZ A CADA 15 DIAS	2	
DUAS OU TRÊS VEZES POR SEMANA	3	
UMA VEZ POR SEMANA OU MENOS	4	
DIARIAMENTE	5	
NS	8	
NR	9	

C.12b7

Para ajudá-lo(a) a EVACUAR o(a) Sr(a):

(Assinale todas as alternativas citadas).

C.122

TOMA MEDICAMENTO / LAXANTE	1
FAZ DIETA	2
INGERE MAIOR QUANTIDADE DE ÁGUA/LÍQUIDOS	3
OUTRO	4
NÃO FAZ NADA	5
NS	8
NR	9

CÂNCER

C.6

Alguma vez um médico lhe disse que o(a) Sr(a) tem (teve) câncer (excluir tumores menores da pele)?

C.123

SIM 1
NÃO 2
NS 8
NR 9

(2, 8, 9) → **C.129**

C.6d

Em que ano ou com que idade foi diagnosticado esse câncer?

OBS.: Se teve mais de 1 coloque a **data do último**.

C.124

ANO .... |____|____|____|____| DO ÚLTIMO
IDADE.. |____|____|____| anos
NS **9998**
NR **9999**

C.6d1

Onde era esse câncer?

OBS.: Anotar até no máximo 3 alternativas (as mais importantes).

C.125

ÓRGÃO(S) OU PARTE(S) DO CORPO:

____
____
____
____
____
____

NS.. **98**
NR.. **99**

C.6e

O(a) Sr(a) recebeu algum tratamento para o câncer?

O MAIS RECENTE

C.126

SIM 1
NÃO 2
NS 8
NR 9

(2, 8, 9) → **C.128**

C.6f

Que tipo de tratamento o(a) Sr(a) recebeu para seu câncer mais recente?

ANOTE TODOS OS TIPOS MENCIONADOS ESPONTANEAMENTE

C.127

QUIMIOTERAPIA OU MEDICAÇÃO 1
CIRURGIA OU BIOPSIA 2
RADIAÇÃO/ RAIOS X/ RADIOTERAPIA 3
MEDICAMENTOS PARA OS SINTOMAS (DOR, NÁUSEA, ALERGIAS) 4
OUTRO ______________________________ 5
Especifique
NS 8
NR 9

C.6f1

Esse câncer que o(a) Sr.(a) referiu foi único que teve?

C.128

SIM.. 1
NÃO.. 2
NS 8
NR 9

ANEMIA

C.13a1

Alguma vez algum médico ou enfermeira disse que o(a) Sr(a) tem ou teve ANEMIA?

C.129

SIM **1**

QUANDO? ____________ (ANO)

NÃO **2**

NS **8**

NR **9**

(2, 8, 9 → **C.131**)

C.13a2

O(a) Sr(a) faz ou fez tratamento para ANEMIA?

C.130

SIM, DIETA **1**

SIM, MEDICAÇÃO **2**

SIM, DIETA E MEDICAÇÃO **3**

NÃO **4**

NÃO LEMBRA /NÃO SABE **8**

NR **9**

DST

C.13a3

Alguma vez um médico ou enfermeira lhe disse que o(a) Sr(a) tem alguma doença sexualmente transmissível como sífilis, portador do vírus da AIDS, etc?

C.131

SIM, É PORTADOR DO VÍRUS DA AIDS (HIV+) **1**

SIM, TEM AIDS **2**

DESDE QUANDO? ____________ (ANO)

SIM, TEM OUTRA DOENÇA **3**

____

ESPECIFIQUE

NÃO **4**

NS **8**

NR **9**

(4, 8, 9 → **C.133**)

C.13a4

O(a) Sr(a) fez/faz tratamento para esse problema?

C.132

SIM **1**

NÃO **2**

NS **8**

NR **9**

TRANSPLANTE

C.13a5

O(a) Sr(a) já fez algum transplante (substituição de um órgão do corpo por outro doado por outra pessoa)?

C.133

SIM **1**

DE QUE? ____________

QUANDO? ____________ (ANO)

NÃO **2**

NS **8**

NR **9**

SEÇÃO C - ESTADO DE SAÚDE

AGORA QUERO QUE O(A) SR(A) ME CONTE SE, NOS ÚLTIMOS 12 MESES TEVE ALGUM DOS SEGUINTES PROBLEMAS:

NOS ÚLTIMOS 12 MESES, O(A) SR(A) TEVE....		SIM	NÃO	NS	NR
C.13a **DOR NO PEITO?**	C.134	1	2	8	9
C.13b **INCHAÇO PERSISTENTE NOS PÉS OU TORNOZELOS?**	C.135	1	2	8	9
C.13c **FALTA DE AR QUANDO ESTÁ ACORDADO?**	C.136	1	2	8	9
C.13d **VERTIGEM OU TONTURA PERSISTENTES?**	C.137	1	2	8	9
C.13g **FADIGA OU CANSAÇO IMPORTANTE?**	C.138	1	2	8	9
C.13l **TOSSE PERSISTENTE HÁ MAIS DE 3 SEMANAS?** **OBS:** (Encaminhar ao Posto de Saúde / comunicar a supervisão)	C.139	1	2	8	9
C.13j **NÁUSEA PERSISTENTE E VÔMITO?**	C.140	1	2	8	9
C.13m **FALTA DE APETITE (NÃO SENTE VONTADE DE COMER)?**	C.141	1	2	8	9

C.13

O(a) Sr.(a) sente **ALGUM OUTRO DESCONFORTO OU MAL ESTAR** ou tem **ALGUMA OUTRA DOENÇA** que o(a) incomoda ou interfere no seu dia-a- dia e **QUE NÃO FOI CITADO** até agora?

C.142

SIM **1**

O QUE? ....

____

____

____

NÃO **2**

NS **8**

NR **9**

C.22h_1

Nos últimos **3 MESES**, o(a) Sr(a) **PERDEU** peso sem fazer nenhuma dieta?

C.143

SIM, QUANTOS QUILOS?________________
Especifique

Entre **1 e 3 kg** **1**

Mais de **3 kg** **2**

NÃO **3**

NS **8**

NR **9**

C.22h_2

Nos últimos **12 MESES**, o(a) Sr(a) **PERDEU** peso sem fazer nenhuma dieta?

C.144

SIM, QUANTOS QUILOS?________________
Especifique

ENTRE **1 E 3 KG** **1**

MAIS DE **3 KG** **2**

NÃO **3**

NS **8**

NR **9**

C.22h_3

Nos últimos 12 meses, o(a) Sr(a) **SE SENTE** mais enfraquecido(a), acha que sua **FORÇA DIMINUIU**?

C.145

SIM	1
NÃO	2
NS	8
NR	9

C.22h_4

Atualmente, o(a) Sr(a) faz **MENOS ATIVIDADES FÍSICAS** do que fazia há 12 meses atrás (há um ano).

C.146

SIM	1
NÃO	2
NS	8
NR	9

C.22h_5

Atualmente, o(a) Sr(a) **ACREDITA QUE ESTÁ CAMINHANDO MAIS DEVAGAR** do que há 12 meses atrás (**há um ano / demora mais tempo para fazer o mesmo percurso que fazia antes, como ir ao banco, à igreja, às compras, etc**)?

C.147

SIM	1
NÃO	2
É ACAMADO	3
É CADEIRANTE	4
NS	8
NR	9

NA ÚLTIMA SEMANA...

C.13g_1

Com que frequência, **NA ÚLTIMA SEMANA**, o(a) Sr(a) sentiu que não **CONSEGUIRIA LEVAR ADIANTE SUAS COISAS** (iniciava alguma coisa mas não conseguia terminar):

C.148

NUNCA OU RARAMENTE (MENOS DE 1 DIA)	1
POUCAS VEZES (1-2 DIAS)	2
ALGUMAS VEZES (3-4 DIAS)	3
A MAIOR PARTE DO TEMPO	4
NS	8
NR	9

C.13g_2

Com que frequência, **NA ÚLTIMA SEMANA**, a realização de suas atividades rotineiras exigiram do(a) Sr(a) um grande esforço para serem realizadas:

C.149

RARAMENTE (MENOS DE 1 DIA) OU NUNCA	1
POUCAS VEZES (1-2 DIAS)	2
ALGUMAS VEZES (3-4 DIAS)	3
A MAIOR PARTE DO TEMPO	4
NS	8
NR	9

AGORA GOSTARIA DE SABER ALGUNS DETALHES SOBRE A SAÚDE DOS OLHOS, OUVIDOS E BOCA.

C.14a

O(a) Sr(a). usa óculos ou lentes de contato PARA ENXERGAR DE LONGE?

C.150

SIM 1
NÃO 2
NS 8
NR 9

C.14d

O(a) Sr(a). usa óculos PARA ENXERGAR DE PERTO?

C.151

SIM 1
NÃO 2
NS 8
NR 9

C.14b1

Em geral, **como o(a) Sr(a) classificaria** sua visão:

OBS.: se o(a) idoso usar óculos, peça que ele(a) classifique sua visão considerando o uso do mesmo.

C.152

MUITO BOA 2
BOA 3
REGULAR 4
RUIM 7
MUITO RUIM 10
CEGO. 6
NS 8
NR 9

C.14

Em um ambiente iluminado, peça a(o) idosa(o) para ficar com ambos os olhos abertos e, a uma distância de 3 metros, mostre a prancha com o **OPTOTIPO (E)** nas **<u>4 POSIÇÕES</u>** e peça a ele(a) para identificar cada posição mostrada (conforme o desenho abaixo).

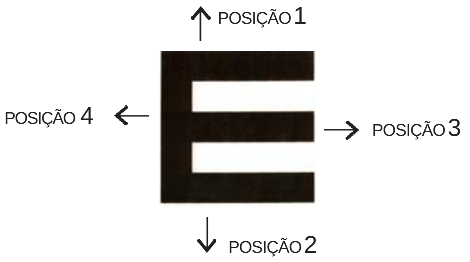

OBS.: se o(a) idoso usar óculos, peça que ele(a) faça o exercício usando o mesmo.

C.153

Assinale um **"X"** em cada posição que for identificada corretamente pelo(a) idoso(a).

POSIÇÃO	1	2	3	4

Acertou 3 ou 4 vezes 1
Acertou 1 ou 2 vezes 2
Não acertou nenhuma vez 3
Não fez o teste 9

C.14f2

Alguma vez um médico ou enfermeira lhe disse que o(a) sr(a) tem/teve **CATARATA**?

C.154

SIM 1
QUANDO? ____________ (ANO)
NÃO 2
NS 8
NR 9
}
Vá para C.156

C.14f

O(a) Sr(a) já foi operado de **CATARATA**?

C.155

SIM	1
OLHO DIREITO (QUANDO?) ________ (ANO)	
OLHO ESQUERDO (QUANDO?) ________ (ANO)	
NÃO	2
NS	8
NR	9

C.14h

Alguma vez um médico ou enfermeira lhe disse que o(a) sr(a) tem **GLAUCOMA** (pressão aumentada no olho)?

C.156

SIM, MAS NÃO FAZ TRATAMENTO	1
SIM E FAZ TRATAMENTO	2
NÃO	3
NS	8
NR	9

C.14h2

Nos últimos 12 meses (último ano), o(a) Sr(a) diria que a sua **VISÃO <u>MELHOROU</u>, <u>ESTÁ IGUAL</u>** ou **<u>PIOROU</u>**?

C.157

MELHOROU	1
IGUAL	2
PIOROU	3
NS	8
NR	9

C15a

Como o(a) Sr(a) CLASSIFICARIA A **SUA AUDIÇÃO**?

OBS.: se o(a) idoso costumar usar aparelho rotineiramente (todos os dias), peça que ele(a) classifique sua audição considerando o uso do mesmo.

C.158

EXCELENTE	1
MUITO BOA	2
BOA	3
REGULAR	4
MÁ	5
SURDO(A)	6
NS	8
NR	9

C.15_1

O(a) Sr(a) usa aparelho auditivo (aparelho para surdez) rotineiramente?

C.159

SIM, ROTINEIRAMENTE	1
SIM, DE VEZ EM QUANDO	2
NÃO (TEM O APARELHO MAS NÃO USA)	3
NÃO (NÃO TEM APARELHO / NÃO PRECISA)	4
NS	8
NR	9

C.18 **FILTRO**<br>**SEXO DO ENTREVISTADO**<br>**C.160**	MULHER .... 1<br>HOMEM .... 2 → **C.172**

SÓ PARA AS MULHERES....

C.18c2<br>A Sra. já fez **MAMOGRAFIA** (radiografia (RX) das mamas) alguma vez?<br>**C.161**	SIM .... 1<br>NUNCA FEZ .... 2<br>NS .... 8<br>NR .... 9<br>(2, 8, 9) **Vá para C.163**
C.18c3<br>Quando foi que a Sra. fez **MAMOGRAFIA** pela última vez?<br>**C.162**	NOS ÚLTIMOS 6 MESES .... 1<br>6 MESES A 12 MESES .... 2<br>12 MESES A 24 MESES .... 3<br>HÁ MAIS DE 2 ANOS .... 4<br>NS .... 8<br>NR 9
C.18d2<br>A Sra. já fez **TESTE DE PAPANICOLAU**, ou seja, o exame para verificar a existência de câncer no colo do útero?<br>**C.163**	SIM .... 1<br>NUNCA FEZ .... 2<br>NS .... 8<br>NR .... 9<br>(2, 8, 9) **Vá para C.165**
C.18d3<br>Quando foi que a Sra. fez esse exame (**PAPANICOLAU)** pela última vez?<br>**C.164**	NOS ÚLTIMOS 6 MESES .... 1<br>6 MESES A 12 MESES .... 2<br>12 MESES A 24 MESES .... 3<br>HÁ MAIS DE 2 ANOS .... 4<br>NS .... 8<br>NR 9
C.18d4<br>A Sra. retornou à **consulta médica** após a realização desses exames (**PAPANICOLAU e ou MAMOGRAFIA**)?<br>**C.165**	SIM .... 1 → **Vá para C.167**<br>NÃO .... 2<br>NS .... 8<br>NR .... 9<br>(8, 9) **Vá para C.167**
C.18d5<br>Por que não retornou?<br>**C.166**	NÃO ACHOU IMPORTANTE / ESQUECEU .... 1<br>NÃO CONSEGUIU AGENDAR .... 2<br>OUTRO MOTIVO .... 3<br>________________<br>*Especifique*<br>NS .... 8<br>NR 9

C.18e<br><br>A senhora fez alguma cirurgia para tirar o útero e/ou os ovários?<br><br>**C.167**	SIM, ÚTERO E OVÁRIOS .... **1**<br>SIM, APENAS ÚTERO .... **2**<br>SIM, APENAS OVÁRIOS .... **4**<br>NÃO .... **3**<br>NS .... **8**<br>NR .... **9**<br>(3, 8, 9) → **C.169**
C.18f<br><br>Que idade tinha quando foi realizada essa cirurgia?<br><br>**C.168**	IDADE .... |____|____| anos<br>NS .... **98**<br>NR **99**
C.18g<br><br>Que idade tinha quando menstruou pela última vez?<br><br>**C.169**	IDADE .... |____|____| anos<br>AINDA MENSTRUA .... **00**<br>NS .... **98**<br>NR **99**
C.18h_1<br><br>Alguma vez a senhora fez REPOSIÇÃO HORMONAL, ou seja, tomou HORMÔNIO de mulher por causa da menopausa?<br><br>**C.170**	SIM, TOMEI, MAS JÁ NÃO TOMO MAIS .... **1**<br>AINDA TOMO .... **2**<br>NUNCA TOMEI .... **3** → **Vá para C.178**<br>NS .... **8**<br>NR **9**
C.18k_1<br><br>Por quanto tempo a senhora fez reposição hormonal?<br><br>**C.171**	NÚMERO DE MESES |____|____| NÚMERO DE anos |____|____| NS .... **98**<br>NR .... **99**<br>→ **C.178**

SÓ PARA OS HOMENS....

C.172 **QUADRO 4: QUESTIONÁRIO I-PSS** (versão em português)

C.31	NENHUMA VEZ	MENOS DE 1 VEZ EM CADA 5	MENOS QUE A METADE DAS VEZES	CERCA DE METADE DAS VEZES	MAIS QUE A METADE DAS VEZES	QUASE SEMPRE	NS	NR
1- No último mês, quantas vezes, em média, o Sr. teve a sensação de não esvaziar completamente a bexiga, depois de terminar de urinar?	0	1	2	3	4	5	8	9
2- No último mês, quantas vezes, em média, o Sr. teve que urinar de novo menos de 2 horas depois de terminar de urinar?	0	1	2	3	4	5	8	9
3- No último mês, quantas vezes, em média, o Sr. notou que parava e recomeçava várias vezes quando urinava?	0	1	2	3	4	5	8	9
4- No último mês, quantas vezes, em média, o Sr. notou que foi difícil conter a vontade de urinar?	0	1	2	3	4	5	8	9
5- No último mês, quantas vezes, em média, o Sr. notou que o jato urinário estava fraco?	0	1	2	3	4	5	8	9
6- No último mês, quantas vezes, em média, o Sr. teve que fazer força para começar a urinar?	0	1	2	3	4	5	8	9

	NENHUMA VEZ	1 VEZ	2 VEZES	3 VEZES	4 VEZES	5 VEZES OU MAIS	NS	NR
7- No último mês, quantas vezes, em média, o Sr. teve que se levantar em cada noite para urinar?	0	1	2	3	4	5	8	9

Escore I-PSS (Escore Internacional de Sintomas Prostáticos): 0 - 35

TOTAL = ______

	ÓTIMO	MUITO BEM	SATISFEITO	MAIS OU MENOS	INSATISFEITO	MAL	PÉSSIMO	NS	NR
8 - Se o sr. tivesse que passar o resto da vida urinando como está agora, como é que o sr. se sentiria?	0	1	2	3	4	5	6	8	9

Escore QI (Qualidade de vida): 0 - 6

C.173

C.19a

O Sr. já fez **EXAME DE PRÓSTATA**?

SIM	1	
NUNCA FEZ	2	Vá para C.178
NS	8	Vá para C.178
NR	9	Vá para C.178

C.174

C.19b

Quando foi que o Sr. fez o **EXAME DE PRÓSTATA** pela última vez?

NOS ÚLTIMOS 6 MESES	1
6 MESES A 12 MESES	2
12 MESES A 24 MESES	3
HÁ MAIS DE 2 ANOS	4
NS	8
NR	9

C.175

C.19c

O Sr. retornou à consulta médica após a realização do **EXAME DE PROSTÁTA**?

SIM	1	Vá para C.177
NÃO	2	
NS	8	Vá para C.177
NR	9	Vá para C.177

C.176

C.19d

Porque a Sr. não retornou?

NÃO ACHOU IMPORTANTE / ESQUECEU	1
NÃO CONSEGUIU AGENDAR	2
OUTRO MOTIVO	3

____
(Especifique)

NS	8
NR	9

C.177

C.19g

O Sr. fez cirurgia para retirar a **PRÓSTATA**?

SIM	1
NÃO	2
NS	8
NR	9

PARA TODOS (homens e mulheres)....

C.19_06

O(a) Sr(a) já tomou **VACINA CONTRA GRIPE**?

C.178

SIM	1	
NÃO	2	Vá para C.180
NS	8	Vá para C.180
NR	9	Vá para C.180

C.19_7

Quando tomou a **VACINA CONTRA GRIPE** pela última vez?

C.179

NOS ÚLTIMOS 6 MESES	1
NOS ÚLTIMOS 12 MESES	2
HÁ MAIS DE 12 MESES	3
NS/NÃO LEMBRA	8
NR	9

C.19_8

O(a) Sr(a) tomou **VACINA CONTRA GRIPE SUÍNA** (**H1N1**)?

C.180

SIM	1
NÃO	2
NS	8
NR	9

C.19_9

O(a) Sr.(a) **TEVE GRIPE** nos últimos 12 meses?

C.181

SIM	1
NÃO	2
NS/NÃO LEMBRA	8
NR	9

C.19_10

O(a) Sr.(a) já tomou **VACINA CONTRA PNEUMONIA**?

C.182

SIM	1
NÃO	2
NS/NÃO LEMBRA	8
NR	9

C.19_12

O(a) Sr.(a) já tomou **VACINA CONTRA TÉTANO**?

C.183

Sim	1	
Não	2	Vá para C.185
NS/Não lembra	8	Vá para C.185
NR	9	Vá para C.185

C.19_13

Quando tomou a **vacina contra tétano** pela última vez?

C.184

Nos últimos 6 meses	1
Nos últimos 12 meses	2
Há mais de 12 meses	4
NS/Não lembra	8
NR	9

PARA TODOS (homens e mulheres)....

SÓ O(A) IDOSO(A)

AGORA GOSTARIA DE LHE FAZER ALGUMAS PERGUNTAS SOBRE A SUA VIDA SEXUAL. A SEXUALIDADE É UMA PARTE NATURAL E IMPORTANTE DE TODAS AS ETAPAS DE NOSSA VIDA. GOSTARIA DE LEMBRÁ-LO(A) QUE TODAS AS SUAS RESPOSTAS SERÃO CONFIDENCIAIS E O SR. PODE SE RECUSAR A RESPONDER.

C.19_1

Para o(a) Sr(a), quanto foi importante sua vida sexual?

(PENSE EM SUA VIDA INTEIRA)

C.185

MUITO IMPORTANTE 1
IMPORTANTE 2
NADA IMPORTANTE 3
NÃO TEVE VIDA SEXUAL ATIVA 4
NS 8
NR 9

C.19_2

Pensando em como se sente agora, em geral, quanto tem sido importante para o(a) Sr(a) a sua vida sexual?

C.186

MUITO IMPORTANTE 1
IMPORTANTE 2
NADA IMPORTANTE 3
NÃO TEM VIDA SEXUAL ATIVA 4
NS 8
NR 9

C.19_3

No último ano o(a) Sr(a) manteve algum tipo de atividade sexual?

C.187

SIM 1
NÃO 2
NS 8
NR 9
(2, 8, 9) → **C.190**

C.19_4

Com que frequência ocorre essa atividade sexual citada?

C.188

2 OU 3 VEZES POR SEMANA 1
UMA VEZ POR SEMANA 2
2 OU 3 VEZES POR MÊS 3
UMA VEZ AO MÊS OU MENOS 4
OUTRO 5
____
(Especifique)
NS 8
NR 9

C.19_6
No último ano quantos parceiro(a)s diferentes o(a) Sr(a) teve?

C.189

|__|__|

C.19_5

Quão satisfeito(a) o(a) Sr(a) diria que está com seu nível de atividade sexual?

C.190

MUITO SATISFEITO(A) 1
SATISFEITO(A) 2
NADA SATISFEITO(A) 3
NS 8
NR 9

SEÇÃO C - ESTADO DE SAÚDE

AGORA VAMOS FALAR DA BOCA E DOS SEUS DENTES.

C.16<br><br>Falta-lhe algum dente?<br><br>**C.191**	SIM .... 1<br>NÃO .... 2<br>NS .... 8<br>NR 9
C.16a<br><br>O Sr(a) consegue contar o número de dentes presentes em sua boca?<br><br>**SÓ O(A) IDOSO(A) RESPONDE.**<br><br>**C.192**	SIM .... 1<br>QUANTOS NO ARCO SUPERIOR? ______<br>QUANTOS NO ARCO INFERIOR? ______<br>NÃO .... 2<br>NS .... 8<br>NR 9
C.16_1<br><br>Porque o(a) Sr(a) acha que perdeu os dentes?<br><br>**SÓ O(A) IDOSO(A) RESPONDE.**<br><br>**C.193**	______<br>______<br>______<br>NS .... 8<br>NR 9
C.16b<br><br>Quando o(a) Sr(a) foi ao dentista pela última vez?<br><br>**C.194**	MENOS DE 1 ANO .... 1<br>DE 1 A 2 ANOS .... 2<br>3 ANOS OU MAIS .... 3<br>NUNCA FOI AO DENTISTA .... 4 → **C.197**<br>NS .... 8<br>NR 9
C.16c<br><br>Para que foi ao dentista pela última vez?<br><br>**C.195**	URGÊNCIA .... 1<br>TRATAMENTO .... 2<br>MANUTENÇÃO .... 3<br>NS .... 8<br>NR 9
C.16c1<br><br>Sua última consulta odontológica foi:<br><br>**C.196**	GRATUITA, NO SERVIÇO PÚBLICO OU SUS .... 1<br>GRATUITA, NO SERV. SOCIAL (SINDICATO, ONG, IGREJA .... 2<br>PAGA, PARTE, DIRETAMENTE NO SERVIÇO SOCIAL .... 3<br>PAGA, INDIRETAMENTEP/PLANO SAÚDE (CONVÊNIO, COOPERATIVA, EMPRESA) .... 4<br>PAGA, EM CONSULTÓRIO / CLÍNICA PRIVADA .... 5<br>NS .... 8<br>NR 9
C.16d<br><br>O(a) Sr(a) diria que sua saúde bucal é muito boa, boa, regular, ruim ou muito ruim?<br><br>**SÓ O(A) IDOSO(A) RESPONDE.**<br><br>**C.197**	MUITO BOA .... 2<br>BOA .... 3<br>REGULAR .... 4<br>RUIM .... 6<br>MUITO RUIM .... 7<br>NS .... 8<br>NR 9

AGORA GOSTARIA QUE ME RESPONDESSE SE O QUE LHE PERGUNTO ACONTECEU SEMPRE, FREQÜENTEMENTE, ALGUMAS VEZES, RARAMENTE OU NUNCA, NOS ÚLTIMOS 12 MESES.

SEMPRE: a situação ocorre nos 7 dias da semana
FREQUENTEMENTE: a situação ocorre de 3 a 6 dias por semana
ALGUMAS VEZES: a situação ocorre de 1 a 2 vezes por semana
RARAMENTE: a situação ocorre menos de 1 vez por semana mas ocorre em algumas ocasiões
NUNCA: a situação não ocorre em nenhum momento

GOHAI

NOS ÚLTIMOS 12 MESES...		Sempre	Freqüen-temente	Algumas vezes	Raramente	Nunca	NS	NR
C.17 Nos últimos 12 meses, com que frequência teve que comer menos ou mudar de comida por causa dos seus dentes, pontes ou dentadura postiça?	C.198	1	2	3	4	5	8	9
C.17a Quantas vezes teve problemas para mastigar comidas duras como carne ou maçã?	C.199	1	2	3	4	5	8	9
C.17b Quantas vezes conseguiu engolir bem?	C.200	1	2	3	4	5	8	9
C.17c Quantas vezes não conseguiu falar bem por causa dos seus dentes ou dentadura?	C.201	1	2	3	4	5	8	9
C.17d Quantas vezes não conseguiu comer as coisas que queria por ter algum problema com seus dentes ou com sua dentadura?	C.202	1	2	3	4	5	8	9
C.17e Quantas vezes não quis sair à rua ou falar com outras pessoas por causa de seus dentes ou da sua dentadura?	C.203	1	2	3	4	5	8	9
C.17f Quando se olha no espelho, quantas vezes esteve contente com seus dentes ou dentadura?	C.204	1	2	3	4	5	8	9
C.17g Quantas vezes teve que usar algum remédio para aliviar a dor de seus dentes ou os problemas na sua boca?	C.205	1	2	3	4	5	8	9
C.17h Quantas vezes esteve preocupado ou se deu conta de que seus dentes ou sua dentadura não estão bem?	C.206	1	2	3	4	5	8	9
C.17i Quantas vezes ficou nervoso por problemas de dentes ou da dentadura?	C.207	1	2	3	4	5	8	9
C.17j Quantas vezes não comeu como queria diante de outras pessoas por causa dos seus dentes ou da dentadura?	C.208	1	2	3	4	5	8	9
C.17k Quantas vezes teve dor nos dentes ou gengivas por causa de alimentos frios, quentes ou doces?	C.209	1	2	3	4	5	8	9

SEÇÃO C - ESTADO DE SAÚDE

OS ESTUDOS FEITOS MOSTRAM QUE A NUTRIÇÃO E O ESTILO DE VIDA SÃO FATORES MUITO IMPORTANTES PARA A SAÚDE. POR ISSO GOSTARIA DE FAZER ALGUMAS PERGUNTAS SOBRE A SUA ALIMENTAÇÃO.

C.22i

Com relação a seu estado nutricional o(a) Sr(a) se considera bem nutrido?

C. 210

SIM	1
NÃO	2
NS	8
NR	9

C.22a

Quantas refeições o(a) Sr(a) faz por dia?

CONSIDERAR O CAFÉ DA MANHÃ COMO UMA REFEIÇÃO

C. 211

UMA	1
DUAS	2
TRÊS OU MAIS	3
NS	8
NR	9

C.22f

Tem comido menos por problemas digestivos ou falta de apetite ou dificuldade de mastigação ou de engolir nos últimos 3 meses?

C. 212

SIM, MUITO	1
SIM, MAIS OU MENOS	3
NÃO	2
NS	8
NR	9

C.22g

Quantos copos ou xícaras de líquido consome diariamente? (incluir água, café, chá, leite, suco etc.)

C. 213

MENOS DE 3 COPOS	1
DE 3 A 5 COPOS	2
MAIS DE 5 COPOS	3
NS	8
NR	9

SEÇÃO C - ESTADO DE SAÚDE

<u>PENSANDO NO ÚLTIMO MÊS</u>, GOSTARIA DE SABER COMO É A SUA ALIMENTAÇÃO.
ENTÃO FAREI PERGUNTAS SOBRE OS ALIMENTOS QUE O(A) SR(A) COSTUMA COMER E BEBER:

**O(A) SR(A) COSTUMA COMER OS SEGUINTES ALIMENTOS? QUANTAS VEZES?**		NÃO COMEU/ COME DE VEZ EM QUANDO.	COME UMA VEZ/ DIA.	COME 2 A 3 VEZE / DIA.	COME PELO MENOS 1 VEZ/SEMANA	COME 2 A 3 VEZES/ SEMANA	COME DE 4 A 6 VEZES / SEMANA	NS	NR
**C. 22_1 Frutas** (banana, laranja, maçã, mamão, melancia, uva, etc).	C.214	1	2	3	4	5	6	8	9
**C. 22_2 Legumes** (abobrinha, cenoura, chuchu, pepino, tomate) e **verduras** (alface, agrião, couve, repolho, rúcula, etc).	C.215	1	2	3	4	5	6	8	9
**C. 22_3 Leite** ou iogurte desnatado, queijos branco *(light).*	C.216	1	2	3	4	5	6	8	9
**C. 22_4 Leite** ou iogurte integral, queijos tipo mussarela, prato, etc.	C.217	1	2	3	4	5	6	8	9
**C. 22_5 Carnes** (de vaca, frango, peixe ou porco, etc).	C.218	1	2	3	4	5	6	8	9
**C. 22_6 Ovos.**	C.219	1	2	3	4	5	6	8	9
**C. 22_7 Feijão,** lentilha, grão de bico, ervilha seca/partida, soja.	C.220	1	2	3	4	5	6	8	9
**C. 22_8 Embutidos** (salsicha, salame, linguiça, mortadela, presunto, peito de peru, *blanquet*, etc).	C.221	1	2	3	4	5	6	8	9
**C. 22_9 Massas e pães (**pão, arroz, macarrão, bolo, aveia, biscoito sem recheio, fubá, farinha de milho/mandioca).	C.222	1	2	3	4	5	6	8	9
**C. 22_10 Cereais integrais** (pão, arroz, bolo, macarrão, biscoito).	C.223	1	2	3	4	5	6	8	9
**C. 22_11 Tubérculos** (batata, inhame, cará, mandioca, mandioquinha, batata doce).	C.224	1	2	3	4	5	6	8	9
**C. 22_12 Doces** (bala, bombom, pirulito, chocolate, bolacha recheada, geléia, pudim, mousse, sorvete, doce de leite, de abóbora, cocada, caldo de cana, refrigerantes não dietéticos, etc).	C.225	1	2	3	4	5	6	8	9
**C. 22_13 Alimentos** *light, diet ou zero* (refrigerante, suco, gelatina, bala/chocolate).	C.226	1	2	3	4	5	6	8	9
**C. 22_14 Gordura vegetal** (óleo de soja, milho, girassol, canola, azeite de oliva, margarina, maionese).	C.227	1	2	3	4	5	6	8	9
**C. 22_15 Gordura animal** (manteiga, nata ou creme de leite, banha de porco, toucinho).	C.228	1	2	3	4	5	6	8	9
**C. 22_16 Frituras** (pastel, batatinha frita, coxinha, quibe, rissóle).	C.229	1	2	3	4	5	6	8	9
**C. 22_17 Alimentos enlatados** (ervilha, milho, azeitona, massa ou molho de tomate).	C.230	1	2	3	4	5	6	8	9
**C. 22_18 Tempero ou molho industrializado** (para saladas, caldos em tablete ou em pó); sopa industrializada, macarrão instantâneo e salgadinhos de pacote.	C.231	1	2	3	4	5	6	8	9

C.23

Nos últimos três meses, em média, quantos dias por semana tomou bebidas alcoólicas? (Por exemplo: cerveja, vinho, aguardente ou outras bebidas que contenham álcool).

C.232

NENHUM	1	→ C.244
MENOS DE 1 DIA POR SEMANA	2	
1 DIA POR SEMANA	3	
2-3 DIAS POR SEMANA	4	
4-6 DIAS POR SEMANA	5	
TODOS OS DIAS	6	
NS	8	
NR	9	

C.23a

Nos últimos três meses, nos dias em que tomou bebida alcoólica, **QUANTOS COPOS** de vinho, cervejas, aguardente ou outra bebida tomou, em média, por dia?

C.233

COPOS DE VINHO	|____|____|
CERVEJAS	|____|____|
OUTRA BEBIDA	|____|____|
NS	98
NR	99

S-MAST-G

VERSÃO GERIÁTRICA DO TESTE MICHIGAN REDUZIDO DE TRIAGEM PARA ALCOOLISMO

		SIM	NÃO
C.23b Quando fala com outras pessoas, o(a) sr(a). sempre subestima quanto bebe?	C.234	1	2
C.23c Depois de algumas doses, o(a) sr(a). às vezes não come ou é capaz de pular uma refeição porque não sente fome?	C.235	1	2
C.23d Tomar algumas doses ajuda a diminuir os seus tremores?	C.236	1	2
C.23e O álcool ás vezes dificulta a lembrança de partes do dia ou da noite?	C.237	1	2
C.23f O(a) sr(a). usualmente toma uma bebida para relaxar ou acalmar os nervos?	C.238	1	2
C.23g O(a) sr(a). bebe para esquecer os seus problemas?	C.239	1	2
C.23h O(a) sr(a). alguma vez aumentou a bebida depois de ter uma perda na sua vida?	C.240	1	2
C.23i Alguma vez um médico ou enfermeira falou que estava preocupado com o seu hábito de beber?	C.241	1	2
C.23j Alguma vez o(a) sr(a). fez regras para administrar o seu hábito de beber?	C.242	1	2
C.23k Quando o(a) sr(a). se sente só, tomar uma bebida melhora?	C.243	1	2

SOME AS RESPOSTAS "SIM" DANDO UM PONTO PARA CADA UMA (0-10) ____________________

C.24

Quanto ao hábito de fumar, o(a) Sr(a)...

LEIA CADA OPÇÃO ATÉ OBTER UMA RESPOSTA AFIRMATIVA.

C.244

FUMA ATUALMENTE	**1**	
JÁ FUMOU, MAS NÃO FUMA MAIS	**2**	→ **C.246**
NUNCA FUMOU	**3**	→ **C.248**
NS	**8**	→ **C.248**
NR	**9**	→ **C.248**

C.24a

Quantos cigarros, charutos ou cachimbos fuma por dia?

DEFINIÇÃO: UM MAÇO = 20 CIGARROS

C.245

CIGARROS POR DIA .... |____|____|

CACHIMBO (*nº de vezes que enche o cachimbo*)|____|____|

CHARUTOS .... |____|____|

Vá para C.247

C.24b

Há quantos anos deixou de fumar?

SE DEIXOU DE FUMAR HÁ MENOS DE UM ANO, ANOTE "00"

C.246

HÁ QUANTOS ANOS: .... |____|____|

IDADE EM ANOS: .... |____|____|____|

ANO .... |____|____|____|____|

NS **9998**

NR **9999**

C.24c

Que idade tinha quando começou a fumar?

C.247

IDADE EM ANOS: .... |____|____|____|

ANO .... |____|____|____|____|

NS **9998**

NR **9999**

Nós estamos interessados em saber como são as suas atividades físicas no dia-a-dia.Suas respostas nos ajudarão a entender quão ativas as pessoas idosas são. As perguntas a seguir dizem respeito ao tempo que o(a) Sr(a) gastou fazendo atividade(s) física(s) na ÚLTIMA SEMANA. Essas perguntas incluem as atividades que o(a) Sr(a) fez no seu trabalho, para ir de um lugar a outro, no lazer, por esporte, por exercício ou como parte das suas atividades em casa ou no jardim.Suas respostas são muito importantes. Por favor responda cada questão mesmo que não se considere ativo(a).

Para responder as questões lembre que:
ATIVIDADES FÍSICAS VIGOROSAS são aquelas que precisam de um grande esforço físico e que fazem RESPIRAR MUITO MAIS FORTE que o normal

ATIVIDADES FÍSICAS MODERADAS são aquelas que precisam de algum esforço físico e que fazem respirar UM POUCO MAIS FORTE que o normal.

C.25c

Quantos dias da última semana o(a) Sr(a) CAMINHOU por **pelo menos 10 minutos contínuos** em casa ou no trabalho, como forma de transporte para ir de um lugar para outro, por lazer, por prazer ou como forma de exercício?

C.248

NÚMERO DE DIAS NA SEMANA: .... |_____| **dias**
NENHUM DIA .... **0** → **C.250**
NS **8**
NR **9**

C.25d

Nesses dias em que o(a) Sr(a) caminhou **por pelo menos 10 minutos contínuos** quanto tempo, **NO TOTAL**, o(a) Sr(a) gastou caminhando por dia?

C.249

NÚMERO DE HORAS: .... |___|___| **horas**
NÚMERO DE MINUTOS .... |___|___| **minutos**
NS **8**
NR **9**

C.25e

Na última semana, QUANTOS DIAS o(a) Sr(a) realizou **ATIVIDADES FÍSICAS MODERADAS** por pelo menos **10 MINUTOS CONTÍNUOS**, ou seja, atividades que exigiram algum **ESFORÇO FÍSICO** e o(a) fizeram respirar **UM POUCO MAIS FORTE** que o normal ou aumentaram um pouco os batimentos de seu coração?

(EXCLUIR A CAMINHADA)

EX: PEDALAR BICICLETA, NADAR, DANÇAR, GINÁSTICA AERÓBICA LEVE, JOGAR VOLEI RECREATIVO, CARREGAR PESOS LEVES, FAZER SERVIÇOS DOMÉSTICOS COMO VARRER, ASPIRAR, CUIDAR DO JARDIM)

C.250

NÚMERO DE DIAS NA SEMANA: .... |_____| DIAS
NENHUM DIA .... **0** → **C.252**
NS **8**
NR **9**

**RELACIONE A(S) ATIVIDADE(S) CITADA(S):**	**CÓDIGO**	**METS**
1) ______	|_|_|_|_|	|_|_|
2) ______	|_|_|_|_|	|_|_|
3) ______	|_|_|_|_|	|_|_|
4) ______	|_|_|_|_|	|_|_|
5) ______	|_|_|_|_|	|_|_|
6) ______	|_|_|_|_|	|_|_|
7) ______	|_|_|_|_|	|_|_|
8) ______	|_|_|_|_|	|_|_|

C.25f

Nesses dias em que o(a) Sr(a) fez essas ATIVIDADES MODERADAS **POR PELO MENOS 10 MINUTOS CONTÍNUOS** quanto tempo, NO TOTAL, o(a) Sr(a) gastou com essas atividades por dia?

C.251

NÚMERO DE HORAS: .... |___|___| **horas**
NÚMERO DE MINUTOS .... |___|___| **minutos**
NS **8**
NR **9**

C.25g

Na última semana, quantos dias o(a) Sr(a) realizou **ATIVIDADES FÍSICAS VIGOROSAS** por pelo menos **10 MINUTOS CONTÍNUOS**, ou seja, atividades que exigiram um grande esforço que aumentaram **MUITO** OS **BATIMENTOS DO SEU CORAÇÃO** e o fizeram **RESPIRAR MUITO MAIS FORTE?**

(EXCLUIR A CAMINHADA)

EX.: CORRER, GINÁSTICA, AERÓBICA, FAZER ESTEIRA, JOGAR FUTUBOL, BASQUETE, CARREGAR GRANDES PESOS, ETC.

C.252

NÚMERO DE DIAS NA SEMANA: .. |_____| DIAS

NENHUM DIA .... **0** → **Vá para C.254**

NS **8**

NR **9**

RELACIONE A(S) ATIVIDADE(S) CITADA(S):

	**CÓDIGO**	**METS**
**1)** ______________________	|__|__|__|__|	|__|__|
**2)** ______________________	|__|__|__|__|	|__|__|
**3)** ______________________	|__|__|__|__|	|__|__|
**4)** ______________________	|__|__|__|__|	|__|__|
**5)** ______________________	|__|__|__|__|	|__|__|

C.25h

Nesses dias em que o(a) Sr(a) fez essas ATIVIDADES VIGOROSAS por pelo menos **10 MINUTOS CONTÍNUOS** quanto tempo, **NO TOTAL**, o(a) Sr(a) gastou com essas atividades por dia?

C.253

NÚMERO DE HORAS: .... **|___|___| horas**

NÚMERO DE MINUTOS .... **|___|___| minutos**

NS **8**

NR **9**

AS PRÓXIMAS QUESTÕES SÃO SOBRE O TEMPO QUE O(A) SR(A) **PERMANECE SENTADO DURANTE O DIA**, NO TRABALHO,EM CASA, DURANTE SEU TEMPO LIVRE. ISSO INCLUI O TEMPO SENTADO LENDO, DESCANSANDO, VISITANDO UM(A) AMIGO(A), ASSISTINDO TELEVISÃO (SENTADO OU DEITADO), USANDO UM COMPUTADOR. **(NÃO INCLUIR O TEMPO SENTADO EM TRANSPORTE DE ÔNIBUS, TREM, METRÔ OU CARRO)**

C.25i

Quanto tempo, no total, o(a) Sr(a) gasta, **SENTADO(A), POR DIA DURANTE A SEMANA**?

C. 254

NÚMERO DE HORAS: .... **|___|___| horas**

NÚMERO DE MINUTOS .... **|___|___| minutos**

NS **8**

NR **9**

C.25j

Quanto tempo, no total, o(a) Sr(a) gasta, SENTADO(A), durante UM DIA DE FINAL DE SEMANA (**SÁBADO OU DOMINGO**)?

C.255

NÚMERO DE HORAS: .... **|___|___| horas**

NÚMERO DE MINUTOS .... **|___|___| minutos**

NS **8**

NR **9**

C.25b

Nos **ÚLTIMOS 12 MESES**, realizou regularmente, para se distrair, algum trabalho manual, artesanato ou atividade artística, pelo menos uma vez por semana?

C.256

SIM **1**

NÃO **2**

NS **8**

NR **9**

C.25k

AS PERGUNTAS DA **SEÇÃO C** FORAM RESPONDIDAS:

C.257

PELO PRÓPRIO IDOSO **1**

IDOSO + INFORMANTE AUXILIAR **2**

INFORMANTE SUBSTITUTO **3**

AS SEGUINTES PERGUNTAS SÃO RELATIVAS AOS SEUS HÁBITOS DE SONO DURANTE O ÚLTIMO MÊS:

C.26

No último mês o(a) Sr(a). teve dificuldade com seu sono?

C.258

SIM	1	
NÃO	2 →	**Vá para C.260**
NS	8	
NR	9	

C.26a

Qual a dificuldade mais frequente:

C.259

DEMORA PARA DORMIR	1
ACORDA DE MADRUGADA E NÃO DORME MAIS	2
ACORDA VÁRIAS VEZES DURANTE A NOITE	3
NÃO CONSEGUE DORMIR	4
OUTROS ____________ *(Especifique)*	5
NS	8
NR	9

C.26b

Durante o último mês, com que frequência o(a) Sr(a) tomou medicamento para ajudá-lo(a) a dormir?

C.260

NENHUMA VEZ	1
MENOS DE 1 VEZ NA SEMANA	2
1 A 2 VEZES NA SEMANA	3
OUTROS ____________ *(Especifique)*	4
TODOS OS DIAS	5

C.26c

No último mês, com que frequência o(a) Sr(a) teve dificuldade de ficar acordado durante o dia?

C.261

NENHUMA VEZ	1
MENOS DE 1 VEZ NA SEMANA	2
1 A 2 VEZES NA SEMANA	3
OUTROS ____________ *(Especifique)*	4
TODOS OS DIAS	5

C.26d

Durante o último mês, de maneira geral, como o(a) Sr(a) classificaria a qualidade do seu sono?

C.262

MUITO BOM	1
BOM	2
REGULAR	3
RUIM	4
MUITO RUIM	5
NS	8
NR	9

C.26e

As perguntas **C26 a C26d** foram respondidas:

C.263

PELO PRÓPRIO IDOSO	1
IDOSO + INFORMANTE AUXILIAR	2
INFORMANTE SUBSTITUTO	3

SEÇÃO C - ESTADO DE SAÚDE

GOSTARIA DE FAZER ALGUMAS PERGUNTAS SOBRE OS PRIMEIROS 15 ANOS DE SUA VIDA.

C.31

Como o(a) Sr(a) descreveria a situação econômica de sua família durante a maior parte dos primeiros 15 anos de sua vida?

LEIA CADA OPÇÃO ATÉ OBTER UMA RESPOSTA AFIRMATIVA.

C.264

BOA	1
REGULAR	2
RUIM	3
NS	8
NR	9

C.32

Durante a maior parte dos primeiros 15 anos de sua vida, o(a) Sr(a) descreveria sua saúde naquela época como excelente, boa ou ruim?

C.265

EXCELENTE	1
BOA	2
RUIM	3
NS	8
NR	9

**Antes dos 15 anos o(a) Sr(a) se lembra de ter tido alguma destas doenças?**		SIM	NÃO	NS	NR
C.28a **Nefrite**	C.266	1	2	8	9
C.28b **Hepatite**	C.267	1	2	8	9
C.28c **Sarampo**	C.268	1	2	8	9
C.28d **Tuberculose**	C.269	1	2	8	9
C.28e **Febre reumática**	C.270	1	2	8	9
C.28f **Asma**	C.271	1	2	8	9
C.28g **Bronquite crônica**	C.272	1	2	8	9
C.28h **Alguma outra?** PERGUNTE SOBRE AS OUTRAS 3 MAIS IMPORTANTES	*Especifique* ______	1	2	8	9
	______	1	2	8	9
	______ C.273	1	2	8	9

C.29

Durante os primeiros 15 anos da sua vida ficou na cama por um mês ou mais devido a algum problema de saúde?

C.274

SIM	1
NÃO	2
NS	8
NR	9

C.30

Durante os primeiros 15 anos da sua vida o(a) Sr(a). diria que houve algum tempo em que não comeu o suficiente ou passou fome?

C.275

SIM	1
NÃO	2
NS	8
NR	9

SEÇÃO D – ESTADO FUNCIONAL

D<br>A pessoa idosa:<br>**D1**	Anda .... 1 Vá para **D3**<br>Está acamada .... 2<br>Usa cadeira de rodas .... 3<br>NS .... 8<br>NR 9
D. 0<br>Pelo fato de estar acamado(a) ou utilizar cadeira de rodas, o(a) idoso(a) desenvolveu algum tipo de ferida na pele? Se sim, marque com um **X** o local da ferida na figura ao lado.<br>**D2**	Sim .... 1<br>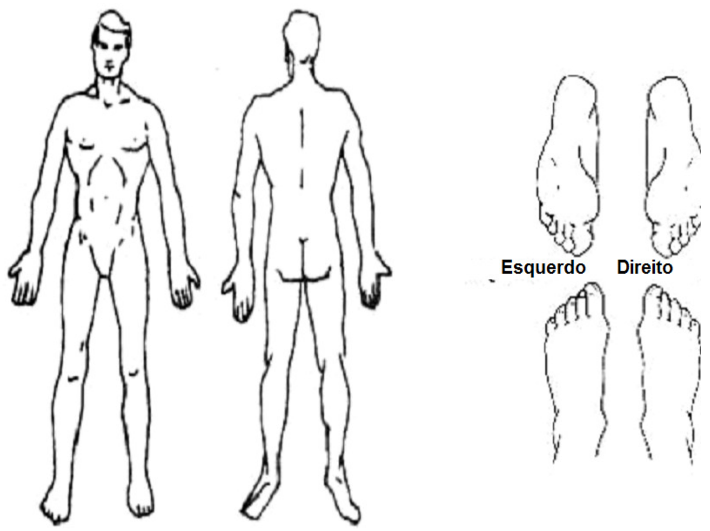<br>Não .... 2<br>NS .... 8<br>NR 9

			SIM	NÃO	NÃO PODE	PODE MAS NÃO FAZ	NS	NR
D.1a	Tem dificuldade para correr ou trotar um quilometro e meio ou 15 quadras?	**D3**	**1**	**2**	**3**	**4**	**8**	**9**
D.1b	Tem dificuldade em caminhar várias ruas (quadras)?	**D4**	**1**	**2**	**3**	**4**	**8**	**9**
D.1c	Tem dificuldade em caminhar uma rua (quadra)?	**D5**	**1**	**2**	**3**	**4**	**8**	**9**
D.2	Tem dificuldade em ficar sentado (a) durante duas horas?	**D6**	**1**	**2**	**3**	**4**	**8**	**9**
D.3	Tem dificuldade em se levantar de uma cadeira depois de ficar sentado(a) durante longos períodos?	**D7**	**1**	**2**	**3**	**4**	**8**	**9**
D.4	Tem dificuldade em subir vários lances de escada sem parar ou descansar?	**D8**	**1**	**2**	**3**	**4**	**8**	**9**
D.5	Tem dificuldade em subir um andar pelas escadas sem parar ou descansar?	**D9**	**1**	**2**	**3**	**4**	**8**	**9**
D.6	Tem dificuldade em se curvar, ajoelhar ou agachar?	**D10**	**1**	**2**	**3**	**4**	**8**	**9**
D.7	Tem dificuldade para estender os braços acima dos ombros?	**D11**	**1**	**2**	**3**	**4**	**8**	**9**
D.8	Tem dificuldade para puxar ou empurrar grandes objetos como, por exemplo, uma poltrona?	**D12**	**1**	**2**	**3**	**4**	**8**	**9**
D.9	Tem dificuldade para levantar ou carregar pesos maiores que 5 kg como, por exemplo, uma sacola de compras pesada?	**D13**	**1**	**2**	**3**	**4**	**8**	**9**
D.10	Tem dificuldade para levantar uma moeda de uma mesa	**D14**	**1**	**2**	**3**	**4**	**8**	**9**

D11a<br>MEDIDA DE INDEPENDENCIA FUNCIONAL<br>Preenchido pela crítica	|____|____| (total)

AGORA VOU FAZER ALGUMAS PERGUNTAS SOBRE AS ATIVIDADES QUE FAZEMOS NO DIA-A-DIA. POR FAVOR, DIGA-ME SE O(A) SR(A) TEM ALGUMA DIFICULDADE PARA EXECUTAR SOZINHO(A) CADA UMA DAS ATIVIDADES QUE EU CITAR DEVIDO A UM PROBLEMA DE SAÚDE.

D.11<br>O(A) Sr(a) tem dificuldade em **ATRAVESSAR UM QUARTO CAMINHANDO?**<br>**D15**	Sim .... 1<br>Não .... 2<br>NS .... 8<br>NR 9
D.11_1<br>O(A) Sr(a) precisa de ajuda para **ATRAVESSAR UM QUARTO CAMINHANDO?**<br>Precisa de supervisão?<br>( ) Sim ( ) Não<br>**D16**	Sim .... 1<br>Não .... 2<br>NS .... 8<br>NR 9
D.12a<br>O(A) Sr(a) costuma utilizar algum **APARELHO OU INSTRUMENTO DE APOIO** para poder atravessar um quarto caminhando**?**<br>**D17**	Sim .... 1<br>Não .... 2<br>NS .... 8<br>NR .... 9<br>(2, 8, 9) Vá para **D21**
D.12b<br>Que tipo de aparelho ou meio de apoio o(a) Sr(a) utiliza**?**<br>**ASSINALAR ATÉ 4 ALTERNATIVAS.**<br>**D18**	Corrimão .... **01**<br>Andador .... **02**<br>Bengala .... **03**<br>Muletas .... **04**<br>Sapatos ortopédicos .... **05**<br>Suporte ou reforço (pernas ou ombro) .... **06**<br>Prótese .... **07**<br>Oxigênio ou respirador .... **08**<br>Móveis ou parede como apoio ..... **09**<br>Cadeira de rodas .... **10**<br>Outro .... **11**<br>*(especifique)*<br>NS .... **98**<br>NR **99**

D.12c<br>O(A) Sr(a) recebe ajuda de alguém para **ATRAVESSAR UM QUARTO CAMINHANDO?**<br>Se SIM:<br>O(A) idoso(a) faz mais de 50% da atividade?<br>( ) Sim ( ) Não<br><br>Quem faz mais esforço?<br>( )idoso(a) ( ) cuidador<br>**D19**	Sim .... 1<br>Não .... 2<br>NS .... 8<br>NR .... 9<br>(2, 8, 9) Vá para **D21**
D.12d<br>Quem o(a) ajuda a **ATRAVESSAR UM QUARTO CAMINHANDO?**<br><br>NOME: ____________________<br><br>Se for referida mais que uma pessoa, anote o nome daquela que ajuda o(a) idoso(a) com mais frequência.<br>**D20**	Cônjuge / Companheiro........ **01**<br>Filho(a) / Enteado(a) .... **02**<br>Pais / Sogros .... **03**<br>Irmão (ãos) .... **04**<br>Genro / Nora .... **05**<br>Neto(s) .... **06**<br>Outro familiar .... **07**<br>Outro NÃO Familiar (não remunerado) .... **08**<br>Cuidador (remunerado) .... **09**<br>Empregada .... **10**<br>NS .... **98**<br>NR **99**
D.13<br>O(A) Sr(a) tem dificuldade para se **VESTIR SOZINHO(A)?**<br>**D21**	Sim, parte de cima do corpo (acima da cintura) .... **1**<br>Sim, parte de baixo do corpo (abaixo da cintura,incluindo meias e sapatos) .... **2**<br>Ambas .... **4**<br>Não .... **3**<br>NS .... **8**<br>NR **9**
D.13_2<br>O(A) Sr(a) precisa de ajuda para se **VESTIR?**<br><br>Precisa de supervisão ou de alguém que separe sua roupa?<br><br>( ) Sim ( ) Não<br>**D22**	Sim .... 1<br>Não .... 2<br>NS .... 8<br>NR .... 9<br>(2, 8, 9) Vá para **D25**
D.13_1<br>O(A) Sr(a) recebe ajuda de alguém para **se VESTIR?**<br><br>Se SIM:<br>O(A) idoso(a) faz mais de 50% da atividade?<br>( ) Sim ( ) Não<br><br>Quem faz mais esforço?<br>( )idoso(a) ( ) cuidador<br>**D23**	Sim .... 1<br>Não .... 2<br>NS .... 8<br>NR .... 9<br>(2, 8, 9) Vá para **D25**

D.13c<br>Quem o(a) ajuda a **se VESTIR?**<br><br>NOME: ______________________________<br><br>Se for referida mais que uma pessoa, anote o nome daquela que ajuda o(a) idoso(a) com mais frequência.<br><br>**D24**	Cônjuge / Companheiro.............................. **01**<br>Filho(a) / Enteado(a) .... **02**<br>Pais / Sogros .... **03**<br>Irmão (ãos) .... **04**<br>Genro / Nora .... **05**<br>Neto(s) .... **06**<br>Outro familiar .... **07**<br>Outro NÃO Familiar (não remunerado) .... **08**<br>Cuidador (remunerado) .... **09**<br>Empregada .... **10**<br>NS .... **98**<br>NR **99**
D.14a<br>O(A) Sr(a) tem dificuldade para **TOMAR BANHO SOZINHO?**<br><br>**D25**	Sim .... **1**<br>Não .... **2**<br>NS .... **8**<br>NR **9**
D.14c2<br>O(A) Sr(a) precisa da ajuda de alguém para **TOMAR BANHO?**<br><br>Precisa de supervisão?<br>( ) Sim ( ) Não<br><br>**D26**	Sim .... 1<br>Não .... 2 }<br>NS .... 8 } **Vá para D29**<br>NR .... 9 }
D.14cd1<br>O(A) Sr(a) recebe ajuda de alguém para **ENTRAR OU SAIR DO CHUVEIRO / BANHEIRA?**<br><br>Se SIM:<br>O(A) idoso(a) faz mais de 50% da atividade?<br>( ) Sim ( ) Não<br><br>Quem faz mais esforço?<br>( )idoso(a) ( ) cuidador<br><br>**D27**	Sim .... **1**<br>Não .... **2** }<br>NS .... **8** } **Vá para D29**<br>NR .... **9** }

D.14d

Quem o(a) ajuda a **TOMAR BANHO/ENTRAR E SAIR DO CHUVEIRO?**

NOME:

____

Se for referida mais que uma pessoa, anote o nome daquela que ajuda o(a) idoso(a) com mais frequência.

D28

Cônjuge / Companheiro..............................	**01**
Filho(a) / Enteado(a)	**02**
Pais / Sogros	**03**
Irmão (ãos)	**04**
Genro / Nora	**05**
Neto(s)	**06**
Outro familiar	**07**
Outro NÃO Familiar (não remunerado)	**08**
Cuidador (remunerado)	**09**
Empregada	**10**
NS	**98**
NR	**99**

D.14b

O(A) Sr(a) costuma utilizar algum **EQUIPAMENTO OU APARELHO** para poder TOMAR BANHO (como corrimão, barra de apoio, etc) ou toma banho **SENTADO(A)** (Cadeira, banquinho, vaso sanitário, etc)**?**

D29

Sim	**1**
Não	**2**
NS	**8**
NR	**9**

D.14a_1a

O(A) Sr(a) tem dificuldade para **FAZER SOZINHO SUA HIGIENE PESSOAL** sozinho, o que inclui:

- Lavar e secar as mãos
- Lavar e secar o rosto
- Escovar os dentes
- Pentear o cabelo
- Barbear-se (homens) ou Maquiar-se (mulheres)

D30

	**Sim**	**Utiliza adaptação**	**Não**	**NS**	**NR**
D14a_1a<br>Lavar e secar as mãos	**1**	**2**	**3**	**8**	**9**
D14a_1b<br>Lavar e secar o rosto	**1**	**2**	**3**	**8**	**9**
D14a_1c<br>Escovar os dentes	**1**	**2**	**3**	**8**	**9**
D14a_1d<br>Pentear o cabelo	**1**	**2**	**3**	**8**	**9**
D14a_1e<br>Barbear-se (homens) ou Maquiar-se (mulheres)	**1**	**2**	**3**	**8**	**9**

D14c02<br>O(A) Sr(a) precisa da ajuda de alguém para **FAZER SUA HIGIENE PESSOAL?**<br>Precisa de supervisão em qualquer um dos itens ou em todos ou precisa que alguém separe ou prepare o material a ser utilizado?<br>( ) Sim ( ) Não	**D31**	Sim .... 1<br>Não .... 2<br>NS .... 8<br>NR .... 9<br>(2, 8, 9) Vá para **D34**
D.14c_1<br>O(A) Sr(a) recebe ajuda de alguém para **FAZER SOZINHO SUA HIGIENE PESSOAL** que inclui:<br>• Lavar e secar as mãos<br>• Lavar e secar o rosto<br>• Escovar os dentes<br>• Pentear o cabelo<br>• Barbear-se (homens) ou Maquiar-se (mulheres)<br>Se SIM:<br>O(A) idoso(a) faz mais de 50% da atividade?<br>( ) Sim ( ) Não<br>Quem faz mais esforço?<br>( )idoso(a) ( ) cuidador	**D32**	Sim .... **1**<br>Não .... **2**<br>NS .... **8**<br>NR .... **9**<br>(2, 8, 9) Vá para **D34**
D.14d_1<br>Quem o ajuda a **FAZER SUA HIGIENE PESSOAL?**<br>NOME: ______________________<br>Se for referida mais que uma pessoa, anote o nome daquela que ajuda o(a) idoso(a) com mais frequência.	**D33**	Cônjuge / Companheiro............ **01**<br>Filho(a) / Enteado(a) .... **02**<br>Pais / Sogros .... **03**<br>Irmão (ãos) .... **04**<br>Genro / Nora .... **05**<br>Neto(s) .... **06**<br>Outro familiar .... **07**<br>Outro NÃO Familiar (não remunerado) .... **08**<br>Cuidador (remunerado) .... **09**<br>Empregada .... **10**<br>NS .... **98**<br>NR **99**

SEÇÃO D – ESTADO FUNCIONAL

D.15a<br>O(A) Sr(a) tem dificuldade para **COMER SOZINHO** a partir de um prato colocado à sua frente**?**<br>**D34**	Sim .... **1**<br>Não .... **2**<br>NS .... **8**<br>NR **9**

D15a1<br>O(A) Sr(a) precisa da ajuda de alguém para **COMER** a partir de um prato colocado à sua frente**?**<br>Precisa de supervisão ou de alguém que corte a comida, encha o copo, etc?<br>( ) Sim ( ) Não<br>**D35**	Sim .... 1<br>Não .... 2<br>NS .... 8<br>NR .... 9<br>(Não, NS, NR) Vá para **D38**

D.15b<br>O(A) Sr(a) recebe ajuda de alguém para **COMER?**<br>Se SIM:<br>O(A) idoso(a) faz mais de 50% da atividade?<br>( ) Sim ( ) Não<br>Quem faz mais esforço?<br>( )idoso(a) ( ) cuidador<br>**D36**	Sim .... **1**<br>Não .... **2**<br>NS .... **8**<br>NR .... **9**<br>(Não, NS, NR) Vá para **D38**

D.15c<br>Quem o(a) ajuda a **COMER?**<br>NOME: ______________________________<br>Se for referida mais que uma pessoa, anote o nome daquela que ajuda o(a) idoso(a) com mais frequência.<br>**D37**	Cônjuge / Companheiro........ **01**<br>Filho(a) / Enteado(a) .... **02**<br>Pais / Sogros .... **03**<br>Irmão (ãos) .... **04**<br>Genro / Nora .... **05**<br>Neto(s) .... **06**<br>Outro familiar .... **07**<br>Outro NÃO Familiar (não remunerado) .... **08**<br>Cuidador (remunerado) .... **09**<br>Empregada .... **10**<br>NS .... **98**<br>NR **99**

D.16b<br>Para **DEITAR E/OU LEVANTAR DA CAMA; SENTAR E/OU LEVANTAR DA CADEIRA/CADEIRA DE RODAS**, O(A) Sr(a) costuma utilizar algum **APOIO** (bengala, andador, móveis próximos, parede, etc)?<br>**D38**	Sim .... **1**<br>Não .... **2**<br>Não se aplica (é acamado) .... **3**<br>NS .... **8**<br>NR **9**

D.16a<br>O(A) Sr(a) tem dificuldade para **DEITAR E/OU LEVANTAR DA CAMA; SENTAR E/OU LEVANTAR DA CADEIRA/CADEIRA DE RODAS SOZINHO(A)?**<br>**D39**	Sim .... **1**<br>Não .... **2**<br>Não se aplica<br>(é acamado) .... **3**<br>NS .... **8**<br>NR **9**
D.16a1<br>O(A) Sr(a) precisa da ajuda de alguém para **DEITAR E/OU LEVANTAR DA CAMA; SENTAR E/OU LEVANTAR DA CADEIRA/CADEIRA DE RODAS?**<br>Precisa de supervisão ?<br>( ) Sim ( ) Não<br>**D40**	Sim .... 1<br>Não .... 2<br>NS .... 8<br>NR .... 9<br>(2, 8, 9) Vá para **D43**
D.16c<br>O(A) Sr(a) recebe ajuda de alguém para **DEITAR E/OU LEVANTAR DA CAMA; SENTAR E/OU LEVANTAR DA CADEIRA/CADEIRA DE RODAS?**<br><br>**PARA OS <u>ACAMADOS</u> CONSIDERAR A AJUDA PARA MOVÊ-LO(A) NA CAMA**<br><br>Se SIM:<br>O(A) idoso(a) faz mais de 50% da atividade?<br>( ) Sim ( ) Não<br>Quem faz mais esforço?<br>( )idoso(a) ( ) cuidador<br>**D41**	Sim .... **1**<br>Não .... **2**<br>NS .... **8**<br>NR .... **9**<br>(2, 8, 9) Vá para **D43**
D.16D<br><br>Quem o(a) ajuda a **DEITAR E/OU LEVANTAR DA CAMA; SENTAR E/OU LEVANTAR DA CADEIRA/CADEIRA DE RODAS?**<br><br>NOME:<br>______________________________<br><br>Se for referida mais que uma pessoa, anote o nome daquela que ajuda o(a) idoso(a) com mais frequência.<br>**D42**	Cônjuge / Companheiro.............................. **01**<br>Filho(a) / Enteado(a) .... **02**<br>Pais / Sogros .... **03**<br>Irmão (ãos) .... **04**<br>Genro / Nora .... **05**<br>Neto(s) .... **06**<br>Outro familiar .... **07**<br>Outro NÃO Familiar (não remunerado) .... **08**<br>Cuidador (remunerado) .... **09**<br>Empregada .... **10**<br>NS .... **98**<br>NR **99**

Pergunta	Respostas
D.17b<br><br>Para **IR AO BANHEIRO inclui: (Ir ao banheiro, despir-se, sentar nio vaso sanitário, limpar-se, levantar do vaso sanitário, vestir-se lavar as mãos)**, o(a) Sr(a) costuma utilizar algum **APOIO** (bengala, andador, móveis próximos, parede, etc)?<br>**D43**	Sim .... **1**<br>Não .... **2**<br>Não se aplica (é acamado ou cadeirante)........ **3**<br>NS .... **8**<br>NR **9**
D.17a<br>O(A) Sr(a) tem dificuldade para **IR AO BANHEIRO SOZINHO(A) (incluindo sentar e levantar do vaso sanitário)?**<br>**D44**	Sim .... **1**<br>Não .... **2**<br>Não se aplica<br>(é acamado ou cadeirante) .... **3**<br>NS .... **8**<br>NR **9**
D.17a1<br>O(A) Sr(a) precisa de ajuda para **IR AO BANHEIRO?**<br>Precisa de supervisão ?<br>( ) Sim ( ) Não<br>**D45**	Sim .... 1<br>Não .... 2<br>NS .... 8<br>NR .... 9<br>(2, 8, 9) Vá para **D48**
D.17c<br>O(A) Sr(a) recebe ajuda de alguém para **IR AO BANHEIRO (incluindo sentar e levantar do vaso sanitário)?**<br><br>**PARA OS <u>ACAMADOS</u> E CADEIRANTES CONSIDERAR A AJUDA PARA TROCÁ-LOS (FRALDA, ROUPA DE CAMA, ROUPAS, ETC)**<br><br>Se SIM:<br>O(A) idoso(a) faz mais de 50% da atividade?<br>( ) Sim ( ) Não<br><br>Quem faz mais esforço?<br>( )idoso(a) ( ) cuidador<br>**D46**	Sim .... **1**<br>Não .... **2**<br>NS .... **8**<br>NR .... **9**<br>(2, 8, 9) Vá para **D48**

Pergunta	Respostas
D.17d<br><br>Quem o(a) ajuda a **IR AO BANHEIRO (incluindo sentar e levantar do vaso sanitário)?**<br><br>**PARA OS <u>ACAMADOS</u> E CADEIRANTES CONSIDERAR A AJUDA PARA TROCÁ-LOS (FRALDA, ROUPA DE CAMA, ROUPAS, ETC)**<br><br>NOME:<br>______________________________<br><br>Se for referida mais que uma pessoa, anote o nome daquela que ajuda o(a) idoso(a) com mais frequência.<br>**D47**	Cônjuge / Companheiro.............................. **01**<br>Filho(a) / Enteado(a) .... **02**<br>Pais / Sogros .... **03**<br>Irmão (ãos) .... **04**<br>Genro / Nora .... **05**<br>Neto(s) .... **06**<br>Outro familiar .... **07**<br>Outro NÃO Familiar (não remunerado) .... **08**<br>Cuidador (remunerado) .... **09**<br>Empregada .... **10**<br>NS .... **98**<br>NR **99**
D.18a<br>O(A) Sr(a) tem dificuldade para **PREPARAR UMA REFEIÇÃO QUENTE?**<br>**D48**	Sim .... **1**<br>Não costuma fazer .... **2**<br>Não .... **3**<br>NS .... **8**<br>NR **9**
D.18a1<br>O(A) Sr(a) precisa de ajuda para<br>**PREPARAR UMA REFEIÇÃO QUENTE?**<br>**D49**	Sim .... 1<br>Não .... 2 }<br>NS .... 8 } Vá para **D52**<br>NR .... 9 }
D.18b<br>O(A) Sr(a) recebe ajuda de alguém para<br>**PREPARAR UMA REFEIÇÃO QUENTE?**<br>**D50**	Sim .... **1**<br>Não .... **2** }<br>NS .... **8** } Vá para **D52**<br>NR .... **9** }

D.18c<br><br>Quem o(a) ajuda a **PREPARAR UMA REFEIÇÃO QUENTE?**<br><br>NOME:<br>______________________________<br><br>Se for referida mais que uma pessoa, anote o nome daquela que ajuda o(a) idoso(a) com mais frequência.<br><br>**D51**	Cônjuge / Companheiro.............................. **01**<br>Filho(a) / Enteado(a) .... **02**<br>Pais / Sogros .... **03**<br>Irmão (ãos) .... **04**<br>Genro / Nora .... **05**<br>Neto(s) .... **06**<br>Outro familiar .... **07**<br>Outro NÃO Familiar (não remunerado) .... **08**<br>Cuidador (remunerado) .... **09**<br>Empregada .... **10**<br>NS .... **98**<br>NR **99**
D.19a<br>O(A) Sr(a) tem dificuldade para **CUIDAR DE SEU PRÓPRIO DINHEIRO?**<br><br>**D52**	Sim .... **1**<br>Não costuma fazer .... **2**<br>Não .... **3**<br>NS .... **8**<br>NR **9**
D.19a1<br>O(A) Sr(a) precisa de ajuda para **CUIDAR DE SEU DINHEIRO?**<br><br>**D53**	Sim .... 1<br>Não .... 2<br>NS .... 8<br>NR .... 9<br>(2, 8, 9) Vá para **D56**
D.19b<br>O(A) Sr(a) recebe ajuda de alguém para **CUIDAR DE SEU DINHEIRO?**<br><br>**D54**	Sim .... **1**<br>Não .... **2**<br>NS .... **8**<br>NR .... **9**<br>(2, 8, 9) Vá para **D56**

Pergunta	Respostas
D.19c<br>Quem o(a) ajuda a **CUIDAR DE SEU DINHEIRO?**<br>NOME:<br>______________________________<br>Se for referida mais que uma pessoa, anote o nome daquela que ajuda o(a) idoso(a) com mais frequência.<br>**D55**	Cônjuge / Companheiro.............................. **01**<br>Filho(a) / Enteado(a) .... **02**<br>Pais / Sogros .... **03**<br>Irmão (ãos) .... **04**<br>Genro / Nora .... **05**<br>Neto(s) .... **06**<br>Outro familiar .... **07**<br>Outro NÃO Familiar (não remunerado) .... **08**<br>Cuidador (remunerado) .... **09**<br>Empregada .... **10**<br>NS .... **98**<br>NR **99**
D.19l<br>O(A) Sr(a) **COSTUMA UTILIZAR O CAIXA ELETRÔNICO DO BANCO** (para retirar dinheiro, pagar contas, etc)**?**<br>**D56**	Não .... **1**<br>SIM, **SEM** AJUDA .... **2**<br>SIM, **COM** AJUDA .. .. **3**<br>NS .... **8**<br>NR .... **9**<br>(2, 3, 8, 9) **Vá para D58**
D.19m<br>Por que **<u>NÃO COSTUMA</u> UTILIZAR O CAIXA ELETRÔNICO DO BANCO** (para retirar dinheiro, pagar contas, etc)**?**<br>**D57**	Não gosto .... **1**<br>Não estou acostumado(a) .... **2**<br>Tenho dificuldade .. .. **3**<br>Não preciso .... **4**<br>Outros.. **5**<br>*(especifique)*<br>NS .... **8**<br>NR **9**
D.20a<br>O(A) Sr(a) tem dificuldade para **UTILIZAR ALGUM TIPO DE TRANSPORTE SOZINHO COMO ÔNIBUS, TAXI, METRÔ, ETC** para ir a outros lugares como igreja, médico, etc**?**<br>**D58**	Sim .... **1**<br>Não costuma fazer .... **2**<br>Não .... **3**<br>NS .... **8**<br>NR **9**

D.20a1<br>O(A) Sr(a) precisa de ajuda para **UTILIZAR ALGUM TIPO DE TRANSPORTE COMO ÔNIBUS, TAXI, METRÔ, ETC?**<br>**D59**	Sim .... 1<br>Não .... 2<br>NS .... 8<br>NR .... 9<br>(2, 8, 9) Vá para **D62**
D.20b<br>Alguém costuma auxiliá-lo(a) a subir ou descer de um transporte ou lhe oferece transporte quando necessita ou ainda o(a) ajuda a consegui-lo (chamar um taxi, por exemplo)**?**<br>**D60**	Sim .... **1**<br>Não .... **2**<br>NS .... **8**<br>NR .... **9**<br>(2, 8, 9) **Vá p/ D 62**
D.20c<br>Quem o(a) ajuda a **UTILIZAR ALGUM TIPO DE TRANSPORTE?**<br>NOME: ______________________________<br>Se for referida mais que uma pessoa, anote o nome daquela que ajuda o(a) idoso(a) com mais frequência.<br>**D61**	Cônjuge / Companheiro............................ **01**<br>Filho(a) / Enteado(a) .... **02**<br>Pais / Sogros .... **03**<br>Irmão (ãos) .... **04**<br>Genro / Nora .... **05**<br>Neto(s) .... **06**<br>Outro familiar .... **07**<br>Outro NÃO Familiar (não remunerado) .... **08**<br>Cuidador (remunerado) .... **09**<br>Empregada .... **10**<br>NS .... **98**<br>NR **99**

D.21a<br>O(A) Sr(a) tem dificuldade para **FAZER COMPRAS SOZINHO** (alimentos, produtos de limpeza, roupas, remédios, etc**?**<br>**D62**	Sim .... **1**<br>Não costuma fazer .... **2**<br>Não .... **3**<br>NS .... **8**<br>NR **9**

D.21a1<br>O(A) Sr(a) precisa de ajuda para **FAZER COMPRAS?**<br>**D63**	Sim .... 1<br>Não .... 2<br>NS .... 8<br>NR .... 9<br>(2, 8, 9) Vá para **D66**
D.21b<br>O(A) Sr(a) recebe ajuda de alguém para **FAZER ESSAS COMPRAS?**<br>**D64**	Sim .... **1**<br>Não .... **2**<br>NS .... **8**<br>NR .... **9**<br>(2, 8, 9) Vá para **D66**
D.21c<br>Quem o(a) ajuda a **FAZER ESSAS COMPRAS?**<br>NOME:<br>_______________________________<br>Se for referida mais que uma pessoa, anote o nome daquela que ajuda o(a) idoso(a) com mais frequência.<br>**D65**	Cônjuge / Companheiro........ **01**<br>Filho(a) / Enteado(a) .... **02**<br>Pais / Sogros .... **03**<br>Irmão (ãos) .... **04**<br>Genro / Nora .... **05**<br>Neto(s) .... **06**<br>Outro familiar .... **07**<br>Outro NÃO Familiar (não remunerado) .... **08**<br>Cuidador (remunerado) .... **09**<br>Empregada .... **10**<br>NS .... **98**<br>NR **99**
D.22a<br>O(A) Sr(a) tem dificuldade para **UTILIZAR O TELEFONE FIXO OU ORELHÃO?**<br>**D66**	Sim .... **1**<br>Não tem telefone em casa .... **2**<br>Não .... **3**<br>NS .... **8**<br>NR **9**

D.22a1<br>O(A) Sr(a) precisa de ajuda para **UTILIZAR O TELEFONE FIXO OU ORELHÃO?**<br>**D67**	Sim .... 1<br>Não .... 2<br>NS .... 8<br>NR .... 9<br>(2, 8, 9) Vá para **D70**
D.22b<br>O(A) Sr(a) recebe ajuda de alguém quando precisa ou quer **UTILIZAR O TELEFONE FIXO OU ORELHÃO?**<br>**D68**	Sim .... **1**<br>Não .... **2**<br>NS .... **8**<br>NR .... **9**<br>(2, 8, 9) Vá para **D70**
D.22c<br>Quem o(a) ajuda a **UTILIZAR O TELEFONE FIXO OU ORELHÃO?**<br>NOME:<br>______________________________<br>Se for referida mais que uma pessoa, anote o nome daquela que ajuda o(a) idoso(a) com mais frequência.<br>**D69**	Cônjuge / Companheiro........ **01**<br>Filho(a) / Enteado(a) .... **02**<br>Pais / Sogros .... **03**<br>Irmão (ãos) .... **04**<br>Genro / Nora .... **05**<br>Neto(s) .... **06**<br>Outro familiar .... **07**<br>Outro NÃO Familiar (não remunerado) .... **08**<br>Cuidador (remunerado) .... **09**<br>Empregada .... **10**<br>NS .... **98**<br>NR **99**
D.22l<br>O(A) Sr(a) **COSTUMA UTILIZAR TELEFONE CELULAR?**<br>**D70**	Não .... **1**<br>SIM, **SEM** AJUDA .... **2**<br>SIM, **COM** AJUDA .. .. **3**<br>NS .... **8**<br>NR .... **9**<br>(2, 3, 8, 9) **Vá para D72**

D.22m<br>Por que **<u>NÃO COSTUMA</u> UTILIZAR TELEFONE CELULAR?**<br>**D71**	Não gosto .... **1**<br>Não estou acostumado(a) .... **2**<br>Tenho dificuldade .. .. **3**<br>Não preciso .... **4**<br>Não tenho telefone celular .... **5**<br>Outros.. **6**<br>*(especifique)*<br>NS .... **8**<br>NR **9**
D.23a<br>O(A) Sr(a) tem dificuldade para fazer **TAREFAS DOMÉSTICAS LEVES** como arrumar a cama, tirar pó dos móveis, etc**?**<br>**D72**	Sim .... **1**<br>Não costuma fazer .... **2**<br>Não .... **3**<br>NS .... **8**<br>NR **9**
D.23a1<br>O(A) Sr(a) precisa de ajuda para fazer **TAREFAS DOMÉSTICAS LEVES?**<br>**D73**	Sim .... 1<br>Não .... 2<br>NS .... 8<br>NR .... 9<br>(2, 8, 9) Vá para **D76**
D.23b<br>O(A) Sr(a) recebe ajuda de alguém para fazer **TAREFAS DOMÉSTICAS LEVES?**<br>**D74**	Sim .... **1**<br>Não .... **2**<br>NS .... **8**<br>NR .... **9**<br>(2, 8, 9) Vá para **D76**

D.23c<br><br>Quem o(a) ajuda a fazer **TAREFAS DOMÉSTICAS LEVES?**<br><br>NOME:<br>____________________________<br><br>Se for referida mais que uma pessoa, anote o nome daquela que ajuda o(a) idoso(a) com mais frequência.<br><br>**D75**	Cônjuge / Companheiro.............................. **01**<br>Filho(a) / Enteado(a) .... **02**<br>Pais / Sogros .... **03**<br>Irmão (ãos) .... **04**<br>Genro / Nora .... **05**<br>Neto(s) .... **06**<br>Outro familiar .... **07**<br>Outro NÃO Familiar (não remunerado) .... **08**<br>Cuidador (remunerado) .... **09**<br>Empregada/diarista .... **10**<br>NS .... **98**<br>NR **99**
D.24a<br>O(A) Sr(a) tem dificuldade para fazer **TAREFAS DOMÉSTICAS PESADAS** como limpar o chão, lavar o banheiro, lavar o quintal, etc**?**<br><br>**D76**	Sim .... **1**<br>Não costuma fazer .... **2**<br>Não .... **3**<br>NS .... **8**<br>NR **9**
D.24a1<br>O(A) Sr(a) precisa de ajuda para fazer **TAREFAS DOMÉSTICAS PESADAS?**<br><br>**D77**	Sim .... 1<br>Não .... 2 }<br>NS .... 8 } Vá para **D80**<br>NR .... 9 }
D.24b<br>O(A) Sr(a) recebe ajuda de alguém para fazer **TAREFAS DOMÉSTICAS PESADAS?**<br><br>**D78**	Sim .... **1**<br>Não .... **2** }<br>NS .... **8** } Vá para **D80**<br>NR .... **9** }

D.24c<br><br>Quem o(a) ajuda a fazer **TAREFAS DOMÉSTICAS PESADAS?**<br><br>NOME:<br>______________________________<br><br>Se for referida mais que uma pessoa, anote o nome daquela que ajuda o(a) idoso(a) com mais frequência.<br><br>**D79**	Cônjuge / Companheiro.............................. **01**<br>Filho(a) / Enteado(a) .... **02**<br>Pais / Sogros .... **03**<br>Irmão (ãos) .... **04**<br>Genro / Nora .... **05**<br>Neto(s) .... **06**<br>Outro familiar .... **07**<br>Outro NÃO Familiar (não remunerado) .... **08**<br>Cuidador (remunerado) .... **09**<br>Empregada/diarista .... **10**<br>NS .... **98**<br>NR **99**
D.25a<br>O(A) Sr(a) tem dificuldade para **TOMAR SEUS REMÉDIOS SOZINHO?**<br><br>**D80**	Sim .... **1**<br>Não .... **2**<br>Não toma remédios .... **3** Vá p/ **D85**<br>NS .... **8**<br>NR **9**
D.25a1<br>O(A) Sr(a) precisa de ajuda para **TOMAR SEUS REMÉDIOS?**<br><br>**D81**	Sim .... 1<br>Não .... 2 }<br>NS .... 8 } Vá para **D85**<br>NR .... 9 }
D.25b<br>O(A) Sr(a) recebe ajuda de alguém para **TOMAR SEUS REMÉDIOS?**<br><br>**D82**	Sim .... **1**<br>Não .... **2** }<br>NS .... **8** } Vá para **D84**<br>NR .... **9** }
D.25c<br><br>Quem o(a) ajuda a **TOMAR SEUS REMÉDIOS?**<br><br>NOME:<br>______________________________<br><br>Se for referida mais que uma pessoa, anote o nome daquela que ajuda o(a) idoso(a) com mais frequência.<br><br>**D83**	Cônjuge / Companheiro.............................. **01**<br>Filho(a) / Enteado(a) .... **02**<br>Pais / Sogros .... **03**<br>Irmão (ãos) .... **04**<br>Genro / Nora .... **05**<br>Neto(s) .... **06**<br>Outro familiar .... **07**<br>Outro NÃO Familiar (não remunerado) .... **08**<br>Cuidador (remunerado) .... **09**<br>Empregada .... **10**<br>NS .... **98**<br>NR .... **99**<br>} **Vá p/ D85**

D.25l<br>Como o(a) Sr(A) faz para **TOMAR SEUS REMÉDIOS?**<br>**D84**	Não toma .... **1**<br>Alguém deixa separado .... **2**<br>Outros .... **3**<br>*(especifique)*<br>NS .... **8**<br>NR **9**
D.26<br>NÚMERO DE <u>**CUIDADORES DIFERENTES**</u> MENCIONADOS PELO(A) IDOSO(A) QUE O(A) AUXILIAM EM SUAS DIFICULDADES<br>**D85**	NENHUM .... **1**<br>UM .... **2**<br>DOIS .... **3**<br>TRÊS .... **4**<br>QUATRO OU MAIS .... **5**<br>NÃO SE APLICA (não mencionou dificuldades) ... **6**<br>NS .... **8**<br>NR **9**
D.1<br>AS PERGUNTAS DA SEÇÃO D FORAM <u>**RESPONDIDAS**</u><br>**D86**	PELO(A) PRÓPRIO(A) IDOSO (A) .... **1**<br>IDOSO(A) + INFORMANTE AUXILIAR .... **2**<br>INFORMANTE SUBSTITUTO **3**

OBSERVAÇÕES:

____
____
____
____
____
____
____
____
____
____
____
____
____

"AVALIAÇÃO DA QUALIDADE DE VIDA SF-12" (SÓ PARA O IDOSO)

OS SEGUINTES ITENS SÃO SOBRE ATIVIDADES QUE O(A) SR.(A) PODERIA FAZER ATUALMENTE DURANTE UM DIA COMUM. **DEVIDO A SUA SAÚDE**, O(A) SR.(A) TERIA DIFICULDADE PARA FAZER ESSAS ATIVIDADES? NESTE CASO, QUANTO? (circule um número para cada linha)**!**

ATIVIDADES	SIM DIFICULTA MUITO	SIM DIFICULTA UM POUCO	NÃO NÃO DIFICULTA DE MODO ALGUM	NS	NR
D29a. **Atividades moderadas**, tais como mover uma mesa, passar aspirador de pó, jogar bola, varrer a casa	1	2	3	8	9
D29b. Subir **vários** lances de escada	1	2	3	8	9

DURANTE AS **ÚLTIMAS QUATRO SEMANAS (Último mês)**, O(A) SR.(A) TEVE ALGUM DOS SEGUINTES PROBLEMAS COM O SEU TRABALHO OU COM ALGUMA ATIVIDADE DIÁRIA REGULAR, **COMO CONSEQUÊNCIA DE SUA SAÚDE FÍSICA?** (circule uma em cada linha)

	Sim	Não	NS	NR
D.29c **Realizou menos** tarefas do que gostaria?	1	2	8	9
D.29d Esteve limitado no seu **tipo** de trabalho ou em outras atividades	1	2	8	9

DURANTE AS **ÚLTIMAS QUATRO SEMANAS (Último mês)**, O(A) SR.(A) TEVE ALGUM DOS SEGUINTES PROBLEMAS COM O SEU TRABALHO OU COM ALGUMA ATIVIDADE DIÁRIA REGULAR, **COMO CONSEQUÊNCIA DE ALGUM PROBLEMA EMOCIONAL** (COMO SENTIR-SE DEPRIMIDO OU ANSIOSO)? (circule uma em cada linha)

	Sim	Não	NS	NR
D.29e **Realizou menos** tarefas do que gostaria?	1	2	8	9
D.29f Não trabalhou ou não fez qualquer das atividades com tanto **cuidado** como geralmente faz?	1	2	8	9

SEÇÃO D – ESTADO FUNCIONAL

DURANTE AS **ÚLTIMAS QUATRO SEMANAS (Último mês)**, QUANTO A **<u>DOR</u>** INTERFERIU COM O SEU TRABALHO NORMAL (INCLUINDO TANTO O TRABALHO, FORA DE CASA E DENTRO DE CASA)? (circule uma)

**D.29g**	**De maneira alguma**	**Um pouco**	**Moderadamente**	**Bastante**	**Extremamente**	**NR**
	**1**	**2**	**3**	**4**	**5**	**9**

ESTAS QUESTÕES SÃO SOBRE COMO O(A) SR.(A) SE SENTE E COMO TUDO TEM ACONTECIDO COM O(A) SR.(A) DURANTE AS **ÚLTIMAS QUATRO SEMANAS (Último mês)**. PARA CADA QUESTÃO, POR FAVOR DÊ UMA RESPOSTA QUE MAIS SE APROXIME DA MANEIRA COMO O(A) SR.(A) SE SENTE EM RELAÇÃO AS ÚLTIMAS QUATRO SEMANAS. (circule um número para cada linha)

	**TODO TEMPO**	**A MAIOR PARTE DO TEMPO**	**UMA BOA PARTE DO TEMPO**	**ALGUMA PARTE DO TEMPO**	**UMA PEQUENA PARTE DO TEMPO**	**NUNCA**	**NS**	**NR**
D.29h. Quanto tempo o(a) Sr.(a) tem se sentido calmo ou tranqüilo?	**1**	**2**	**3**	**4**	**5**	**6**	**8**	**9**
D.29i Quanto tempo o(a) Sr.(a) tem se sentido com muita energia?	**1**	**2**	**3**	**4**	**5**	**6**	**8**	**9**
D.29j Quanto tempo o(a) Sr.(a) tem se sentido desanimado e abatido?	**1**	**2**	**3**	**4**	**5**	**6**	**8**	**9**

DURANTE AS **ÚLTIMAS QUATRO SEMANAS (Último mês**, QUANTO DO SEU TEMPO A SUA SAÚDE FÍSICA OU PROBLEMAS EMOCIONAIS INTERFERIRAM **<u>NAS SUAS ATIVIDADES SOCIAIS</u>** (COMO VISITAR AMIGOS, PARENTES, ETC)? (circule uma)

D.29K	**TODO TEMPO**	**A MAIOR PARTE DO TEMPO**	**UMA BOA PARTE DO TEMPO**	**ALGUMA PARTE DO TEMPO**	**UMA PEQUENA PARTE DO TEMPO**	**NUNCA**	**NS**	**NR**
	**1**	**2**	**3**	**4**	**5**	**6**	**8**	**9**

D.27 - AVALIAÇÃO DAS ATIVIDADES AVANÇADAS DE VIDA DIÁRIA
(DEVE SER RESPONDIDO PELO(A) PRÓPRIO(A) IDOSO(A))

Vou fazer algumas perguntas relacionadas à algumas atividades.
Gostaria de saber se o(a) Sr(a) as executa e os motivos pelos quais as faz ou não.

D.27_1a

Mantém contato com outras pessoas por meio de cartas, telefone ou email?

SIM	1	
NÃO	2	VÁ p/ 27_1c
NS	8	VÁ p/ 27_2a
NR	9	

D.27_1b

Gostaria de saber qual o principal motivo pelo qual o(s) Sr(a) **FAZ** essa atividade

LAZER	1	Vá para 27_1d
TRABALHO	2	
CONTATO SOCIAL	3	
ATIVIDADE FÍSICA/EXERCÍCIO	4	
OUTROS ______ ESPECIFIQUE	5	
NS	8	
NR	9	

D.27_1c

Gostaria de saber qual o motivo pelo qual o(s) Sr(a) **NÃO FAZ** essa atividade

NÃO TENHO INTERESSE NELA/NÃO GOSTO	1
LIMITAÇÃO FUNCIONAL	2
NECESSITO DE AJUDA PARA EXECUTÁ-LA, MAS NÃO A TENHO	3
OUTROS ______ ESPECIFIQUE	4
NS	8
NR	9

D.27_1d

Quanto o(a) Sr(a) se sente limitado para executar essa atividade

NADA	1
UM POUCO/ ÀS VEZES	2
MUITO / TOTALMENTE	3
NS	8
NR	9

D.27_2a

Visita seus amigos e familiares em suas casas

SIM	1	
NÃO	2	VÁ p/ 27_2c
NS	8	VÁ p/ 27_3a
NR	9	

D.27_2b

Gostaria de saber qual o principal motivo pelo qual o(s) Sr(a) **FAZ** essa atividade

LAZER	1	Vá para 27_2d
TRABALHO	2	
CONTATO SOCIAL	3	
ATIVIDADE FÍSICA/EXERCÍCIO	4	
OUTROS ______ ESPECIFIQUE	5	
NS	8	
NR	9	

D.27_2c

Gostaria de saber qual o motivo pelo qual o(s) Sr(a) **NÃO FAZ** essa atividade

NÃO TENHO INTERESSE NELA/NÃO GOSTO	1
LIMITAÇÃO FUNCIONAL	2
NECESSITO DE AJUDA PARA EXECUTÁ-LA, MAS NÃO A TENHO	3
OUTROS ______ ESPECIFIQUE	4
NS	8
NR	9

D.27_2d

Quanto o(a) Sr(a) se sente limitado para executar essa atividade

NADA	1
UM POUCO/ ÀS VEZES	2
MUITO / TOTALMENTE	3
NS	8
NR	9

D.27_3a

Cuida ou provê assistência a outras pessoas (incluindo cuidado pessoal, transporte, compras para familiares ou amigos)

SIM	1	
NÃO	2	VÁ p/ 27_3c
NS	8	VÁ p/ 27_4a
NR	9	

D.27_3b

Gostaria de saber qual o principal motivo pelo qual o(s) Sr(a) **FAZ** essa atividade

LAZER	1	Vá para 27_3d
TRABALHO	2	
CONTATO SOCIAL	3	
ATIVIDADE FÍSICA/EXERCÍCIO	4	
OUTROS ______ ESPECIFIQUE	5	
NS	8	
NR	9	

D.27_3c

Gostaria de saber qual o motivo pelo qual o(s) Sr(a) **NÃO FAZ** essa atividade

NÃO TENHO INTERESSE NELA/NÃO GOSTO	1
LIMITAÇÃO FUNCIONAL	2
NECESSITO DE AJUDA PARA EXECUTÁ-LA, MAS NÃO A TENHO	3
OUTROS ______ ESPECIFIQUE	4
NS	8
NR	9

D.27_3d

Quanto o(a) Sr(a) se sente limitado para executar essa atividade

NADA	1
UM POUCO/ ÀS VEZES	2
MUITO / TOTALMENTE	3
NS	8
NR	9

D.27_4a

Cuida dos afazeres domésticos de sua casa (limpeza e arrumação da casa, lavagem de roupas, pequenos reparos da casa.

SIM	1	
NÃO	2	VÁ p/ 27_4c
NS	8	VÁ p/ 27_5a
NR	9	VÁ p/ 27_5a

D.27_4b

Gostaria de saber qual o principal motivo pelo qual o(s) Sr(a) **FAZ** essa atividade

LAZER	1	Vá para 27_4d
TRABALHO	2	
CONTATO SOCIAL	3	
ATIVIDADE FÍSICA/EXERCÍCIO	4	
OUTROS ______ ESPECIFIQUE	5	
NS	8	
NR	9	

D.27_4c

Gostaria de saber qual o motivo pelo qual o(s) Sr(a) **NÃO FAZ** essa atividade

NÃO TENHO INTERESSE NELA/NÃO GOSTO	1
LIMITAÇÃO FUNCIONAL	2
NECESSITO DE AJUDA PARA EXECUTÁ-LA, MAS NÃO A TENHO	3
OUTROS ______ ESPECIFIQUE	4
NS	8
NR	9

D.27_4d

Quanto o(a) Sr(a) se sente limitado para executar essa atividade

NADA	1
UM POUCO/ ÀS VEZES	2
MUITO / TOTALMENTE	3
NS	8
NR	9

D.27_5a

Trabalha como voluntário fora de casa?

SIM	1	
NÃO	2 →	VÁ p/ 27_5c
NS	8	VÁ p/ 27_6a
NR	9	VÁ p/ 27_6a

D.27_5b

Gostaria de saber qual o principal motivo pelo qual o(s) Sr(a) **FAZ** essa atividade

LAZER	1	Vá para 27_5d
TRABALHO	2	Vá para 27_5d
CONTATO SOCIAL	3	Vá para 27_5d
ATIVIDADE FÍSICA/EXERCÍCIO	4	Vá para 27_5d
OUTROS ____ ESPECIFIQUE	5	Vá para 27_5d
NS	8	Vá para 27_5d
NR	9	Vá para 27_5d

D.27_5c

Gostaria de saber qual o motivo pelo qual o(s) Sr(a) **NÃO FAZ** essa atividade

NÃO TENHO INTERESSE NELA/NÃO GOSTO	1
LIMITAÇÃO FUNCIONAL	2
NECESSITO DE AJUDA PARA EXECUTÁ-LA, MAS NÃO A TENHO	3
OUTROS ____ ESPECIFIQUE	4
NS	8
NR	9

D.27_5d

Quanto o(a) Sr(a) se sente limitado para executar essa atividade

NADA	1
UM POUCO/ ÀS VEZES	2
MUITO / TOTALMENTE	3
NS	8
NR	9

D.27_6a

Cuida dos próprios negócios e finanças (dinheiro, pagamento de contas, negocia com o senhorio ou inquilino, com empresas ou agências governamentais?

?

SIM	1	
NÃO	2 →	VÁ p/ 27_6c
NS	8	VÁ p/ 27_7a
NR	9	VÁ p/ 27_7a

D.27_6b

Gostaria de saber qual o principal motivo pelo qual o(s) Sr(a) **FAZ** essa atividade

LAZER	1	Vá para 27_6d
TRABALHO	2	Vá para 27_6d
CONTATO SOCIAL	3	Vá para 27_6d
ATIVIDADE FÍSICA/EXERCÍCIO	4	Vá para 27_6d
OUTROS ____ ESPECIFIQUE	5	Vá para 27_6d
NS	8	Vá para 27_6d
NR	9	Vá para 27_6d

D.27_6c

Gostaria de saber qual o motivo pelo qual o(s) Sr(a) **NÃO FAZ** essa atividade

NÃO TENHO INTERESSE NELA/NÃO GOSTO	1
LIMITAÇÃO FUNCIONAL	2
NECESSITO DE AJUDA PARA EXECUTÁ-LA, MAS NÃO A TENHO	3
OUTROS ________ ESPECIFIQUE	4
NS	8
NR	9

D.27_6d

Quanto o(a) Sr(a) se sente limitado para executar essa atividade

NADA	1
UM POUCO/ ÀS VEZES	2
MUITO / TOTALMENTE	3
NS	8
NR	9

D.27_7a

Cuida da própria saúde (inclui o manejo diário das medicações, seguimento de dieta especial, agendamento de consultas médicas).

SIM	1	
NÃO	2	→ **VÁ p/ 27_7c**
NS	8	**VÁ p/ 27_8a**
NR	9	

?

D.27_7b

Gostaria de saber qual o principal motivo pelo qual o(s) Sr(a) **FAZ** essa atividade

LAZER	1	**Vá para 27_7d**
TRABALHO	2	
CONTATO SOCIAL	3	
ATIVIDADE FÍSICA/EXERCÍCIO	4	
OUTROS ________ ESPECIFIQUE	5	
NS	8	
NR	9	

D.27_7c

Gostaria de saber qual o motivo pelo qual o(s) Sr(a) **NÃO FAZ** essa atividade

NÃO TENHO INTERESSE NELA/NÃO GOSTO	1
LIMITAÇÃO FUNCIONAL	2
NECESSITO DE AJUDA PARA EXECUTÁ-LA, MAS NÃO A TENHO	3
OUTROS ________ ESPECIFIQUE	4
NS	8
NR	9

D.27_7d

Quanto o(a) Sr(a) se sente limitado para executar essa atividade

NADA	1
UM POUCO/ ÀS VEZES	2
MUITO / TOTALMENTE	3
NS	8
NR	9

**D.27_8a**<br>Viaja para fora da cidade pernoitando pelo menos uma noite?	SIM .... 1<br>NÃO .... 2 → **VÁ p/ 27_8c**<br>NS .... 8 } **VÁ p/ 27_9a**<br>NR 9
**D.27_8b**<br>Gostaria de saber qual o principal motivo pelo qual o(s) Sr(a) **FAZ** essa atividade	LAZER .... 1<br>TRABALHO .... 2<br>CONTATO SOCIAL .... 3<br>ATIVIDADE FÍSICA/EXERCÍCIO .... 4<br>OUTROS ________ 5<br>ESPECIFIQUE<br>NS .... 8<br>NR .... 9<br>} **Vá para 27_8d**
**D.27_8c**<br>Gostaria de saber qual o motivo pelo qual o(s) Sr(a) **NÃO FAZ** essa atividade	NÃO TENHO INTERESSE NELA/NÃO GOSTO .... 1<br>LIMITAÇÃO FUNCIONAL .... 2<br>NECESSITO DE AJUDA PARA EXECUTÁ-LA, MAS NÃO A TENHO ... 3<br>OUTROS ________ 4<br>ESPECIFIQUE<br>NS .... 8<br>NR 9
**D.27_8d**<br>Quanto o(a) Sr(a) se sente limitado para executar essa atividade	NADA .... 1<br>UM POUCO/ ÀS VEZES .... 2<br>MUITO / TOTALMENTE .... 3<br>NS .... 8<br>NR 9
**D.27_9a**<br>Participa de algum programa de exercícios regulares, tais como esportes, exercícios físicos, caminhadas e grupos de práticas corporais?	SIM .... 1<br>NÃO .... 2 → **VÁ p/ 27_9c**<br>NS .... 8 } **VÁ p/ 27_10a**<br>NR 9
**D.27_9b**<br>Gostaria de saber qual o principal motivo pelo qual o(s) Sr(a) **FAZ** essa atividade	LAZER .... 1<br>TRABALHO .... 2<br>CONTATO SOCIAL .... 3<br>ATIVIDADE FÍSICA/EXERCÍCIO .... 4<br>OUTROS ________ 5<br>ESPECIFIQUE<br>NS .... 8<br>NR .... 9<br>} **Vá para 27_9d**

D.27_9c

Gostaria de saber qual o motivo pelo qual o(s) Sr(a) **NÃO FAZ** essa atividade

NÃO TENHO INTERESSE NELA/NÃO GOSTO	1
LIMITAÇÃO FUNCIONAL	2
NECESSITO DE AJUDA PARA EXECUTÁ-LA, MAS NÃO A TENHO	3
OUTROS ____________ ESPECIFIQUE	4
NS	8
NR	9

D.27_9d

Quanto o(a) Sr(a) se sente limitado para executar essa atividade

NADA	1
UM POUCO/ ÀS VEZES	2
MUITO / TOTALMENTE	3
NS	8
NR	9

D.27_10a

Convida pessoas para virem à sua casa para refeições ou lazer?

SIM	1	
NÃO	2	VÁ p/ 27_10c
NS	8	VÁ p/ 27_11a
NR	9	VÁ p/ 27_11a

D.27_10b

Gostaria de saber qual o principal motivo pelo qual o(s) Sr(a) **FAZ** essa atividade

LAZER	1	Vá para 27_10d
TRABALHO	2	
CONTATO SOCIAL	3	
ATIVIDADE FÍSICA/EXERCÍCIO	4	
OUTROS ____________ ESPECIFIQUE	5	
NS	8	
NR	9	

D.27_10c

Gostaria de saber qual o motivo pelo qual o(s) Sr(a) **NÃO FAZ** essa atividade

NÃO TENHO INTERESSE NELA/NÃO GOSTO	1
LIMITAÇÃO FUNCIONAL	2
NECESSITO DE AJUDA PARA EXECUTÁ-LA, MAS NÃO A TENHO	3
OUTROS ____________ ESPECIFIQUE	4
NS	8
NR	9

D.27_10d

Quanto o(a) Sr(a) se sente limitado para executar essa atividade

NADA	1
UM POUCO/ ÀS VEZES	2
MUITO / TOTALMENTE	3
NS	8
NR	9

D.27_11a

Sai com outras pessoas para lugares públicos como restaurante ou cinema?

SIM	1	
NÃO	2	VÁ p/ 27_11c
NS	8	VÁ p/ 27_12a
NR	9	VÁ p/ 27_12a

D.27_11b

Gostaria de saber qual o principal motivo pelo qual o(s) Sr(a) **FAZ** essa atividade

LAZER	1	Vá para 27_11d
TRABALHO	2	
CONTATO SOCIAL	3	
ATIVIDADE FÍSICA/EXERCÍCIO	4	
OUTROS ______ ESPECIFIQUE	5	
NS	8	
NR	9	

D.27_11c

Gostaria de saber qual o motivo pelo qual o(s) Sr(a) **NÃO FAZ** essa atividade

NÃO TENHO INTERESSE NELA/NÃO GOSTO	1
LIMITAÇÃO FUNCIONAL	2
NECESSITO DE AJUDA PARA EXECUTÁ-LA, MAS NÃO A TENHO	3
OUTROS ______ ESPECIFIQUE	4
NS	8
NR	9

D.27_11d

Quanto o(a) Sr(a) se sente limitado para executar essa atividade

NADA	1
UM POUCO/ ÀS VEZES	2
MUITO / TOTALMENTE	3
NS	8
NR	9

D.27_12a

Cuida de suas necessidades pessoais (banho, vestimenta e toalete)?

SIM	1	
NÃO	2	VÁ p/ 27_12c
NS	8	VÁ p/ 27_13a
NR	9	VÁ p/ 27_13a

D.27_12b

Gostaria de saber qual o principal motivo pelo qual o(s) Sr(a) **FAZ** essa atividade

LAZER	1	Vá para 27_12d
TRABALHO	2	
CONTATO SOCIAL	3	
ATIVIDADE FÍSICA/EXERCÍCIO	4	
OUTROS ______ ESPECIFIQUE	5	
NS	8	
NR	9	

D.27_12c

Gostaria de saber qual o motivo pelo qual o(s) Sr(a) **NÃO FAZ** essa atividade

NÃO TENHO INTERESSE NELA/NÃO GOSTO	1
LIMITAÇÃO FUNCIONAL	2
NECESSITO DE AJUDA PARA EXECUTÁ-LA, MAS NÃO A TENHO	3
OUTROS ________ (ESPECIFIQUE)	4
NS	8
NR	9

D.27_12d

Quanto o(a) Sr(a) se sente limitado para executar essa atividade

NADA	1
UM POUCO/ ÀS VEZES	2
MUITO / TOTALMENTE	3
NS	8
NR	9

D.27_13a

Participa de atividades sociais organizadas (clubes, grupos comunitários ou religiosos, centros de convivência de idosos, bingo, universidade da 3ª idade)?

SIM	1	
NÃO	2 →	VÁ p/ 27_13c
NS	8	VÁ p/ 27_14a
NR	9	VÁ p/ 27_14a

D.27_13b

Gostaria de saber qual o principal motivo pelo qual o(s) Sr(a) **FAZ** essa atividade

LAZER	1	Vá para 27_13d
TRABALHO	2	
CONTATO SOCIAL	3	
ATIVIDADE FÍSICA/EXERCÍCIO	4	
OUTROS ________ (ESPECIFIQUE)	5	
NS	8	
NR	9	

D.27_13c

Gostaria de saber qual o motivo pelo qual o(s) Sr(a) **NÃO FAZ** essa atividade

NÃO TENHO INTERESSE NELA/NÃO GOSTO	1
LIMITAÇÃO FUNCIONAL	2
NECESSITO DE AJUDA PARA EXECUTÁ-LA, MAS NÃO A TENHO	3
OUTROS ________ (ESPECIFIQUE)	4
NS	8
NR	9

D.27_13d

Quanto o(a) Sr(a) se sente limitado para executar essa atividade

NADA	1
UM POUCO/ ÀS VEZES	2
MUITO / TOTALMENTE	3
NS	8
NR	9

D.27_14a

Cuida de suas responsabilidades pessoais (compras em geral, compras de alimentos ir ao banco, lavanderia, etc)?

SIM	1	
NÃO	2 →	VÁ p/ 27_14c
NS	8	VÁ p/ 27_15a
NR	9	

D.27_14b

Gostaria de saber qual o principal motivo pelo qual o(s) Sr(a) **FAZ** essa atividade

LAZER	1	Vá para 27_14d
TRABALHO	2	
CONTATO SOCIAL	3	
ATIVIDADE FÍSICA/EXERCÍCIO	4	
OUTROS ______ ESPECIFIQUE	5	
NS	8	
NR	9	

D.27_14c

Gostaria de saber qual o motivo pelo qual o(s) Sr(a) **NÃO FAZ** essa atividade

NÃO TENHO INTERESSE NELA/NÃO GOSTO	1
LIMITAÇÃO FUNCIONAL	2
NECESSITO DE AJUDA PARA EXECUTÁ-LA, MAS NÃO A TENHO	3
OUTROS ______ ESPECIFIQUE	4
NS	8
NR	9

D.27_14d

Quanto o(a) Sr(a) se sente limitado para executar essa atividade

NADA	1
UM POUCO/ ÀS VEZES	2
MUITO / TOTALMENTE	3
NS	8
NR	9

D.27_15a

Prepara as próprias refeições (incluindo planejamento, preparação, servir e limpeza posterior)?

SIM	1	
NÃO	2 →	VÁ p/ 27_15c
NS	8	VÁ p/ 27_16a
NR	9	

D.27_15b

Gostaria de saber qual o principal motivo pelo qual o(s) Sr(a) **FAZ** essa atividade

LAZER	1	Vá para 27_15d
TRABALHO	2	
CONTATO SOCIAL	3	
ATIVIDADE FÍSICA/EXERCÍCIO	4	
OUTROS ______ ESPECIFIQUE	5	
NS	8	
NR	9	

D.27_15c

Gostaria de saber qual o motivo pelo qual o(s) Sr(a) **<u>NÃO FAZ</u>** essa atividade

NÃO TENHO INTERESSE NELA/NÃO GOSTO	1
LIMITAÇÃO FUNCIONAL	2
NECESSITO DE AJUDA PARA EXECUTÁ-LA, MAS NÃO A TENHO	3
OUTROS ______ ESPECIFIQUE	4
NS	8
NR	9

D.27_15d

Quanto o(a) Sr(a) se sente limitado para executar essa atividade?

NADA	1
UM POUCO/ ÀS VEZES	2
MUITO / TOTALMENTE	3
NS	8
NR	9

D.27_16a

Faz uso de computador incluindo a Internet?

SIM	1	
NÃO	2	→ **VÁ p/ 27_16c**
NS	8	**VÁ p/ 27_17a**
NR	9	**VÁ p/ 27_17a**

D.27_16b

Gostaria de saber qual o principal motivo pelo qual o(s) Sr(a) **<u>FAZ</u>** essa atividade

LAZER	1	**Vá para 27_16d**
TRABALHO	2	
CONTATO SOCIAL	3	
ATIVIDADE FÍSICA/EXERCÍCIO	4	
OUTROS ______ ESPECIFIQUE	5	
NS	8	
NR	9	

D.27_16c

Gostaria de saber qual o motivo pelo qual o(s) Sr(a) **<u>NÃO FAZ</u>** essa atividade

NÃO TENHO INTERESSE NELA/NÃO GOSTO	1
LIMITAÇÃO FUNCIONAL	2
NECESSITO DE AJUDA PARA EXECUTÁ-LA, MAS NÃO A TENHO	3
OUTROS ______ ESPECIFIQUE	4
NS	8
NR	9

D.27_16d

Quanto o(a) Sr(a) se sente limitado para executar essa atividade?

NADA	1
UM POUCO/ ÀS VEZES	2
MUITO / TOTALMENTE	3
NS	8
NR	9

**D.27_17a** Dirige veículos a motor?	
	SIM 1
	NÃO .... 2 → **VÁ p/ 27_17c**
	NS .... 8 ⎤ **VÁ p/ 27_18a**
	NR .... 9 ⎦

**D.27_17b** Gostaria de saber qual o principal motivo pelo qual o(s) Sr(a) **FAZ** essa atividade	
	LAZER 1
	TRABALHO 2
	CONTATO SOCIAL 3
	ATIVIDADE FÍSICA/EXERCÍCIO 4
	OUTROS ____________ ESPECIFIQUE 5
	NS 8
	NR 9
	(1–9) **Vá para 27_17d**

**D.27_17c** Gostaria de saber qual o motivo pelo qual o(s) Sr(a) **NÃO FAZ** essa atividade	
	NÃO TENHO INTERESSE NELA/NÃO GOSTO 1
	LIMITAÇÃO FUNCIONAL 2
	NECESSITO DE AJUDA PARA EXECUTÁ-LA, MAS NÃO A TENHO ... 3
	OUTROS ____________ ESPECIFIQUE 4
	NS 8
	NR 9

**D.27_17d** Quanto o(a) Sr(a) se sente limitado para executar essa atividade	
	NADA 1
	UM POUCO/ ÀS VEZES 2
	MUITO / TOTALMENTE 3
	NS 8
	NR 9

**D.27_18a** Faz trabalhos manuais como pintura, escultura, desenho, bordado, tricô, crochê, marcenaria, jardinagem, cuidar da horta, etc?	
	SIM 1
	NÃO .... 2 → **VÁ p/ 27_18c**
	NS .... 8 ⎤ **VÁ p/ 27_19a**
	NR .... 9 ⎦

**D.27_18b** Gostaria de saber qual o principal motivo pelo qual o(s) Sr(a) **FAZ** essa atividade	
	LAZER 1
	TRABALHO 2
	CONTATO SOCIAL 3
	ATIVIDADE FÍSICA/EXERCÍCIO 4
	OUTROS ____________ ESPECIFIQUE 5
	NS 8
	NR 9
	(1–9) **Vá para 27_18d**

D.27_18c

Gostaria de saber qual o motivo pelo qual o(s) Sr(a) **NÃO FAZ** essa atividade

NÃO TENHO INTERESSE NELA/NÃO GOSTO	1
LIMITAÇÃO FUNCIONAL	2
NECESSITO DE AJUDA PARA EXECUTÁ-LA, MAS NÃO A TENHO	3
OUTROS ________ ESPECIFIQUE	4
NS	8
NR	9

D.27_18d

Quanto o(a) Sr(a) se sente limitado para executar essa atividade?

NADA	1
UM POUCO/ ÀS VEZES	2
MUITO / TOTALMENTE	3
NS	8
NR	9

D.27_19a

Envolve-se em jogos de mesa, tais como jogo de damas, dominó, baralho, etc?

SIM	1	
NÃO	2	→ VÁ p/ 27_19c
NS	8	VÁ p/ 27_20a
NR	9	VÁ p/ 27_20a

D.27_19b

Gostaria de saber qual o principal motivo pelo qual o(s) Sr(a) **FAZ** essa atividade

LAZER	1	Vá para 27_19d
TRABALHO	2	Vá para 27_19d
CONTATO SOCIAL	3	Vá para 27_19d
ATIVIDADE FÍSICA/EXERCÍCIO	4	Vá para 27_19d
OUTROS ________ ESPECIFIQUE	5	Vá para 27_19d
NS	8	Vá para 27_19d
NR	9	Vá para 27_19d

D.27_19c

Gostaria de saber qual o motivo pelo qual o(s) Sr(a) **NÃO FAZ** essa atividade

NÃO TENHO INTERESSE NELA/NÃO GOSTO	1
LIMITAÇÃO FUNCIONAL	2
NECESSITO DE AJUDA PARA EXECUTÁ-LA, MAS NÃO A TENHO	3
OUTROS ________ ESPECIFIQUE	4
NS	8
NR	9

D.27_19d

Quanto o(a) Sr(a) se sente limitado para executar essa atividade?

NADA	1
UM POUCO/ ÀS VEZES	2
MUITO / TOTALMENTE	3
NS	8
NR	9

D.27_20a

Participa de atividades de relaxamento: assistir televisão, ouvir rádio ou músicas?

SIM	1	
NÃO	2 →	VÁ p/ 27_20c
NS	8	VÁ p/ 27_21a
NR	9	

D.27_20b

Gostaria de saber qual o principal motivo pelo qual o(s) Sr(a) **FAZ** essa atividade

LAZER	1	Vá para 27_20d
TRABALHO	2	
CONTATO SOCIAL	3	
ATIVIDADE FÍSICA/EXERCÍCIO	4	
OUTROS ____________ ESPECIFIQUE	5	
NS	8	
NR	9	

D.27_20c

Gostaria de saber qual o motivo pelo qual o(s) Sr(a) **NÃO FAZ** essa atividade

NÃO TENHO INTERESSE NELA/NÃO GOSTO	1
LIMITAÇÃO FUNCIONAL	2
NECESSITO DE AJUDA PARA EXECUTÁ-LA, MAS NÃO A TENHO	3
OUTROS ____________ ESPECIFIQUE	4
NS	8
NR	9

D.27_20d

Quanto o(a) Sr(a) se sente limitado para executar essa atividade

NADA	1
UM POUCO/ ÀS VEZES	2
MUITO / TOTALMENTE	3
NS	8
NR	9

D.27_21a

Realiza atividades musicais: canta, toca instrumento musical, dança.

SIM	1	
NÃO	2 →	VÁ p/ 27_21c
NS	8	VÁ p/ 27_22a
NR	9	

D.27_21b

Gostaria de saber qual o principal motivo pelo qual o(s) Sr(a) **FAZ** essa atividade

LAZER	1	Vá para 27_21d
TRABALHO	2	
CONTATO SOCIAL	3	
ATIVIDADE FÍSICA/EXERCÍCIO	4	
OUTROS ____________ ESPECIFIQUE	5	
NS	8	
NR	9	

D.27_21c

Gostaria de saber qual o motivo pelo qual o(s) Sr(a) **NÃO FAZ** essa atividade

NÃO TENHO INTERESSE NELA/NÃO GOSTO	1
LIMITAÇÃO FUNCIONAL	2
NECESSITO DE AJUDA PARA EXECUTÁ-LA, MAS NÃO A TENHO	3
OUTROS ______ ESPECIFIQUE	4
NS	8
NR	9

D.27_21d

Quanto o(a) Sr(a) se sente limitado para executar essa atividade?

NADA	1
UM POUCO/ ÀS VEZES	2
MUITO / TOTALMENTE	3
NS	8
NR	9

D.27_22a

Realiza atividades intelectuais: lê jornais e revistas, livros, resolve palavras cruzadas, joga xadrez, monta quebra-cabeças, etc?

SIM	1	
NÃO	2	VÁ p/ 27_22c
NS	8	VÁ p/ 27_23a
NR	9	VÁ p/ 27_23a

D.27_22b

Gostaria de saber qual o principal motivo pelo qual o(s) Sr(a) **FAZ** essa atividade

LAZER	1	Vá para 27_22d
TRABALHO	2	
CONTATO SOCIAL	3	
ATIVIDADE FÍSICA/EXERCÍCIO	4	
OUTROS ______ ESPECIFIQUE	5	
NS	8	
NR	9	

D.27_22c

Gostaria de saber qual o motivo pelo qual o(s) Sr(a) **NÃO FAZ** essa atividade

NÃO TENHO INTERESSE NELA/NÃO GOSTO	1
LIMITAÇÃO FUNCIONAL	2
NECESSITO DE AJUDA PARA EXECUTÁ-LA, MAS NÃO A TENHO	3
OUTROS ______ ESPECIFIQUE	4
NS	8
NR	9

D.27_22d

Quanto o(a) Sr(a) se sente limitado para executar essa atividade?

NADA	1
UM POUCO/ ÀS VEZES	2
MUITO / TOTALMENTE	3
NS	8
NR	9

D.27_23a

Realiza atividades culturais: vai ao cinema, teatro, shows, festas, bingos, museus, concertos, eventos esportivos?

SIM	1	
NÃO	2 →	VÁ p/ 27_23c
NS	8	VÁ p/ 27_24a
NR	9	

D.27_23b

Gostaria de saber qual o principal motivo pelo qual o(s) Sr(a) **FAZ** essa atividade

LAZER	1	Vá para 27_23d
TRABALHO	2	
CONTATO SOCIAL	3	
ATIVIDADE FÍSICA/EXERCÍCIO	4	
OUTROS ____________ ESPECIFIQUE	5	
NS	8	
NR	9	

D.27_23c

Gostaria de saber qual o motivo pelo qual o(s) Sr(a) **NÃO FAZ** essa atividade

NÃO TENHO INTERESSE NELA/NÃO GOSTO	1
LIMITAÇÃO FUNCIONAL	2
NECESSITO DE AJUDA PARA EXECUTÁ-LA, MAS NÃO A TENHO	3
OUTROS ____________ ESPECIFIQUE	4
NS	8
NR	9

D.27_23d

Quanto o(a) Sr(a) se sente limitado para executar essa atividade

NADA	1
UM POUCO/ ÀS VEZES	2
MUITO / TOTALMENTE	3
NS	8
NR	9

D.27_24a

Participa de atividades políticas: em partidos políticos, associações civis, lideranças comunitárias, cooperativas?

SIM	1	
NÃO	2 →	VÁ p/ 27_24c
NS	8	VÁ p/ 27_25a
NR	9	

D.27_24b

Gostaria de saber qual o principal motivo pelo qual o(s) Sr(a) **FAZ** essa atividade

LAZER	1	Vá para 27_24d
TRABALHO	2	
CONTATO SOCIAL	3	
ATIVIDADE FÍSICA/EXERCÍCIO	4	
OUTROS ____________ ESPECIFIQUE	5	
NS	8	
NR	9	

D.27_24c

Gostaria de saber qual o motivo pelo qual o(s) Sr(a) **NÃO FAZ** essa atividade

NÃO TENHO INTERESSE NELA/NÃO GOSTO	1
LIMITAÇÃO FUNCIONAL	2
NECESSITO DE AJUDA PARA EXECUTÁ-LA, MAS NÃO A TENHO	3
OUTROS ______ ESPECIFIQUE	4
NS	8
NR	9

D.27_24d

Quanto o(a) Sr(a) se sente limitado para executar essa atividade?

NADA	1
UM POUCO/ ÀS VEZES	2
MUITO / TOTALMENTE	3
NS	8
NR	9

D.27_25a

Realiza trabalho remunerado?

SIM	1	
NÃO	2	VÁ p/ 27_25c
NS	8	VÁ p/ 27_26a
NR	9	VÁ p/ 27_26a

D.27_25b

Gostaria de saber qual o principal motivo pelo qual o(s) Sr(a) **FAZ** essa atividade

LAZER	1	Vá para 27_25d
TRABALHO	2	Vá para 27_25d
CONTATO SOCIAL	3	Vá para 27_25d
ATIVIDADE FÍSICA/EXERCÍCIO	4	Vá para 27_25d
OUTROS ______ ESPECIFIQUE	5	Vá para 27_25d
NS	8	Vá para 27_25d
NR	9	Vá para 27_25d

D.27_25c

Gostaria de saber qual o motivo pelo qual o(s) Sr(a) **NÃO FAZ** essa atividade

NÃO TENHO INTERESSE NELA/NÃO GOSTO	1
LIMITAÇÃO FUNCIONAL	2
NECESSITO DE AJUDA PARA EXECUTÁ-LA, MAS NÃO A TENHO	3
OUTROS ______ ESPECIFIQUE	4
NS	8
NR	9

D.27_25d

Quanto o(a) Sr(a) se sente limitado para executar essa atividade?

NADA	1
UM POUCO/ ÀS VEZES	2
MUITO / TOTALMENTE	3
NS	8
NR	9

**D.27_26a**<br>Cuida de animais domésticos?	SIM .... 1<br>NÃO .... 2 → **VÁ p/ 27_26c**<br>NS .... 8<br>NR .... 9<br>(8, 9) → **VÁ p/ 27_27a**
**D.27_26b**<br>Gostaria de saber qual o principal motivo pelo qual o(s) Sr(a) **FAZ** essa atividade	LAZER .... 1<br>TRABALHO .... 2<br>CONTATO SOCIAL .... 3<br>ATIVIDADE FÍSICA/EXERCÍCIO .... 4<br>OUTROS ______ (ESPECIFIQUE) 5<br>NS .... 8<br>NR .... 9<br>(1–9) → **Vá para 27_26d**
**D.27_26c**<br>Gostaria de saber qual o motivo pelo qual o(s) Sr(a) **NÃO FAZ** essa atividade	NÃO TENHO INTERESSE NELA/NÃO GOSTO .... 1<br>LIMITAÇÃO FUNCIONAL .... 2<br>NECESSITO DE AJUDA PARA EXECUTÁ-LA, MAS NÃO A TENHO ... 3<br>OUTROS ______ (ESPECIFIQUE) 4<br>NS .... 8<br>NR 9
**D.27_26d**<br>Quanto o(a) Sr(a) se sente limitado para executar essa atividade	NADA .... 1<br>UM POUCO/ ÀS VEZES .... 2<br>MUITO / TOTALMENTE .... 3<br>NS .... 8<br>NR 9
**D.27_27a**<br>Reza ou ora em casa?	SIM .... 1<br>NÃO .... 2 → **VÁ p/ 27_27c**<br>NS .... 8<br>NR .... 9<br>(8, 9) → **VÁ p/ 27_28a**
**D.27_27b**<br>Gostaria de saber qual o principal motivo pelo qual o(s) Sr(a) **FAZ** essa atividade	LAZER .... 1<br>TRABALHO .... 2<br>CONTATO SOCIAL .... 3<br>ATIVIDADE FÍSICA/EXERCÍCIO .... 4<br>OUTROS ______ (ESPECIFIQUE) 5<br>NS .... 8<br>NR .... 9<br>(1–9) → **Vá para 27_27d**

D.27_27c

Gostaria de saber qual o motivo pelo qual o(s) Sr(a) **NÃO FAZ** essa atividade

NÃO TENHO INTERESSE NELA/NÃO GOSTO	1
LIMITAÇÃO FUNCIONAL	2
NECESSITO DE AJUDA PARA EXECUTÁ-LA, MAS NÃO A TENHO	3
OUTROS ________ ESPECIFIQUE	4
NS	8
NR	9

D.27_27d

Quanto o(a) Sr(a) se sente limitado para executar essa atividade?

NADA	1
UM POUCO/ ÀS VEZES	2
MUITO / TOTALMENTE	3
NS	8
NR	9

D.27_28a

Participa de bate papos na internet ou utiliza redes sociais (facebook, orkut, etc)?

SIM	1	
NÃO	2	→ VÁ p/ 27_28c
NS	8	VÁ p/ E1
NR	9	VÁ p/ E1

D.27_28b

Gostaria de saber qual o principal motivo pelo qual o(s) Sr(a) **FAZ** essa atividade

LAZER	1	Vá para 27_28d
TRABALHO	2	Vá para 27_28d
CONTATO SOCIAL	3	Vá para 27_28d
ATIVIDADE FÍSICA/EXERCÍCIO	4	Vá para 27_28d
OUTROS ________ ESPECIFIQUE	5	Vá para 27_28d
NS	8	Vá para 27_28d
NR	9	Vá para 27_28d

D.27_28c

Gostaria de saber qual o motivo pelo qual o(s) Sr(a) **NÃO FAZ** essa atividade

NÃO TENHO INTERESSE NELA/NÃO GOSTO	1
LIMITAÇÃO FUNCIONAL	2
NECESSITO DE AJUDA PARA EXECUTÁ-LA, MAS NÃO A TENHO	3
OUTROS ________ ESPECIFIQUE	4
NS	8
NR	9

D.27_28d

Quanto o(a) Sr(a) se sente limitado para executar essa atividade?

NADA	1
UM POUCO/ ÀS VEZES	2
MUITO / TOTALMENTE	3
NS	8
NR	9

SEÇÃO E- MEDICAMENTOS

E. 1<br>O(a) Sr(a) poderia me mostrar os medicamentos (remédios) que está tomando (usando) atualmente?	Sim	1
	Não	2
	O(a) idoso(a) **<u>NÃO TOMA</u>** medicamentos	3 Vá para E.6

E. 2 **LISTA DE MEDICAMENTOS**	E. 3 **QUEM O RECEITOU?**	E. 5a **Como obteve o medicamento(remédio)?**
O(A) Sr(a) poderia me dizer o nome dos medicamentos (remédios) que está tomando (usando)?<br>Coloque o nome comercial e o nome genérico.<br><br>Anote todos os medicamentos (remédios) mostrados ou referidos.	Médico(a)......................... 1<br>Farmacêutico(a)............... 2<br>Enfermeiro(a).................. 3<br>O(a)próprio (a) idoso(a).... 4<br>Outra pessoa.................... 5<br>NS.................................... 8<br>NR.................................... 9	O(a) idoso ou outra pessoa pagou por ele......................... 1<br>O(a) idoso ou outra pessoa obteve gratuitamente............ 2<br>NS.. 8<br>NR.. 9

	**Medicamento (remédio)**	**Responsabilidade**	**Obtenção**
1	(nome comercial)<br>(nome genérico)<br>|___|___|___|___|___|___|___|___|___|___|	|_____|	|_____|
2	(nome comercial)<br>(nome genérico)<br>|___|___|___|___|___|___|___|___|___|___|	|_____|	|_____|
3	(nome comercial)<br>(nome genérico)<br>|___|___|___|___|___|___|___|___|___|___|	|_____|	|_____|
4	(nome comercial)<br>(nome genérico)<br>|___|___|___|___|___|___|___|___|___|___|	|_____|	|_____|
5	(nome comercial)<br>(nome genérico)<br>|___|___|___|___|___|___|___|___|___|___|	|_____|	|_____|

SEÇÃO E- MEDICAMENTOS

6	 (nome comercial)<br><br>(nome genérico)<br>|___|___|___|___|___|___|___|___|___|___|	|______|	|______|
7	 (nome comercial)<br><br>(nome genérico)<br>|___|___|___|___|___|___|___|___|___|___|	|______|	|______|
8	 (nome comercial)<br><br>(nome genérico)<br>|___|___|___|___|___|___|___|___|___|___|	|______|	|______|
9	 (nome comercial)<br><br>(nome genérico)<br>|___|___|___|___|___|___|___|___|___|___|	|______|	|______|
10	 (nome comercial)<br><br>(nome genérico)<br>|___|___|___|___|___|___|___|___|___|___|	|______|	|______|
11	 (nome comercial)<br><br>(nome genérico)<br>|___|___|___|___|___|___|___|___|___|___|	|______|	|______|
12	 (nome comercial)<br><br>(nome genérico)<br>|___|___|___|___|___|___|___|___|___|___|	|______|	|______|

SEÇÃO E- MEDICAMENTOS

13	(nome comercial)<br>(nome genérico)<br>|___|___|___|___|___|___|___|___|___|___|	|______|	|______|
14	(nome comercial)<br>(nome genérico)<br>|___|___|___|___|___|___|___|___|___|___|	|______|	|______|
15	(nome comercial)<br>(nome genérico)<br>|___|___|___|___|___|___|___|___|___|___|	|______|	|______|
16	(nome comercial)<br>(nome genérico)<br>|___|___|___|___|___|___|___|___|___|___|	|______|	|______|
17	(nome comercial)<br>(nome genérico)<br>|___|___|___|___|___|___|___|___|___|___|	|______|	|______|
18	(nome comercial)<br>(nome genérico)<br>|___|___|___|___|___|___|___|___|___|___|	|______|	|______|

Observações:

____
____
____
____
____

SEÇÃO E- MEDICAMENTOS

Pergunta	Resposta
E. 5b<br>De modo geral, como o(a) Sr(a) identifica seus medicamentos (remédios)?	Pela COR........ 1<br>Pelo TAMANHO .... 2<br>Pela FORMA .... 3<br>Pelo NOME .... 4<br>Pela EMBALAGEM .... 5<br>De outra forma........ 6<br>(1 a 6: Vá para E6)<br>..<br>(especifique)<br>Não identifica........ 7<br>NS .... 8<br>NR 9
E. 5c<br>Como o(a) Sr(a) disse que tem dificuldade para identificar seu(s) medicamento(s), diga-me como faz para tomá-los corretamente?	Alguém fornece........ 1<br>Alguém deixa separado DIARIAMENTE .... 2<br>Alguém deixa separado SEMANALMENTE .... 3<br>De outra forma........ 7<br>..<br>(especifique) 8<br>NS .... 9<br>NR ....
E. 6<br>Atualmente, para cuidar de sua saúde, o(a) Sr(a) faz uso de CHÁS, ERVAS, PRODUTOS HOMEOPÁTICOS OU FITOTERÁPICOS?	Sim .... 1 Volte para E2 e anote<br>Não .... 2<br>NS .... 8<br>NR 9
E. 7a<br>Atualmente, para cuidar de sua saúde, o(a) Sr(a) faz uso VITAMINAS (em comprimidos) ou SUPLEMENTOS ALIMENTARES?	Sim .... 1 Volte para E2 e anote<br>Não .... 2<br>NS .... 8<br>NR 9
E. 7b<br>Atualmente, para cuidar de sua saúde, além do que o(a) Sr(a) já me falou, faz uso de laxantes, medicamentos para gripe, medicamentos para dormir?	Sim .... 1 Volte para E2 e anote<br>Não .... 2<br>NS .... 8<br>NR 9
E. 7c FILTRO<br>Número de medicamentos (remédios) utilizados pelo(a) idoso(a):	Não toma NENHUM........ 02 Vá para E.9e<br>Toma APENAS UM .... 01<br>Toma MAIS QUE UM........ 06<br>Quantos medicamentos diferentes utiliza por dia? .... |___|___| NS .... 98<br>NR........ 99
E. 8<br>Durante o último mês, no total, quanto o(a) Sr(a) gastou com medicamentos (remédios) incluindo seringas, cremes, pomadas, etc; comprados com ou sem receita médica?	Nada........ 0<br>Gasto total........ R$|_|_|_|_|,|_|_| NS .... 8<br>NR........ 9

SEÇÃO E- MEDICAMENTOS

E. 9a<br>Em algum momento, nos últimos 12 meses (último ano), o(a) Sr(a) deixou de tomar algum dos medicamentos (remédios) prescritos?	Sim .... 1<br>Não .... 2<br>NS .... 8<br>NR .... 9<br>(2, 8, 9) Vá para E9c
E. 9b<br>Por que?	Não tinha dinheiro para comprar........ 1<br>O medicamento (remédio) não estava disponível .... 2<br>Não se sentiu bem quando tomou .... 3<br>Não gosta de tomar medicamentos (remédios) .... 4<br>Outro motivo .... 5<br>........<br>(especifique)<br>NS .... 8<br>NR 9
E. 9c<br>Em algum momento, nos últimos 12 meses (último ano), o(a) Sr(a) tomou uma quantidade de medicamentos (remédios) **<u>MENOR</u>** do que estava prescrito?	Sim .... 1<br>Não .... 2<br>NS .... 8<br>NR .... 9<br>(2, 8, 9) Vá para E9e
E. 9d<br>Por que?	Não tinha dinheiro para comprar........ 1<br>O medicamento (remédio) não estava disponível .... 2<br>Não se sentiu bem quando tomou .... 3<br>Não achei necessário tomar a quantidade (dose) prescrita .. 4<br>Outro motivo .... 5<br>........<br>(especifique)<br>NS .... 8<br>NR 9
E. 9e<br>Em algum momento, nos últimos 12 meses (último ano), o(a) Sr(a) **<u>PAROU DE TOMAR</u>** algum medicamento (remédio) porque **NÃO SE SENTIU BEM** quando tomou?	Sim .... 1<br>Não .... 2<br>NS .... 8<br>NR .... 9<br>(2, 8, 9) Vá para Seção F
E. 9f<br>O(a) Sr(a) poderia me dizer o nome desse (s) medicamento(s) (remédio(s))?	Sim .... 1<br>........<br>........<br>........<br>........<br>........<br>........<br>........<br>........<br>........<br>(especifique)<br>Não .... 2<br>Não lembra .... 8<br>NR 9

SEÇÃO F : USO E ACESSO A SERVIÇOS

Pergunta	Respostas
F.1b<br>O(a) Sr(a) tem algum plano de saúde (convênio) além do Sistema único de Saúde:<br>**01**	SIM.. **1**<br>NÃO.. **2**<br>NS .... **8**<br>NR .... **9**<br>(2, 8, 9) Vá para **05**
F.1c<br>Que tipo de plano de saúde o(a) Sr(a) tem?<br>ASSINALE TODAS AS RESPOSTAS MENCIONADAS<br>**02**	Plano de Assistência ao Servidor Público .. **2**<br>Plano de Saúde / Convênio Particular ...... **6**<br>Plano de Saúde / Convênio Empresa .... **7**<br>Outro .... **8**<br>NS .... **98**<br>NR .... **99**<br><br>Especifique o nome do Convênio / Plano de Saúde:<br>____________________________________<br>____________________________________<br>____________________________________
F.1d<br>Quem paga pelo plano/convênio de saúde?<br>**03**	O próprio idoso (sozinho) .... **1**<br>O idoso + outra pessoa .... **7**<br>Outra pessoa .... **8**<br>NS .... **98**<br>NR **99**
F.1e<br>Quanto paga pelo plano/convênio de saúde?<br>**04**	|___|___|___|___|,|___|___| reais<br>NS .... **8**<br>NR **9**
F.3b<br>O(A) Sr(a) tem alguma dificuldade para utilizar ou acessar serviços de saúde, quando precisa?<br>**05**	Sim .... **1**<br>Não .... **2**<br>NS .... **8**<br>NR .... **9**<br>(2, 8, 9) Vá para **07**

SEÇÃO F : USO E ACESSO A SERVIÇOS

F.3c<br>Qual(is) o(s) motivo(s)?<br><br>**06**	Não tenho dinheiro para o transporte .... **1**<br>Não tem companhia para levá-lo(a) .... **3**<br>Dificuldade para se locomover .... **4**<br>O atendimento é ruim .... **5**<br>A distância é muito longa .... **7**<br>Outros: .... **8**<br>*(especifique)*<br>NS .... **98**<br>NR **99**
F.4a<br>Durante os últimos 12 meses, o(a) Sr (a) precisou ser internado em um hospital POR PELO MENOS UMA NOITE?<br><br>Exceto em CASA DE REPOUSO/ ASILO<br>**07**	Sim .... 1<br>Quantas vezes: |___|___| Não .... **2**<br>NS .... **98**<br>NR .... **99**<br>(Não, NS, NR) Vá para **15**
F.5b<br>No total, quantos dias esteve internado nos últimos 12 meses?<br><br>**08**	Número de dias: |___|___| NS .... **98**<br>NR **99**
F.6<br>Onde o(a) Sr(a) esteve internado pela **ÚLTIMA VEZ?**<br><br>**09**	Hospital geral por motivos clínicos .... **7**<br>Hospital geral por motivos cirúrgicos.......... **8**<br>Outros .... **6**<br>(especifique)<br>NS .... **98**<br>NR **99**
F.6a<br>Há quanto tempo foi essa **ÚLTIMA INTERNAÇÃO?**<br><br>**10**	Há menos de 15 dias .... **1**<br>Entre 15 dias e 1 mês .... **2**<br>Entre 1 e 3 meses .... **3**<br>Entre 4 e 6 meses .... **4**<br>Entre 6 meses e 1 ano .... **5**<br>Há mais de 1 ano .... **6**<br>NS .... **8**<br>NR **9**

F.6c<br><br>O (a) Sr (a) poderia me dizer qual o **MOTIVO** dessa última internação?<br><br>**11**	Sim .... **1**<br>____<br>____<br>____<br>____<br>____<br>____<br>(especifique)<br>Prefere não dizer .... **2**<br>NS .... **98**<br>NR **99**
F.6b<br><br>Poderia me dizer o nome e o endereço desse hospital?<br><br>**12**	Nome da Instituição:<br>____<br>____<br>____<br>Endereço:<br>____<br>____<br>____<br><br>NS .... **98**<br>NR **99**
F.9<br>Sua ÚLTIMA INTERNAÇÃO foi feita:<br><br>**13**	Pelo SUS .... **1**<br>Pelo convênio / Plano de Saúde .... **2**<br>Particular .... **3**<br>NS .... **8**<br>NR **9**
F.10a<br>O(a) Sr(a) considera que o atendimento recebido nessa ÚLTIMA INTERNAÇÃO foi:<br><br>**14**	Muito Bom .... **1**<br>Bom .... **2**<br>Regular .... **3**<br>Ruim .... **4**<br>Muito Ruim .... **5**<br>NS .... **8**<br>NR **9**

F.12c<br>Excetuando-se a(s) internação(ões) que perguntei realizou algum atendimento de saúde de **URGÊNCIA / EMERGÊNCIA?**<br>**15**	Sim.. **1**<br>Quantas vezes: |___|___| Não .... **2**<br>NS .... **8**<br>NR .... **9**<br>(2, 8, 9) Vá para **21**
F.12d<br>O(a) Sr(a) poderia me dizer o **MOTIVO** do último atendimento de **URGÊNCIA/EMERGÊNCIA?**<br>**16**	Sim .... **1**<br>______________________________<br>______________________________<br>______________________________<br>______________________________<br>______________________________<br>(especifique)<br>Prefere não dizer .... **2**<br>NS .... **98**<br>NR **99**
F.12f<br>Há quanto tempo foi esse último atendimento de **URGÊNCIA/ EMERGÊNCIA?**<br>**17**	Há menos de 15 dias .... **1**<br>Entre 15 dias e 1 mês .... **2**<br>Entre 1 e 3 meses .... **3**<br>Entre 4 e 6 meses .... **4**<br>Entre 6 meses e 1 ano .... **5**<br>Há mais de 1 ano .... **6**<br>NS .... **8**<br>NR **9**
F.12g<br>Quanto tempo demorou para chegar ao local onde fez o último atendimento desse tipo de **URGÊNCIA / EMERGÊNCIA?**<br>**18**	Minutos: |___|___| Horas: |___|___| NS .... **998**<br>NR **999**
F.12h<br>Quando chegou ao local de atendimento de **URGÊNCIA/ EMERGÊNCIA**, quanto tempo esperou para ser atendido por um profissional de saúde?<br>**19**	Minutos: |___|___| Horas: |___|___| NS .... **998**<br>NR **999**

F.12i<br><br>Esse atendimento de **URGÊNCIA/ EMERGÊNCIA** foi feito:<br><br>**20**	Pelo SUS .... **1**<br>Pelo convênio / Plano de Saúde .... **2**<br>Particular .... **3**<br>NS .... **8**<br>NR **9**
F.13_1<br><br>Além das internações e dos atendimentos de urgência /emergência que perguntei, nos últimos 12 meses, realizou alguma consulta médica?<br><br>**21**	Sim .... **1**<br>Número de vezes: |___|___| Não .... **2**<br>NS .... **8**<br>NR .... **9**<br>(2, 8, 9) Vá para **39**
F.13_2<br>O(a) Sr(a) poderia me dizer o **MOTIVO** de sua última **CONSULTA MÉDICA**?<br><br>**22**	Sim .... **1**<br>____________________<br>____________________<br>____________________<br>____________________<br>____________________<br>(especifique)<br>Prefere não dizer .... **2**<br>NS .... **98**<br>NR **99**
F.13_3<br>Há quanto tempo foi sua última **CONSULTA MÉDICA**?<br><br>**23**	Há menos de 15 dias .... **1**<br>Entre 15 dias até 1 mês .... **2**<br>Entre 1 e 3 meses .... **3**<br>Entre 4 e 6 meses .... **4**<br>Entre 6 meses e 1 ano .... **5**<br>Há mais de 1 ano .... **6**<br>NS .... **8**<br>NR **9**

SEÇÃO F : USO E ACESSO A SERVIÇOS

F.14_1<br>Onde foi sua última **CONSULTA MÉDICA**?<br>**24**	Ambulatório / Clínica de Especialidades .... **1**<br>Consultório Particular .... **2**<br>Unidade Básica de Saúde / Centro de Saúde . **3**<br>Centro de Referência do Idoso .... **4**<br>Outro .... **5**<br>(especifique)<br>NS .... **8**<br>NR **9**
F.13_1<br>Com quem foi sua última **CONSULTA MÉDICA**?<br>**25**	Médico Clínico Geral / Médico de Família ..... **1**<br>Médico especialista (exceto Geriatra) .... **2**<br>_______________________________________<br>(*especifique qual especialista*)<br>Médico Geriatra .... **3**<br>NS .... **8**<br>NR **9**
F.15<br>Quanto tempo esperou entre a solicitação da consulta e o atendimento propriamente dito**?**<br>**26**	Dias: |___|___| Meses: |___|___| NS .... **98**<br>NR **99**
F.18<br>Sua ÚLTIMA CONSULTA MÉDICA foi feita:<br>**27**	Pelo SUS .... **1**<br>Pelo convênio / Plano de Saúde .... **2**<br>Particular .... **3**<br>NS .... **8**<br>NR **9**

SEÇÃO F : USO E ACESSO A SERVIÇOS

Pergunta	Respostas	Código	
F.19<br>No total, quanto pagou por essa **ÚLTIMA CONSULTA?**<br>**28**	Não pagou..	**2**	
	|___|___|___|___|,|___|___| reais		
	NS	**8**	
	NR	**9**	
F.20<br>Durante essa consulta, foram solicitados radiografias, exames de laboratórios ou outros exames?<br>**29**	Sim	1	
	Não	**2**	Vá para **33**
	NS	**8**	
	NR	**9**	
F.20a<br>O(A) Sr(A) fez os exames solicitados?<br>**30**	Sim, fez todos	**1**	Vá para **32**
	Não fez todos, mas estão marcados ...	**2**	
	Não fez ou só fez alguns	**3**	
	NS	**8**	
	NR	**9**	
F.21<br>Por que não fez TODOS os exames solicitados?<br>**31**	Por causa dos custos dos exames	**1**	
	Custo do transporte (viagem)	**2**	
	O(s) exame(s) não estava(m) disponível(eis)	**10**	
	Não conseguiu agendar	**11**	
	Não achou necessário	**12**	
	Não tinha companhia para ir	**14**	
	Outro ....<br>(especifique)	**07**	
	NS	**98**	
	NR	**99**	
F.23<br>Os exames foram feitos:<br>ASSINALE TODAS AS RESPOSTAS MENCIONADAS<br>**32**	Pelo SUS	**1**	
	Pelo convênio / Plano de Saúde..............	**2**	
	Particular	**3**	
	NS	**8**	
	NR	**9**	

SEÇÃO F : USO E ACESSO A SERVIÇOS

Pergunta	Respostas	Código	Instrução
F.25<br>Durante esta consulta, foram prescritos ou receitados medicamentos (remédios)?<br>**33**	Sim	1	
	Não	2	Vá para **39**
	NS	8	Vá para **39**
	NR	9	Vá para **39**

Pergunta	Respostas	Código	Instrução
F.26<br>O(A) Sr(a) conseguiu os medicamentos (remédios) que foram prescritos ou receitados?<br>**34**	Sim	1	Vá para **36**
	Alguns sim, outros não	2	
	Não	3	
	NS	8	Vá para **39**
	NR	9	Vá para **39**

Pergunta	Respostas	Código
F.26a<br>Por que não obteve TODOS os medicamentos (remédios)?<br>**35**	Por causa dos custos dos medicamentos	**01**
	Não estava(m) disponível (eis) no SUS	**04**
	Não tinha quem fosse buscá-lo(s)	**06**
	Não tinha dinheiro para o transporte	**07**
	Não tive tempo para buscá-los	**10**
	Não achou necessário	**11**
	Outro .... (especifique)	**05**
	NS	**98**
	NR	**99**

Pergunta	Respostas	Código	Instrução
F.27<br>Teve que pagar por esse(s) medicamento(s) prescrito(s) ou receitado(s)?<br>**36**	Sim	**1**	
	Não	**2**	**Vá para 39**
	NS	**8**	**Vá para 39**
	NR	**9**	**Vá para 39**

Pergunta	Respostas	Código
F.28<br>Quem pagou?<br>**37**	O(A) PROPRIO IDOSO (SOZINHO)	**1**
	IDOSO + OUTRA PESSOA	**2**
	OUTRA PESSOA	**3**
	NS	**8**
	NR	**9**

SEÇÃO F : USO E ACESSO A SERVIÇOS

F.29<br><br>No total, quanto pagou por esse(s) medicamento(s)?<br><br>**38**	|___|___|___|___|,|___|___| reais<br>NS .... **8**<br>NR **9**
F.33<br>Nos últimos 12 meses, o(a) Sr(a) recebeu algum outro tipo de atendimento em unidade de saúde?<br><br>**39**	Sim .... 1<br>Não .... 2 } Vá para **41**<br>NS .... 8 } Vá para **41**<br>NR .... 9 } Vá para **41**
F.33a<br><br>Que profissional o atendeu?<br><br>**40**	Médico .... **1**<br>Enfermeira / equipe de enfermagem.. **2**<br>Fisioterapeuta .... **3**<br>Dentista .... **4**<br>Agente comunitário .... **5**<br>Outros .... **6**<br>Especifique<br>NS .... **8**<br>NR **9**
F.34<br>Nos últimos 12 meses, o(a) Sr(a) recebeu algum tipo de atendimento de saúde em casa (atendimento domiciliar ou home care)?<br><br>**41**	Sim .... 1<br>Não .... **2** } Vá para **47**<br>NS .... **8** } Vá para **47**<br>NR .... **9** } Vá para **47**
F.34a<br><br>Que profissional o atendeu em sua casa?<br><br>**42**	Médico .... **1**<br>Enfermeira / equipe de enfermagem.. **2**<br>Fisioterapeuta .... **3**<br>Dentista .... **4**<br>Agente comunitário .... **5**<br>Outros .... **6**<br>Especifique<br>NS .... **8**<br>NR **9**

SEÇÃO F : USO E ACESSO A SERVIÇOS

F.34b<br><br>Por que recebeu esse tipo de atendimento de saúde em casa (atendimento domiciliar ou home care)?<br><br>**43**	Estava acamado(a) .... **1**<br>Estava com dificuldade para caminhar **2**<br>Sua situação de saúde havia piorado .. **3**<br>Precisou depois da alta hospitalar .... **4**<br>Outros .... **5**<br>(especifique)<br>NS .... **8**<br>NR **9**

F.34c<br><br>Esse atendimento de saúde em casa (atendimento domiciliar ou home care) que o(a) Sr(a) recebeu foi?<br><br>**44**	Diário .... **1**<br>Uma vez por semana .... **2**<br>2 ou mais vezes por semana .... **3**<br>1 vez a cada 15 dias .... **4**<br>1 vez ao mês .... **5**<br>Outros .... **6**<br>(especifique)<br>NS .... **8**<br>NR **9**

F.34d<br><br>Por quanto tempo recebe(u) esse atendimento de saúde em casa (atendimento domiciliar ou home care)?<br><br>**45**	Menos de 15 dias .... **1**<br>Entre 15 dias e 1 mês .... **2**<br>Entre 1 e 3 meses .... **3**<br>Entre 4 e 6 meses .... **4**<br>Entre 6 meses e 1 ano .... **5**<br>Mais de 1 ano .... **6**<br>NS .... **8**<br>NR **9**

F.34e<br>Quem pagou esse atendimento de saúde em casa (atendimento domiciliar ou home care)?<br><br>**46**	SUS .... **1**<br>O convênio / Plano de Saúde........................ **2**<br>Particular .... **3**<br>NS .... **8**<br>NR **9**

F.32<br><br>AS PERGUNTAS DA SEÇÃO F FORAM **RESPONDIDAS**<br><br>**47**	PELO(A) PRÓPRIO(A) IDOSO (A) .... **1**<br><br>IDOSO(A) + INFORMANTE AUXILIAR .... **2**<br><br>INFORMANTE SUBSTITUTO **3**

DADOS GERAIS DAS PESSOAS QUE RESIDEM NO MESMO DOMICÍLIO DO(A) IDOSO(A)

Anote o nome de todas as pessoas que residem na casa **INCLUINDO O IDOSO** (que deve ser colocado em **primeiro lugar).**

<table>
<tr><th rowspan="2">Número de registro</th><th>G.1</th><th colspan="2">G.4</th><th>G.5</th><th>G.6</th></tr>
<tr><td>Por favor, começando pelo(a) Sr(a), diga-me o nome de cada uma das pessoas que moram nesta casa. ANOTE OS NOMES DE TODOS OS CITADOS COLOCANDO O IDOSO EM PRIMEIRO LUGAR
SE RESIDIR SOZINHO VÁ PARA G18a.</td><td>QUAL É O PARENTESCO DE (NOME) COM O(A) SR(A)?
Esposo(a) / companheiro(a)................ 01
Filho(a).. 02
Pais.. 03
Sogros.. 04
Irmãos.. 05
Genro ou Nora.................................... 06
Neto(a)... 07</td><td>Enteado(a)... 08
Outro familiar não remunerado......... 09
Outro familiar remunerado............... 10
Outro não familiar não remunerado.. 11
Empregado Doméstico...................... 12
Cuidador 13
NS... 98
NR .... 99</td><td>(NOME) É HOMEM OU MULHER?
HOMEM... 1
MULHER... 2</td><td>QUANTOS ANOS COMPLETOS TEM (NOME)?
Anote a idade em anos completos.
Menos de1 ano...... 000
NS.......................... 998
NR.......................... 999</td></tr>
<tr><td></td><td>LISTA DE NOMES</td><td colspan="2">PARENTESCO</td><td>SEXO</td><td>IDADE</td></tr>
<tr><td>1</td><td>___________________________
NOME DA PESSOA IDOSA</td><td colspan="2"></td><td></td><td></td></tr>
<tr><td>2</td><td></td><td colspan="2">|___|___|</td><td>|___|</td><td>|___|___|___|</td></tr>
<tr><td>3</td><td></td><td colspan="2">|___|___|</td><td>|___|</td><td>|___|___|___|</td></tr>
<tr><td>4</td><td></td><td colspan="2">|___|___|</td><td>|___|</td><td>|___|___|___|</td></tr>
<tr><td>5</td><td></td><td colspan="2">|___|___|</td><td>|___|</td><td>|___|___|___|</td></tr>
<tr><td>6</td><td></td><td colspan="2">|___|___|</td><td>|___|</td><td>|___|___|___|</td></tr>
<tr><td>7</td><td></td><td colspan="2">|___|___|</td><td>|___|</td><td>|___|___|___|</td></tr>
<tr><td>8</td><td></td><td colspan="2">|___|___|</td><td>|___|</td><td>|___|___|___|</td></tr>
<tr><td>9</td><td></td><td colspan="2">|___|___|</td><td>|___|</td><td>|___|___|___|</td></tr>
<tr><td>10</td><td></td><td colspan="2">|___|___|</td><td>|___|</td><td>|___|___|___|</td></tr>
<tr><td>11</td><td></td><td colspan="2">|___|___|</td><td>|___|</td><td>|___|___|___|</td></tr>
<tr><td>12</td><td></td><td colspan="2">|___|___|</td><td>|___|</td><td>|___|___|___|</td></tr>
<tr><td>13</td><td></td><td colspan="2">|___|___|</td><td>|___|</td><td>|___|___|___|</td></tr>
<tr><td>14</td><td></td><td colspan="2">|___|___|</td><td>|___|</td><td>|___|___|___|</td></tr>
<tr><td>15</td><td></td><td colspan="2">|___|___|</td><td>|___|</td><td>|___|___|___|</td></tr>
<tr><td>16</td><td></td><td colspan="2">|___|___|</td><td>|___|</td><td>|___|___|___|</td></tr>
</table>

**G.2**	Então são |___|___| pessoas que residem nesta casa? Conferir, se for diferente do citado verifique e corrija a lista.

Número de registro	G. 13a<br>**AGORA DIGA-ME SE *(nome)* DE ALGUMA FORMA O(A) AJUDA. SE SIM, COM QUE?**	G. 15a<br>**AGORA DIGA-ME SE O(A) SR(A) OFERECE ALGUM TIPO DE AJUDA PARA *(nome)*? SE SIM, COM QUE?**
	Sim........ **1**<br>Não .... **2**<br>NS .... **8**<br>NR **9**	Sim........ **1**<br>Não .... **2**<br>NS .... **8**<br>NR **9**
	AJUDA RECEBIDA	AJUDA FORNECIDA
1	**IDOSO(A)**	**IDOSO(A)**
2	|____| __________	|____| __________
3	|____| __________	|____| __________
4	|____| __________	|____| __________
5	|____| __________	|____| __________
6	|____| __________	|____| __________
7	|____| __________	|____| __________
8	|____| __________	|____| __________
9	|____| __________	|____| __________
10	|____| __________	|____| __________
11	|____| __________	|____| __________
12	|____| __________	|____| __________
13	|____| __________	|____| __________
14	|____| __________	|____| __________
15	|____| __________	|____| __________
16	|____| __________	|____| __________

FILHOS QUE NÃO MORAM NA CASA (incluir filhos adotivos e enteados)

Número de registro	G.18a	G.19	G.20	G.21	G. 28a	G. 30b
	Agora quero perguntar sobre os seus filhos que **NÃO MORAM COM O(A) SR(A)** nesta casa. Poderia, por favor, me dizer o nome deles.<br>Nº de filhos fora da casa .... |___|___| Não tem filhos fora da casa .... **00**<br>**Vá para G32**<br>NS... **98**<br>NR **99**	*(Nome)* **É SEU FILHO:**<br>Próprio **1**<br>Enteado **2**<br>Adotivo **3**<br>NS................ **8**<br>NR **9**	*(Nome)* **É HOMEM OU MULHER?**<br>HOMEM.... **1**<br>MULHER ... **2**	**QUANTOS ANOS COMPLETOS TEM** *(nome)***?**<br>< de 1 ano.......... **00**<br>NS...................... **98**<br>NR...................... **99**	**AGORA DIGA-ME SE** *(nome)* **DE ALGUMA FORMA O AJUDA. SE SIM, COM QUE?**<br>Sim....................................... **1**<br>Não .... **2**<br>NS .... **8**<br>NR **9**	**AGORA DIGA-ME SE O(A) SR(A) OFERECE ALGUM TIPO DE AJUDA PARA** *(nome)***?**<br>Sim....................................... **1**<br>Não .... **2**<br>NS .... **8**<br>NR **9**
	**Lista de nomes**	**Parentesco**	**Sexo**	**Idade**	**AJUDA RECEBIDA**	**AJUDA FORNECIDA**
1		|___|	|___|	|___|___|	|___|	|___| ....
2		|___|	|___|	|___|___|	|___|	|___| ....
3		|___|	|___|	|___|___|	|___|	|___| ....
4		|___|	|___|	|___|___|	|___|	|___| ....
5		|___|	|___|	|___|___|	|___|	|___| ....
6		|___|	|___|	|___|___|	|___|	|___| ....
7		|___|	|___|	|___|___|	|___|	|___| ....
8		|___|	|___|	|___|___|	|___|	|___| ....
9		|___|	|___|	|___|___|	|___|	|___| ....
10		|___|	|___|	|___|___|	|___|	|___| ....

IRMÃOS E IRMÃS

Número de registro	G.32	G.33	G.34	G. 40a	G. 42a
	Agora quero perguntar sobre seusirmãos e irmãs que **NÃO MORAM COM O(A) SR(A)** nessa casa. Poderia, por favor, me dizer o nome deles.<br>Nº de irmãos vivos fora da casa..|___|___| Não tem irmãos fora da casa .... **00**<br>**Vá para G44a**<br>NS... **98**<br>NR **99**	*(Nome)* **É HOMEM OU MULHER?**<br>HOMEM.... **1**<br>MULHER ... **2**	**QUANTOS ANOS COMPLETOS TEM** *(nome)***?**<br>NS................. **998**<br>NR.................. **999**	**AGORA DIGA-ME SE** *(nome)* **DE ALGUMA FORMA O AJUDA. SE SIM, COM QUE?**<br>Sim.. **1**<br>Não .... **2**<br>NS .... **8**<br>NR **9**	**AGORA DIGA-ME SE O(A) SR(A) OFERECE ALGUM TIPO DE AJUDA PARA** *(nome)***?**<br>Sim.. **1**<br>Não .... **2**<br>NS .... **8**<br>NR **9**
	**Lista de nomes**	**Sexo**	**Idade**	**AJUDA RECEBIDA (especifique)**	**AJUDA FORNECIDA (especifique)**
1		|___|	|___||___|___|	|___|	|___| ....
2		|___|	|___||___|___|	|___|	|___| ....
3		|___|	|___||___|___|	|___|	|___| ....
4		|___|	|___||___|___|	|___|	|___| ....
5		|___|	|___||___|___|	|___|	|___| ....
6		|___|	|___||___|___|	|___|	|___| ....
7		|___|	|___||___|___|	|___|	|___| ....
8		|___|	|___||___|___|	|___|	|___| ....
9		|___|	|___||___|___|	|___|	|___| ....
10		|___|	|___||___|___|	|___|	|___| ....

SEÇÃO G – REDE DE APOIO FAMILIAR E SOCIAL

OUTROS FAMILIARES E AMIGOS

Número de registro	G.44a	G.45	G.44b	G. 48a	G. 50a
	Existe algum outro familiar ou amigo que **NÃO MORAM COM O(A) SR(A)** nesta casa do qual recebe ajuda ou para o qual fornece algum tipo de ajuda. Poderia, por favor, me dizer o nome deles.<br>Sim .... **1**<br>Não .... **2**<br>**Vá para G51** **8**<br>NS........ **9**<br>NR	*(Nome)* **É HOMEM OU MULHER?**<br><br>HOMEM.... **1**<br>MULHER ... **2**	**QUAL O SEU PARENTESCO COM (*nome*)?**<br>**Pais** .... **1**<br>**Sogros** .... **2**<br>**Genro/Nora** .... **3**<br>**Neto(a)** .... **4**<br>**Sobrinho(a)** .... **5**<br>**Outro** .... **6**<br>**Nenhum** .... **7**<br>**NS** .... **8**<br>**NR** **9**	**AGORA DIGA-ME SE *(nome)* DE ALGUMA FORMA O AJUDA. SE SIM, COM QUE?**<br><br>Sim........ **1**<br>Não .... **2**<br>NS .... **8**<br>NR **9**	**AGORA DIGA-ME SE O(A) SR(A) OFERECE ALGUM TIPO DE AJUDA PARA *(nome)*?**<br><br>Sim........ **1**<br>Não .... **2**<br>NS .... **8**<br>NR **9**
	**Lista de nomes**	**Sexo**	**Parentesco**	**AJUDA RECEBIDA (especifique)**	**AJUDA FORNECIDA (especifique)**
1		|___|	|___|	|___|	|___| ....
2		|___|	|___|	|___|	|___| ....
3		|___|	|___|	|___|	|___| ....
4		|___|	|___|	|___|	|___| ....
5		|___|	|___|	|___|	|___| ....
6		|___|	|___|	|___|	|___| ....
7		|___|	|___|	|___|	|___| ....
8		|___|	|___|	|___|	|___| ....
9		|___|	|___|	|___|	|___| ....
10		|___|	|___||___|___|	|___|	|___| ....

G.51 - APGAR DE FAMILIA (APLICAR SOMENTE COM O(A) IDOSO(A))

AS SEGUINTES PERGUNTAS FORAM ELABORADAS PARA NOS AJUDAR A COMPREENDER O(A) SR(A) E SUA FAMÍLIA. SINTA-SE À VONTADE PARA FAZER PERGUNTAS SOBRE QUALQUER ITEM DO QUESTIONÁRIO.

"FAMÍLIA" É (SÃO) A(S) PESSOA(S) COM A(S) QUAL(AIS) HABITUALMENTE SE VIVE. CASO O(A) IDOSO(A) MORE SOZINHO(A), CONSIDERE FAMÍLIA AQUELAS PESSOAS COM AS QUAIS ELE(A) TEM LAÇOS EMOCIONAIS MAIS FORTES. (PARA O IDOSO)

		SEMPRE	QUASE SEMPRE	ALGUMAS VEZES	RARAMENTE	NUNCA	NR
**G.51b**	Estou satisfeito(a) pois posso recorrer à minha família em busca de ajuda quando alguma coisa está me incomodando ou preocupando.	**4**	**3**	**2**	**1**	**0**	**9**
**G.51c**	Estou satisfeito(a) com a maneira pela qual minha família e eu conversamos e compartilhamos os problemas.	**4**	**3**	**2**	**1**	**0**	**9**
**G.51d**	Estou satisfeito(a) com a maneira como minha família aceita e apóia meus desejos de iniciar ou buscar novas atividades e procurar novos caminhos ou direções.	**4**	**3**	**2**	**1**	**0**	**9**
**G.51e**	Estou satisfeito(a) com a maneira pela qual minha família demonstra afeição e reage às minhas emoções, tais como raiva, mágoa ou amor.	**4**	**3**	**2**	**1**	**0**	**9**
**G.51f**	Estou satisfeito(a) com a maneira pela qual minha família e eu compartilhamos o tempo juntos.	**4**	**3**	**2**	**1**	**0**	**9**

PARTICIPAÇÃO COMUNITÁRIA E TRANSFERÊNCIAS

<table>
<tr><td rowspan="2">Número de registro</td><td>G. 52
Nos últimos 12 meses (último ano) o(a) Sr(a) RECEBEU assistência ou ajuda de algum tipo de instituição ou organização de sua comunidade? Se sim, anote todos os códigos correspondentes.
UTILIZE UMA LINHA PARA CADA INSTITUIÇÃO OU ORGANIZAÇÃO.</td><td colspan="3">G. 53
QUE TIPO DE AJUDA O(A) SR(A) RECEBEU?

RELACIONE A AJUDA COM A INSTITUIÇÃO CITADA.</td><td colspan="3">G.54a
QUAL A FREQÜÊNCIA COM QUE O(A) SR(A) RECEBEU ESSA AJUDA?

CADA QUADRADINHO CORRESPONDE A UMA AJUDA CITADA EM G.53</td></tr>
<tr><td>SIM
Serviço Social........ 01
Centro de convivência de idosos 02
Bolsa Família 08
Igreja, templo ou outra instituição religiosa ... 04
Pastoral da saúde ou do idoso 07
Outro (especifique)........ 05
NÃO (vá para G.55) 06
NS 98
NR .... 99</td><td colspan="3">DINHEIRO........ 01
COMIDA........ 02
AJUDA EM TAREFAS DOMÉSTICAS........ 03
AJUDA NO CUIDADO PESSOAL 04
TRANSPORTE 05
LAZER/DIVERSÃO 06
COMPANHIA 07
Outro (especifique)........ 08
NS 98
NR .... 99</td><td colspan="3">DIÁRIA 1
SEMANAL........ 2
QUINZENAL 3
MENSAL 4
SEMESTRAL 5
ANUAL 6
ESPORÁDICA 7
NS 8
NR .... 9</td></tr>
<tr><td></td><td>AJUDA RECEBIDA</td><td colspan="3">TIPO DE AJUDA</td><td colspan="3">FREQUENCIA DE RECEBIMENTO DA AJUDA</td></tr>
<tr><td>0</td><td>EXEMPLO:
| 1 | RECEBE AJUDA DO SERVIÇO SOCIAL</td><td>|0|2|
COMIDA</td><td>|0|3|
ROUPA</td><td>|0|5|
TRANSPORTE</td><td>| 2 |
SEMANAL</td><td>| 4 |
MENSAL</td><td>| 2 |
SEMANAL</td></tr>
<tr><td>1</td><td>|___| ____________</td><td>|___|___|</td><td>|___|___|</td><td>|___|___|</td><td>|_____|</td><td>|_____|</td><td>|_____|</td></tr>
<tr><td>2</td><td>|___| ____________</td><td>|___|___|</td><td>|___|___|</td><td>|___|___|</td><td>|_____|</td><td>|_____|</td><td>|_____|</td></tr>
<tr><td>3</td><td>|___| ____________</td><td>|___|___|</td><td>|___|___|</td><td>|___|___|</td><td>|_____|</td><td>|_____|</td><td>|_____|</td></tr>
<tr><td>4</td><td>|___| ____________</td><td>|___|___|</td><td>|___|___|</td><td>|___|___|</td><td>|_____|</td><td>|_____|</td><td>|_____|</td></tr>
<tr><td>5</td><td>|___| ____________</td><td>|___|___|</td><td>|___|___|</td><td>|___|___|</td><td>|_____|</td><td>|_____|</td><td>|_____|</td></tr>
<tr><td>6</td><td>|___| ____________</td><td>|___|___|</td><td>|___|___|</td><td>|___|___|</td><td>|_____|</td><td>|_____|</td><td>|_____|</td></tr>
<tr><td>7</td><td>|___| ____________</td><td>|___|___|</td><td>|___|___|</td><td>|___|___|</td><td>|_____|</td><td>|_____|</td><td>|_____|</td></tr>
<tr><td>8</td><td>|___| ____________</td><td>|___|___|</td><td>|___|___|</td><td>|___|___|</td><td>|_____|</td><td>|_____|</td><td>|_____|</td></tr>
<tr><td>9</td><td>|___| ____________</td><td>|___|___|</td><td>|___|___|</td><td>|___|___|</td><td>|_____|</td><td>|_____|</td><td>|_____|</td></tr>
<tr><td>10</td><td>|___| ____________</td><td>|___|___|</td><td>|___|___|</td><td>|___|___|</td><td>|_____|</td><td>|_____|</td><td>|_____|</td></tr>
</table>

PARTICIPAÇÃO COMUNITÁRIA E TRANSFERÊNCIAS

<table>
<tr><td rowspan="2">Número de registro</td><td>G. 55 - Nos últimos 12 meses (último ano) o(a) Sr(a) PRESTOU ALGUM SERVIÇO de forma voluntária ou gratuita para algum tipo de instituição ou organização de sua comunidade?
UTILIZE UMA LINHA PARA CADA INSTITUIÇÃO OU ORGANIZAÇÃO.</td><td colspan="3">G. 56
QUE TIPO DE AJUDA O(A) SR(A) FORNECE ÀS ORGANIZAÇÕES OU INSTITUIÇÕES DE SUA COMUNIDADE?

RELACIONE A AJUDA COM A INSTITUIÇÃO CITADA.</td><td colspan="3">G.57a
QUAL A FREQÜÊNCIA COM QUE O(A) SR(A) OFERECE ESSA AJUDA?

CADA QUADRADINHO CORRESPONDE A UMA AJUDA CITADA EM G.56</td></tr>
<tr><td>SIM
Serviço Social.......... 01
Centro de convivência de idosos 02
Creche ou centro infantil.......... 03
Colégio ou Universidade 04
Posto de Saúde 05
Igreja, templo ou outra instituição religiosa 06
Hospital 07
Pastoral da saúde ou do idoso 10
Outro (especifique).......... 08
NÃO (vá para Seção H) 09
NS 98
NR .... 99</td><td colspan="3">DINHEIRO.......... 01
COMIDA.......... 02
ROUPA 03
AJUDA COM ENSINO 06
AJUDA NAS OFICINAS 07
LAZER/DIVERSÃO 09
COMPANHIA 10
OUTRO (especifique).......... 08
NS 98
NR .... 99</td><td colspan="3">DIÁRIA.......... 1
SEMANAL.......... 2
QUINZENAL 3
MENSAL 4
SEMESTRAL 5
ANUAL 6
ESPORÁDICA 7
NS 8
NR .... 9</td></tr>
<tr><th></th><th>AJUDA PRESTADA</th><th colspan="3">TIPO DE AJUDA</th><th colspan="3">FREQUENCIA DE RECEBIMENTO DA AJUDA</th></tr>
<tr><td>0</td><td>EXEMPLO:
|10| PRESTOU SERVIÇO NA PASTORAL DO IDOSO</td><td>|0|9|
LAZER</td><td>|1|0|
COMPANHIA</td><td>|0|8|
ORAÇÃO</td><td>| 5 |
SEMESTRAL</td><td>| 2 |
SEMANAL</td><td>| 2 |
SEMANAL</td></tr>
<tr><td>1</td><td>|___| ____________________</td><td>|___|___|</td><td>|___|___|</td><td>|___|___|</td><td>|_____|</td><td>|_____|</td><td>|_____|</td></tr>
<tr><td>2</td><td>|___| ____________________</td><td>|___|___|</td><td>|___|___|</td><td>|___|___|</td><td>|_____|</td><td>|_____|</td><td>|_____|</td></tr>
<tr><td>3</td><td>|___| ____________________</td><td>|___|___|</td><td>|___|___|</td><td>|___|___|</td><td>|_____|</td><td>|_____|</td><td>|_____|</td></tr>
<tr><td>4</td><td>|___| ____________________</td><td>|___|___|</td><td>|___|___|</td><td>|___|___|</td><td>|_____|</td><td>|_____|</td><td>|_____|</td></tr>
<tr><td>5</td><td>|___| ____________________</td><td>|___|___|</td><td>|___|___|</td><td>|___|___|</td><td>|_____|</td><td>|_____|</td><td>|_____|</td></tr>
<tr><td>6</td><td>|___| ____________________</td><td>|___|___|</td><td>|___|___|</td><td>|___|___|</td><td>|_____|</td><td>|_____|</td><td>|_____|</td></tr>
<tr><td>7</td><td>|___| ____________________</td><td>|___|___|</td><td>|___|___|</td><td>|___|___|</td><td>|_____|</td><td>|_____|</td><td>|_____|</td></tr>
<tr><td>8</td><td>|___| ____________________</td><td>|___|___|</td><td>|___|___|</td><td>|___|___|</td><td>|_____|</td><td>|_____|</td><td>|_____|</td></tr>
<tr><td>9</td><td>|___| ____________________</td><td>|___|___|</td><td>|___|___|</td><td>|___|___|</td><td>|_____|</td><td>|_____|</td><td>|_____|</td></tr>
<tr><td>10</td><td>|___| ____________________</td><td>|___|___|</td><td>|___|___|</td><td>|___|___|</td><td>|_____|</td><td>|_____|</td><td>|_____|</td></tr>
</table>

SEÇÃO H - HISTÓRIA DE TRABALHO E FONTES DE RECEITA

H.1a

Alguma vez, na sua vida, o(a) Sr.(a) teve algum trabalho, pelo qual recebeu um pagamento em dinheiro ou em espécie ou ajudou em um estabelecimento familiar, sem receber qualquer tipo de pagamento?

H. 1

SIM, trabalhei **COM** pagamento em dinheiro ou espécie	1	
SIM, trabalhei **SEM** receber pagamento *(Ex.: estabelecimento familiar)*	2	
NÃO, nunca trabalhei	3	VÁ PARA **H. 3**
NS	8	VÁ PARA **H. 3**
NR	9	

H.12

Com relação ao seu trabalho principal, o Sr.(a) é ou era...?

LEIA AS OPÇÕES ATÉ OBTER UMA RESPOSTA AFIRMATIVA

H. 2

OPERÁRIO OU EMPREGADO NÃO AGROPÉCUÁRIO?	01	VÁ PARA **H. 4**
DIARISTA RURAL OU PEÃO DO CAMPO?	02	
PATRÃO, EMPREGADOR OU PROPRIETÁRIO (C/ 1 A 5 EMPREGADOS)?.	03	
PATRÃO, EMPREGADOR OU PROPRIETÁRIO (C/6 OU MAIS EMPREGADOS)?	04	
TRABALHADOR POR CONTA PRÓPRIA?.	05	
TRABALHADOR FAMILIAR, SEM PAGAMENTO?.	06	
TRABALHADOR NÃO FAMILIAR, SEM PAGAMENTO?	07	
TRABALHADOR POR EMPREITADA?	08	
OUTRO *Especifique*	09	
NS	98	
NR	99	

H.3

Qual a principal razão pela qual o(a) sr.(a) nunca trabalhou?

ANOTE SOMENTE UMA RESPOSTA

H. 3

PROBLEMA DE SAÚDE	1	VÁ PARA **H.10**
NÃO TINHA NECESSIDADE ECONÔMICA	2	
DEDICOU-SE A CUIDAR DA FAMÍLIA	3	
CASOU-SE MUITO JOVEM	4	
NÃO HAVIA OPORTUNIDADE DE TRABALHO	5	
OS PAIS NÃO DEIXARAM	6	
OUTRO ________ *Especifique*	7	
NS	8	
NR	9	

H.4

Que idade o(a) Sr(a) tinha quando começou a trabalhar?

H. 4

ANOS	|___|___| anos
NS	98
NR	99

SEÇÃO H - HISTÓRIA DE TRABALHO E FONTES DE RECEITA

H.5a

Atualmente o(a) Sr(a) trabalha?

H. 5

TRABALHA	1
NÃO TRABALHA	2 → VÁ PARA **H. 8**

H.5b

Tipo de trabalho atual:

ANOTE TODAS AS RESPOSTAS DO(A IDOSO(A)

H. 6

TRABALHO VOLUNTÁRIO (SEM REMUNERAÇÃO)	1
TRABALHO ESPORÁDICO (BICO)	2
TRABALHO REGULAR	3
NR	9

H. 22

Qual é a principal razão pela qual o(a) Sr.(a) trabalha?

ANOTE SOMENTE UMA RESPOSTA (A MAIS IMPORTANTE)

H. 7

NECESSITA DO DINHEIRO	1
NECESSITA OU QUER AJUDAR A FAMÍLIA	2
QUER MANTER-SE OCUPADO	3
NECESSIDADE DE SENTIR-SE ÚTIL, PRODUTIVO	4
GOSTA DO SEU TRABALHO	5
OUTRO ______ *(Especifique)*	6
AINDA NÃO COMPLETEI TEMPO/IDADE PARA ME APOSENTAR	7
NS	8
NR	9

VÁ PARA **H. 10**

H.8

Qual a principal razão pela qual o(a) Sr.(a) não trabalha atualmente?

ANOTE SOMENTE UMA RESPOSTA

H. 8

NÃO CONSEGUE TRABALHO	1
PROBLEMAS DE SAÚDE	2
APOSENTADO	3
FOI COLOCADO À DISPOSIÇÃO	4
A FAMÍLIA NÃO QUER QUE TRABALHE	5
OUTRO ______ *Especifique*	6
NS	8
NR	9

H.7

Com que idade deixou de trabalhar?

H. 9

ANOS	**|___|___| anos**
NS	98
NR	99

H.27c
O(a) Sr(a) recebe algum tipo de alguma receita (dinheiro) próprio?

H. 10

SIM	1	
NÃO	2	VÁ PARA **H.15**
NS	8	VÁ PARA **H.15**
NR	9	VÁ PARA **H.15**

H.26a
Que tipo de receita (dinheiro) o(a) Sr(a) recebe?

ANOTAR ATÉ 2 RESPOSTAS.

H. 11

APOSENTADORIA POR TEMPO DE SERVIÇO	01
APOSENTADORIA POR DOENÇA OU INVALIDEZ	02
PENSÃO (do cônjuge, dos filhos, dos pais, etc)	03
BENEFÍCIO DE PRESTAÇÃO CONTINUADA (BPC)	04
FUNRURAL	05
ALUGUEL OU APLICAÇÕES BANCÁRIAS	06
SALÁRIO	08
OUTRO ______ (*ESPECIFIQUE*)	07
NS	88
NR	89

H.26a1
O(a) Sr(a) recebe 13º salário?

H. 12

SIM	1
NÃO	2
NS	8
NR	9

H.26c
No total, aproximadamente, quanto o(a) Sr(a) recebe por mês?

Referência: 1 salário mínimo = R$ 622,00

ANOTAR ATÉ 2 RESPOSTAS.

H. 13

VALOR: |__|__|__|__|__|,|__|__|

MENOS DE UM SALÁRIO MÍNIMO	1
UM SALÁRIO MÍNIMO	2
ATÉ DOIS SALÁRIOS MÍNIMOS	3
DE DOIS A QUATRO SALÁRIOS MÍNIMOS	4
DE QUATRO A DEZ SALÁRIOS MÍNIMOS	5
DE DEZ A VINTE SALÁRIOS MÍNIMOS	6
VINTE SALÁRIOS MÍNIMOS E MAIS	7
NS	8
NR	9

H.28
Quantas pessoas dependem dos seus ganhos?

INCLUA O ENTREVISTADO NO TOTAL DE PESSOAS QUE DEPENDEM DESTES GANHOS.

H. 14

SÓ O(a) ENTREVISTADO(a)	01
NÚMERO DE PESSOAS some o(a) idoso(a) às pessoas citadas	|__|__|
NS	98
NR	99

**Em relação a algumas despesas do dia-a-dia, gostaria que o(a) Sr(a) me dissesse como as paga:**	O SR. PAGA		OUTRA PESSOA responsável pelo pagto.	NÃO PAGA (nem o(a) idoso(a) nem outras pessoas)	NR	NR
	Sim, todo	Sim, em parte				
H.29a Casa (luz, gás, telefone, água, IPTU, aluguel, etc.) **H.15**	1	2	3	4	8	9
H.29h Comida, roupas, material de limpeza, etc **H.16**	1	2	3	4	8	9
H.29d Medicamentos, despesas com médicos, etc. **H.17**	1	2	3	4	8	9
H.29f Outros ____ ____ ____ *Especifique* **H.18**	1	2	3	4	8	9

H.30
O(a) Sr(a) considera que tem dinheiro suficiente para cobrir suas despesas diárias?

H. 19

SIM 1
NÃO 2
NS 8
NR 9

As perguntas H20 e H21 são para o(a)s idoso(a)s que trabalharam/trabalham.
Se ele(a) NUNCA trabalhou, vá para a SEÇÃO J.

H.19
Alguma vez um médico ou enfermeira lhe disse que o(a) Sr(a) tinha ou tem um problema de saúde provocado pelo trabalho ou ocupação que exerceu em sua vida?

H. 20

SIM 1
NÃO 2
NS 8
NR 9

(NÃO, NS, NR:) VÁ PARA **J.01a**

H.20
Que tipo de problema é este?

H. 21

Textual ________________ |__|__|
____
____
NS 98
NR 99

SEÇÃO J- CARACTERÍSTICAS DA MORADIA

J. 1a<br>A CASA ONDE O(A) IDOSO(A) MORA É:<br>J01		
	TÉRREA	4
	SOBRADO	6
	APARTAMENTO...	2
	BARRACO	3
	OUTRO ....<br>(especifique)	5

J.2<br>A RESIDENCIA ONDE O(A) IDOSO(A) MORA É:<br>J02		
	PRÓPRIA, QUITADA, EM TERRENO PRÓPRIO...........	1
	AINDA ESTÁ PAGANDO	2
	PRÓPRIA EM TERRENO QUE NÃO É PRÓPRIO	3
	ARRENDADA OU ALUGADA	4
	EMPRESTADA OU CEDIDA	5
	OUTRO ....<br>(especifique)	6
	NS	8
	NR	9

A CASA ONDE O(A) IDOSO(A) MORA TEM:	SIM	NÃO	NR
J.3a LUZ ELÉTRICA (J03)	**1**	**2**	**9**
J.4a ÁGUA ENCANADA (J04)	**1**	**2**	**9**
J.5a SISTEMA DE DRENAGEM DE ESGOTO (J05)	**1**	**2**	**9**
J.8 CÔMODO PRÓPRIO PARA COZINHAR (J06)	**1**	**2**	**9**

J.7 NÚMERO DE QUARTOS (J07)	|____|____|	9

J13. NESTA CASA HÁ:

**POSSE DE ITENS** (J08)	**NÃO TEM**	**TEM (Quantidade)**			
		**UM**	**DOIS**	**TRÊS**	**QUATRO**
Televisores em cores	0	1	2	3	4
Videocassete /DVD	0	2	2	2	2
Rádios	0	1	2	3	4
Banheiros	0	4	5	6	7
Automóveis	0	4	7	9	9
Empregadas mensalistas	0	3	4	4	4
Máquinas de lavar	0	2	2	2	2
Geladeira	0	4	4	4	4
Freezer (*)	0	2	2	2	2
**TOTAL DE PONTOS**	J08 |___|___| (preenchido pela crítica)				

(*) Independente ou 2ª porta da geladeira

SEÇÃO J- CARACTERÍSTICAS DA MORADIA

POSSE DE ITENS J08	NÃO TEM	TEM (Quantidade)			
		UM	DOIS	TRÊS	QUATRO
Telefone móvel	0	1	2	3	4
Telefone fixo	0	2	2	2	2
Fogão	0	1	2	3	4
Computador	0	4	5	6	7
TV a cabo	0	4	7	9	9
Internet	0	3	4	4	4
Motocicleta	0	2	2	2	2
**TOTAL DE PONTOS**	J08 |___|___| (preenchido pela crítica)				

AVALIAÇÃO DO RISCO AMBIENTAL

AS QUESTÕES A SEGUIR DEVEM SER RESPONDIDAS DE ACORDO COM A OBSERVAÇÃO DA ENTREVISTADORA.

J. 14<br>O(A) idoso(a) :<br>J09	
CONSEGUE ANDAR...	1
É ACAMADO	2
UTILIZA CADEIRA DE RODAS PARA SE LOCOMOVER	3

Na entrada da casa/prédio onde o(a) idoso(a) mora observa-se a presença de:	SIM	NÃO
J.17a ESCADAS J10	**1**	**2**
J.17b RAMPAS J11	**1**	**2**
J.17c CORRIMÃO J12	**1**	**2**

J. 20<br>HÁ QUANTO TEMPO O(A) SR(A) MORA NESSA CASA/APARTAMENTO?<br>J13	
Menos de 1 ano	1
Mais de 1 ano	2
NS	8
NR	9

J. 21<br>QUANDO ESTÁ EM SUA CASA/APARTAMENTO, **<u>DURANTE O DIA</u>**, QUE TIPO DE CALÇADO NORMALMENTE O(A) SR(A) COSTUMA UTILIZAR?<br>Assinale o que for mais freqüente (apenas um).<br>J14	
Sapato fechado COM solado de borracha	1
Sapato fechado SEM solado de borracha	2
Chinelos ou tamancos	3
Anda descalço, COM meias	4
Anda descalço, SEM meias	5
OUTRO.. (especifique)	6
NS	8
NR	9

SEÇÃO J- CARACTERÍSTICAS DA MORADIA

J. 22<br>QUANDO LEVANTA, **DURANTE A NOITE**, QUE TIPO DE CALÇADO NORMALMENTE O(A) SR(A) COSTUMA UTILIZAR?<br><br>Assinale o que for mais freqüente (apenas um).<br><br>**J15**	Sapato fechado COM solado de borracha .... 1<br>Sapato fechado SEM solado de borracha .... 2<br>Chinelos ou tamancos .... 3<br>Anda descalço, COM meias .... 4<br>Anda descalço, SEM meias .... 5<br>OUTRO.. 6<br>(especifique)<br>Não costuma se levantar à noite .... 7<br>NS .... 8<br>NR 9

AS QUESTÕES A SEGUIR DEVEM SER RESPONDIDAS SE **EXISTIR ESCADA** NO INTERIOR DA CASA/APARTAMENTO.

J. 23a<br>Há escadas no interior da casa/apartamento onde o(a) idoso(a) reside?<br><br>**J16**	Sim .... 1<br>Não .... 2 Vá para J.26<br>NS .... 8<br>NR 9

A(s) escada(s) são:		**SIM**	**NÃO**
J.23b1 Retas	**J17**	1	2
J.23b2 Em curva ou caracol	**J18**	1	2
J.23b3 COM corrimão (apenas 1 lado)	**J19**	1	2
J.23b4 COM corrimão (dos 2 lados)	**J20**	1	2
J.23b5 SEM corrimão	**J21**	1	2
J.23b6 Iluminadas	**J22**	1	2
J.23b7 Acarpetadas	**J23**	1	2
J.23b8 Enceradas ou com piso escorregadio	**J24**	1	2
J.23b9 Com objetos espalhados pelos degraus (livros, vasos, sapatos, etc)	**J25**	1	2

AS QUESTÕES A SEGUIR REFEREM-SE AO LOCAL, NO INTERIOR DA CASA/APARTAMENTO, ONDE O(A) IDOSO(A) COSTUMA **TOMAR BANHO**.

J. 36a<br>O(a) idoso(a) usualmente toma banho:<br><br>**J26**	No banheiro (chuveiro ou banheira) .... 1<br>No banheiro (sentado no vaso sanitário) .... 2<br>No leito (na cama) .... 3 Vá para J43<br>NS .... 8<br>NR 9

SEÇÃO J- CARACTERÍSTICAS DA MORADIA

No local onde o(a) idoso(a) toma banho há:		SIM	NÃO	Não se aplica
J.36b Chuveiro	**J27**	1	2	3
J.36c Banheira	**J28**	1	2	3
J.36d Barra de apoio no interior do local onde está o chuveiro/banheira	**J29**	1	2	3
J.36e tapete de borracha ou piso antiderrapante no interior do local onde está o chuveiro/banheira	**J30**	1	2	3
J.36f Banco ou cadeira para banho	**J31**	1	2	3
J.36g Barra de apoio ao lado do vaso sanitário	**J32**	1	2	3

AS QUESTÕES A SEGUIR REFEREM-SE AO(S) LOCAL(IS), NO INTERIOR DA CASA/APARTAMENTO, POR ONDE O(A) IDOSO(A) COSTUMA **CIRCULAR OU ANDAR**.

No local por onde o(a) idoso(a) costuma andar/ circular observa(m)-se:		SIM	NÃO	NR
J.29 Piso escorregadio ou encerado	**J33**	1	2	9
J.30 Tapetes soltos	**J34**	1	2	9
J.31 Objetos desordenados (livros, vasos, sapatos, papéis, etc)	**J35**	1	2	9
J.32 Móveis muito pesados	**J36**	1	2	9
J.33 Piso com diferença(s) de nível (degraus)	**J37**	1	2	9

J. 34

O(a) Sr(a) costuma se apoiar nos moveis ou parede para se locomover de um cômodo à outro no interior da casa/apartamento?

J38

Sim 1
Não 2
NS 8
NR 9

J. 35

O(a) Sr(a) deixa de ir a algum cômodo no interior da casa/apartamento por sentir dificuldade de acessá-lo?

J39

Sim 1
Não 2
NS 8
NR 9

SEÇÃO J- CARACTERÍSTICAS DA MORADIA

AS QUESTÕES A SEGUIR REFEREM-SE ÀS CALÇADAS OU RUAS, EXTERNAS A CASA/APARTAMENTO, POR ONDE O(A) IDOSO(A) COSTUMA(VA) CIRCULAR OU ANDAR.

J. 36_2<br>O(a) Sr(a) costuma sair à rua para passear ou caminhar (com ou sem outras pessoas)?<br>**J40**	Sim .... 1<br>Não .... 2<br>NS .... 8<br>NR 9
J. 36_3<br>Com que freqüência as **CONDIÇÕES DA RUA OU DAS CALÇADAS** foram impedimento para que o(a) Sr(a) saísse para caminhar ou passear (aclive, declive, presença de buracos ou irregularidades)?<br>**J41**	SEMPRE .... 1<br>ÀS VEZES .... 2<br>NUNCA .... 3<br>NS .... 8<br>NR 9
J. 36_4<br>Com que freqüência **O MEDO DA VIOLÊNCIA** foi impedimento para que o(a) Sr(a) saísse à rua (medo de assalto, atropelamento, etc)?<br>**J42**	SEMPRE .... 1<br>ÀS VEZES .... 2<br>NUNCA .... 3<br>NS .... 8<br>NR 9

AS QUESTÕES A SEGUIR REFEREM-SE À PRESENÇA DE ANIMAIS DOMÉSTICOS NO INTERIOR DA CASA/APARTAMENTO **PERTENCENTES** AO(A) IDOSO(A).

J. 43<br>O(a) Sr(a) possui animais domésticos?<br>**J43**	Sim .... 1<br>Não .... 2 Vá p/ a seção M<br>NS .... 8<br>NR 9
J. 43a<br>Quantos e de que tipo são seus animais domésticos?<br>**J44**	Gato(s) .... 1<br>Número: |___|___| Cachorro(s) .... 2<br>Número: |___|___| Ave(s) .... 3<br>Número: |___|___| Outro(s) .... 4<br>Número: |___|___| NS .... 8<br>NR 9

SEÇÃO J- CARACTERÍSTICAS DA MORADIA

J. 43b<br>O(a) Sr(a) costuma sair de casa por causa de seu(s) animal(is) doméstico(s) (para comprar comida, levar para passear, levar ao veterinário, etc?<br>**J45**	Sim ...... 1<br>Não ...... 2<br>Outra pessoa faz ...... 3<br>NS ...... 8<br>NR 9
J. 43c<br>O(a) Sr(a), alguma vez, mudou sua rotina por causa de seu(s) animal(is) doméstico(s) (deixou de viajar ou sair, receber pessoas, etc)?<br>**J46**	Sim ...... 1<br>Não ...... 2<br>NS ...... 8<br>NR 9
J. 43d<br>A presença de animal(is) doméstico(s) interfere no seu estado de humor (sente-se mais ou menos solitário, sente-se mais ou menos irritado, etc)?<br>**J47**	Sim, positivamente ...... 1<br>Sim, negativamente ...... 2<br>Não ...... 3<br>NS ...... 8<br>NR 9
J. 43e<br>A presença de animal(is) doméstico(s) interfere no(s) seu relacionamento(s) com amigo(s) ou familiares (visitam mais ou menos, viaja mais ou menos, etc)?<br>**J48**	Sim, positivamente ...... 1<br>Sim, negativamente ...... 2<br>Não ...... 3<br>NS ...... 8<br>NR 9
J. 43f<br>Quão **IMPORTANTE** para o(a) Sr(a) é seu animal(is) de estimação?<br>**J49**	MUITO IMPORTANTE ...... 1<br>IMPORTANTE ...... 2<br>INDIFERENTE ...... 3<br>POUCO IMPORTANTE ...... 4<br>NADA IMPORTANTE ...... 5<br>NS ...... 8<br>NR 9

Observações:____

____

____

____

____

____

SEÇÃO M. MAUS TRATOS

FAREMOS AGORA ALGUMAS PERGUNTAS SOBRE A SUA RELAÇÃO COM AS PESSOAS QUE O RODEIAM E A ATITUDE QUE ESSAS PESSOAS COSTUMAM TER COM O(A) SR(A).

M.1
No último ano, alguma das pessoas que o rodeiam tem gritado com o(a) Sr(a) SEM RAZÃO?

SIM	1	
NÃO	2	VÁ PARA M.3
NS	8	VÁ PARA M.3
NR	9	VÁ PARA M.3

M.2
Com que freqüência?

TODOS OS DIAS	1
DUAS OU TRÊS VEZES POR SEMANA	2
UMA VEZ POR SEMANA	3
DUAS A TRÊS VEZES POR MES	4
UMA VEZ POR MÊS OU MENOS	5
NS	8
NR	9

M.3
No último ano, alguma das pessoas que o rodeiam o(a) tem chamado por algum nome ou apelido que o(a) Sr(a) não goste?

SIM	1	
NÃO	2	VÁ PARA M.5
NS	8	VÁ PARA M.5
NR	9	VÁ PARA M.5

M.4
Com que freqüência?

TODOS OS DIAS	1
DUAS OU TRÊS VEZES POR SEMANA	2
UMA VEZ POR SEMANA	3
DUAS A TRÊS VEZES POR MES	4
UMA VEZ POR MÊS OU MENOS	5
NS	8
NR	9

M.5
No último ano, alguma das pessoas que o rodeiam tem usado ou mexido no seu dinheiro sem a sua autorização?

SIM	1	
NÃO	2	VÁ PARA M.7
NS	8	VÁ PARA M.7
NR	9	VÁ PARA M.7

M.6
Com que freqüência?

TODOS OS DIAS	1
DUAS OU TRÊS VEZES POR SEMANA	2
UMA VEZ POR SEMANA	3
DUAS A TRÊS VEZES POR MES	4
UMA VEZ POR MÊS OU MENOS	5
NS	8
NR	9

SEÇÃO M. MAUS TRATOS

M.7
No último ano, alguma das pessoas que o rodeiam o(a) tem ameçado(a) por não fazer o que eles querem que o(a) Sr(a) faça?

SIM 1
NÃO 2
NS 8
NR 9
(2, 8, 9) VÁ PARA **M.9**

M.8
Com que freqüência?

TODOS OS DIAS 1
DUAS OU TRÊS VEZES POR SEMANA 2
UMA VEZ POR SEMANA 3
DUAS A TRÊS VEZES POR MES 4
UMA VEZ POR MÊS OU MENOS 5
NS 8
NR 9

M.9
No último ano, alguma das pessoas que o rodeiam tem batido ou esbofeteado o(a) Sr(a)?

SIM 1
NÃO 2
NS 8
NR 9
(2, 8, 9) VÁ PARA **M.11**

M.10
Com que freqüência?

TODOS OS DIAS 1
DUAS OU TRÊS VEZES POR SEMANA 2
UMA VEZ POR SEMANA 3
DUAS A TRÊS VEZES POR MES 4
UMA VEZ POR MÊS OU MENOS 5
NS 8
NR 9

M.11
No último ano, alguma das pessoas que o rodeiam tem sacudido ou chacoalhado o(a) Sr(a)?

SIM 1
NÃO 2
NS 8
NR 9
(2, 8, 9) VÁ PARA **M.13**

M.12
Com que freqüência?

TODOS OS DIAS 1
DUAS OU TRÊS VEZES POR SEMANA 2
UMA VEZ POR SEMANA 3
DUAS A TRÊS VEZES POR MES 4
UMA VEZ POR MÊS OU MENOS 5
NS 8
NR 9

SEÇÃO M. MAUS TRATOS

**M.13**<br>**No último ano, alguma das pessoas que o rodeiam tem roubado seu dinheiro ou algum pertence importante para o(a) Sr(a)?**	SIM ..... **1**<br>NÃO ..... **2**<br>NS ..... **8**<br>NR ..... **9**<br>(2, 8, 9: VÁ PARA perguntas e comentários finais)
**M.14**<br>**Com que freqüência?**	TODOS OS DIAS ..... **1**<br>DUAS OU TRÊS VEZES POR SEMANA ..... **2**<br>UMA VEZ POR SEMANA ..... **3**<br>DUAS A TRÊS VEZES POR MES ..... **4**<br>UMA VEZ POR MÊS OU MENOS ..... **5**<br>NS ..... **8**<br>NR **9**

PERGUNTAS E COMENTÁRIOS FINAIS

1. **Observou situação de violência, abandono ou maus tratos de algum integrante da casa com relação ao entrevistado?**	SIM .... 1<br>NÃO .... 2 → VÁ PARA 3
2. **Descreva a situação:**	Textual ____
3. **Teve dificuldades para completar o questionário individual?**	SIM .... 1<br>NÃO .... 2 → VÁ PARA 5
4. **De que tipo?**	Textual ____
5. **Ocorreu alguma situação especial que tenha dificultado seu trabalho durante a entrevista?**	SIM .... 1<br>NÃO .... 2 → VÁ PARA 7
6. **Descreva a situação:**	Textual ____
7. **Mais alguém esteve presente durante a entrevista (além do informante ou substituto)?**	SIM .... 1<br>NÃO .... 2 → VÁ PARA 9

PERGUNTAS E COMENTÁRIOS FINAIS

Pergunta	Resposta
8. **Que relação tinham com a pessoa entrevistada e que tipo de participação tiveram durante a entrevista?**	Textual ____________
9. **Houve recusa e oposição da pessoa entrevistada durante o desenvolvimento da entrevista?**	SIM .... 1<br>NÃO .... 2 → VÁ PARA 11
10. **Em que parte ou seção da entrevista?**	Textual ____________
11. **Em que horário e dia da semana considera que é mais fácil encontrar a pessoa entrevistada, caso seja necessário um novo contato.**	Textual ____________

SEÇÃO N - CUIDADORES

ESSA SEÇÃO DEVERÁ SER PREENCHIDA PARA AQUELES IDOSOS QUE **REFERIRAM RECEBER AJUDA** NO DESEMPENHO DE SUAS ATIVIDADES DE VIDA DIÁRIA (SEÇÃO D). ELAS DEVEM SER FEITAS AO "CUIDADOR" MAIS CITADO PELO(A) IDOSO(A) OU SEJA, AQUELA PESSOA QUE MAIS O AJUDA NO DESEMPENHO DESSAS ATIVIDADES.

FAÇA A ENTREVISTA COM O CUIDADOR **SEM** A PRESENÇA DA PESSOA IDOSA.

NOME DO CUIDADOR ENTREVISTADO: ....

N. 1a<br>O(A) cuidador(a) é :	HOMEM ....<br>MULHER	1<br>2
N. 1b<br>O(A) cuidador(a) nasceu em que ano /tem qual idade?	Ano de nascimento |____|____|____|____| Idade |____|____| anos<br>NR..	 <br>9
N. 1c<br>O(A) cuidador(a) é :	SOLTEIRO(A) ....<br>CASADO(A) / AMASIADO(A) ....<br>SEPARADO(A)/ DESQUITADO(A)/ DIVORCIADO(A) ..<br>VIÚVO(A) ....<br>NR..	1<br>2<br>3<br>4<br>9
N. 1d<br>O(A) cuidador(a) sabe ler e escrever um recado :	Sim ....<br>Não ....<br>NR..	1<br>2<br>9
N. 1e<br>Qual o tipo de relação do(a) cuidador(a) com o(a) idoso(a) :	Esposo(a) / companheiro(a)....................................<br>Filho(a)..<br>Pais...<br>Sogros...<br>Irmãos...<br>Genro ou Nora...<br>Neto(a)..<br>Enteado(a)..<br>Outro familiar não remunerado...............................<br>Outro familiar remunerado.....................................<br>Outro não familiar não remunerado........................<br>Empregado Doméstico...<br>Cuidador ....<br>NS...<br>NR	01<br>02<br>03<br>04<br>05<br>06<br>07<br>08<br>09<br>10<br>11<br>12<br>13<br>98<br>99
N. 1f<br>Onde reside o(a) cuidador(a) ?	Na mesma casa junto com o(a) idoso(a) ....<br>Em outra casa próxima à casa do(a) idoso(a) ....<br>Em outra casa distante da casa do(a) idoso(a) ....<br>NR..	1<br>2<br>3<br>9

SEÇÃO N - CUIDADORES

N. 1g<br>Com que freqüência o(a) cuidador(a) auxilia o(a) idoso(a) no desempenho de suas atividades de vida diária ?	O tempo todo .... 1<br>Uma ou mais vezes ao dia .... 2<br>Em dias alternados .... 3<br>Pelo menos 1 vez por semana .... 4<br>Sempre que necessário .... 5<br>Outro........ 6<br>(especifique)<br>NR........ 9

N. 1h<br>O(A) cuidador(a) precisou parar de trabalhar ou estudar para cuidar do(a) idoso(a)?	Sim .... 1<br>Não .... 2<br>NR........ 9

N. 1i<br>Há quanto tempo o(a) cuidador(a) auxilia o(a) idoso(a)?	|____|____| dias<br>|____|____| meses<br>|____|____| anos<br>NR........ 99

N. 1j<br>Há alguém que auxilie o(a) cuidador(a) no cuidado do(a) idoso(a)?	Sim .... 1<br>Não .... 2<br>NR........ 9

APGAR DO CUIDADOR (para ser aplicado em cuidadores familiares)

	AS SEGUINTES PERGUNTAS FORAM ELABORADAS PARA NOS AJUDAR A COMPREENDER O(A) SR(A) E SUA FAMÍLIA. SINTA-SE À VONTADE PARA FAZER PERGUNTAS SOBRE QUALQUER ITEM DO QUESTIONÁRIO.<br>**"FAMÍLIA" É (SÃO) A(S) PESSOA(S) COM A(S) QUAL(AIS) HABITUALMENTE SE VIVE. CONSIDERE FAMÍLIA AQUELAS PESSOAS COM AS QUAIS TEM LAÇOS EMOCIONAIS MAIS FORTES.**	SEMPRE	QUASE SEMPRE	ALGUMAS VEZES	RARAMENTE	NUNCA
**N.23a**	Estou satisfeito(a) pois posso recorrer à minha família em busca de ajuda quando alguma coisa está me incomodando ou preocupando.	**4**	**3**	**2**	**1**	**0**
**N.23b**	Estou satisfeito(a) com a maneira pela qual minha família e eu conversamos e compartilhamos os problemas.	**4**	**3**	**2**	**1**	**0**
**N.23c**	Estou satisfeito(a) com a maneira como minha família aceita e apóia meus desejos de iniciar ou buscar novas atividades e procurar novos caminhos ou direções.	**4**	**3**	**2**	**1**	**0**
**N.23d**	Estou satisfeito(a) com a maneira pela qual minha família demonstra afeição e reage às minhas emoções, tais como raiva, mágoa ou amor.	**4**	**3**	**2**	**1**	**0**
**N.23e**	Estou satisfeito(a) com a maneira pela qual minha família e eu compartilhamos o tempo juntos.	**4**	**3**	**2**	**1**	**0**

SEÇÃO N - CUIDADORES

**A SEGUIR ENCONTRA-SE UMA LISTA DE AFIRMATIVAS QUE REFLETEM COMO AS PESSOAS, ALGUMAS VEZES, PODEM SE SENTIR QUANDO CUIDAM DE OUTRA PESSOA. DEPOIS DE CADA AFIRMATIVA, INDIQUE COM QUE FREQÜÊNCIA O(A) SR(A) SE SENTE EM RELAÇÃO AO QUE ESTÁ SENDO PERGUNTADO. NÃO EXISTEM RESPOSTAS CERTAS OU ERRADAS.**	NUNCA	RARAMENTE	ALGUMAS VEZES	FREQUENTE-MENTE	SEMPRE
N. 1 - **O(a) Sr(a) sente que *NOME DO IDOSO(A)* pede mais ajuda do que ele(a) necessita?**	**0**	**1**	**2**	**3**	**4**
N. 2 - **O(a) Sr(a) sente que por causa do tempo que o (a) Sr(a) gasta com *NOME DO IDOSO(A)* não tem tempo suficiente para si mesmo(a)?**	**0**	**1**	**2**	**3**	**4**
**N. 3 - O(a) Sr(a) se sente estressado(a) entre cuidar de *NOME DO IDOSO(A)* e suas outras responsabilidades com a família e o trabalho?**	**0**	**1**	**2**	**3**	**4**
N. 4 - **O(a) Sr(a) sente envergonhado(a) com o comportamento de *NOME DO IDOSO(A)* ?**	**0**	**1**	**2**	**3**	**4**
**N. 5 - O(a) Sr(a) sente irritado(a) quando *NOME DO IDOSO(A)* está por perto?**	**0**	**1**	**2**	**3**	**4**
**N. 6 - O(a) Sr(a) sente que *NOME DO IDOSO(A)* afeta negativamente seus relacionamentos com outros membros da família ou amigos?**	**0**	**1**	**2**	**3**	**4**
**N. 7 - O(a) Sr(a) sente receio pelo futuro de *NOME DO IDOSO(A)?***	**0**	**1**	**2**	**3**	**4**
**N. 8 - O(a) Sr(a) sente que de *NOME DO IDOSO(A)* depende do(a) Sr(a)*?***	**0**	**1**	**2**	**3**	**4**
**N. 9 - O(a) Sr(a) se sente tenso(a) quando *NOME DO IDOSO(A)* está por perto?**	**0**	**1**	**2**	**3**	**4**
**N. 10 - O(a) Sr(a) sente que sua saúde foi afetada por causa do seu envolvimento com *NOME DO IDOSO(A)*?**	**0**	**1**	**2**	**3**	**4**
**N. 11 - O(a) Sr(a) sente que Não tem tanta privacidade como gostaria por causa de *NOME DO IDOSO(A)*?**	**0**	**1**	**2**	**3**	**4**
**N. 12 - O(a) Sr(a) sente que sua vida social tem sido prejudicada em razão de ter de cuidar de *NOME DO IDOSO(A)*?**	**0**	**1**	**2**	**3**	**4**
**N. 13 - O(a) Sr(a) não se sente à vontade em receber visitas em casa por causa de *NOME DO IDOSO(A)*?**	**0**	**1**	**2**	**3**	**4**
**N. 14 - O(a) Sr(a) sente que *NOME DO IDOSO(A)* espera que o(a) Sr(a) cuide dele(a) como se fosse a única pessoa de quem ele(a) pode depender?**	**0**	**1**	**2**	**3**	**4**
**N. 15 - O(a) Sr(a) sente que não tem dinheiro suficiente para cuidar de *NOME DO IDOSO(A)* somando-se às suas outras despesas?**	**0**	**1**	**2**	**3**	**4**
**N. 16 - O(a) Sr(a) sente que será incapaz de cuidar de *NOME DO IDOSO(A)* por muito mais tempo?**	**0**	**1**	**2**	**3**	**4**
**N. 17 - O(a) Sr(a) sente que perdeu o controle de sua vida desde a doença de *NOME DO IDOSO(A)*?**	**0**	**1**	**2**	**3**	**4**
**N. 18 - O(a) Sr(a) gostaria de simplesmente deixar que outra pessoa cuidasse de *NOME DO IDOSO(A)*?**	**0**	**1**	**2**	**3**	**4**
**N. 19 - O(a) Sr(a) se sente em dúvida sobre o que fazer por *NOME DO IDOSO(A)*?**	**0**	**1**	**2**	**3**	**4**
**N. 20 - O(a) Sr(a) sente que deveria estar fazendo mais por *NOME DO IDOSO(A)*?**	**0**	**1**	**2**	**3**	**4**
**N. 21 - O(a) Sr(a) sente que poderia cuidar melhor de *NOME DO IDOSO(A)*?**	**0**	**1**	**2**	**3**	**4**
**N. 22 - De uma maneira geral, quanto o(a) Sr(a) se sente sobrecarregado por cuidar de *NOME DO IDOSO(A)*?**	**0**	**1**	**2**	**3**	**4**

SEÇÃO K. ANTROPOMETRIA

HORA DE INÍCIO: |__|__| : |__|__|

K.14

O(a) Sr.(a) fez alguma cirurgia no braço ou na mão que usa regularmente, nos últimos três meses?

SIM	1 →	**VÁ PARA A K1a**
NÃO	2	
NS	8	
NR	9	

K.15A

Agora vou usar um instrumento que se chama **DINAMÔMETRO** para testar a força da sua mão. Este teste somente pode ser feito se o Sr. NÃO sofreu nenhuma cirurgia no braço ou na mão, nos últimos três meses.

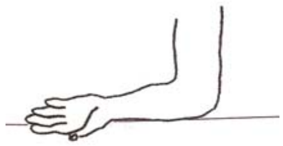

Use o braço que acha que tem mais força. Coloque o cotovelo sobre a mesa e estique o braço com a palma da mão para cima. Pegue as duas peças de metal juntas assim (faça a demonstração). Preciso ajustar o aparelho para o seu tamanho?

Agora, aperte bem forte. Tão forte quanto puder. As duas peças de metal não vão se mover, mas eu poderei ver qual a intensidade da força que o Sr. está usando. Vou fazer este teste 2 vezes. Avise-me se sentir alguma dor ou incômodo.

DINAMÔMETRO [ ]

ANOTE A MÃO USADA NO TESTE:

1.DIREITA **2.ESQUERDA** → [ ]

PRIMEIRA VEZ:

TENTOU MAS NÃO CONSEGUIU **95**

NÃO TENTOU, POR ACHAR ARRISCADO **96**

ENTREVISTADO INCAPACITADO **97**

RECUSOU-SE A TENTAR **98**

COMPLETOU O TESTE:

|____|____| ,|____|kg

SEGUNDA VEZ:

TENTOU MAS NÃO CONSEGUIU **95**

NÃO TENTOU, POR ACHAR ARRISCADO **96**

ENTREVISTADO INCAPACITADO **97**

RECUSOU-SE A TENTAR **98**

COMPLETOU O TESTE:

|____|____|,|____| kg

SEÇÃO K. ANTROPOMETRIA

ANOTE O APROPRIADO PARA A EXTREMIDADE CORRESPONDENTE	SIM	NÃO
**PARA O BRAÇO ESQUERDO**		
K.1a **TEM TODOS OS DEDOS?**	1 VÁ PARA K.2a	2
K.1b **TEM A MÃO?**	1 VÁ PARA K.1e	2
K.1c **TEM O ANTEBRAÇO?**	1 VÁ PARA K.1e	2
K.1d **TEM O BRAÇO?**	1	2
K.1e **TEM ALGUM TIPO DE PRÓTESE?**	1	2
**PARA O BRAÇO DIREITO**		
K.2a **TEM TODOS OS DEDOS?**	1 VÁ PARA K.3a	2
K.2b **TEM A MÃO?**	1 VÁ PARA K.2e	2
K.2c **TEM O ANTEBRAÇO?**	1 VÁ PARA K.2e	2
K.2d **TEM O BRAÇO?**	1	2
K.2e **TEM ALGUM TIPO DE PRÓTESE?**	1	2
**PARA A PERNA ESQUERDA**		
K.3a **TEM TODOS OS DEDOS?**	1 VÁ PARA K.4a	2
K.3b **TEM O PÉ?**	1 VÁ PARA K.3e	2
K.3c **TEM A PERNA?**	1 VÁ PARA K.3e	2
K.3d **TEM A COXA?**	1	2
K.3e **TEM ALGUM TIPO DE PRÓTESE?**	1	2
**PARA A PERNA DIREITA**		
K.4a **TEM TODOS OS DEDOS?**	1 VÁ PARA K.5	2
K.4b **TEM O PÉ?**	1 VÁ PARA K.4e	2
K.4c **TEM A PERNA?**	1 VÁ PARA K.4e	2
K.4d **TEM A COXA?**	1	2
K.4e **TEM ALGUM TIPO DE PRÓTESE?**	1	2

SEÇÃO K. ANTROPOMETRIA

TOMAR TRÊS MEDIDAS PARA TODAS AS PERGUNTAS ABAIXO

K.5 **Precisamos medir sua altura e para isso, queremos que o(a) Sr(a) fique descalço(a). Coloque-se de pé, com pés e calcanhares juntos e com suas costas e cabeça encostadas na parede. Olhe bem para a frente.**	ALTURA .... |___|___|___| **cm**<br>NÃO CONSEGUE PARAR DE PÉ **999**
K.6 **MEDIDA DA ALTURA DOS JOELHOS**	ALTURA .... |___|___| **cm**
K.7 **CIRCUNFERÊNCIA DO BRAÇO**	CIRCUNFERÊNCIA DO BRAÇO .... |___|___| **cm**
K.8 **CIRCUNFERÊNCIA DA CINTURA**	CINTURA .... |___|___|___| **cm**<br>NÃO CONSEGUE PARAR DE PÉ **999**
K.9 **CIRCUNFERÊNCIA DO QUADRIL**	QUADRIL .... |___|___|___| **cm**<br>NÃO CONSEGUE PARAR DE PÉ **999**
K.10 **DOBRA CUTÂNEA TRICIPITAL**	PREGA TRICIPITAL .... |___|___| **mm**
K.10a **DOBRA CUTÂNEA SUBESCAPULAR**	PREGA SUBESCAPULAR .... |___|___| **mm**
K.11 **PESO**	PESO .... |__|__|__|__| . |__| **kg**<br>NÃO CONSEGUE PARAR DE PÉ **999**
K.12 **CIRCUNFERÊNCIA DE PANTURRILHA**	CIRCUNFERÊNCIA DE PANTURRILHA .... |___|___| **cm**
K.12a **LARGURA DO COTOVELO**	LARGURA DO COTOVELO .... |___|___| **cm**
K.13 **CIRCUNFERÊNCIA DO PUNHO**	CIRCUNFERÊNCIA DO PULSO .... |___|___| **cm**

OBS.: Se ele(a) não fez o teste do dinamômetro a 1ª vez, passe para a Seção L.

K.15

Agora vamos repetir o teste com o dinamômetro, mas com o braço em outra posição.

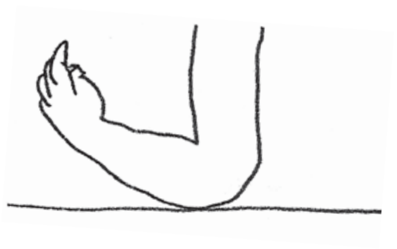

Use o braço que acha que tem mais força. Coloque o cotovelo sobre a mesa e estique o braço com a palma da mão para cima. Pegue as duas peças de metal juntas assim (faça a demonstração). Preciso ajustar o aparelho para o seu tamanho?

Agora, aperte bem forte. Tão forte quanto puder. As duas peças de metal não vão se mover, mas eu poderei ver qual a intensidade da força que o Sr. está usando. Vou fazer este teste 2 vezes. Avise-me se sentir alguma dor ou incômodo.

DINAMÔMETRO |____|

ANOTE A MÃO USADA NO TESTE:

1.DIREITA **2.ESQUERDA** → |____|

PRIMEIRA VEZ:

TENTOU MAS NÃO CONSEGUIU **95**

NÃO TENTOU, POR ACHAR ARRISCADO **96**

ENTREVISTADO INCAPACITADO **97**

RECUSOU-SE A TENTAR **98**

COMPLETOU O TESTE:

|____|____| ,|____|kg

SEGUNDA VEZ:

TENTOU MAS NÃO CONSEGUIU **95**

NÃO TENTOU, POR ACHAR ARRISCADO **96**

ENTREVISTADO INCAPACITADO **97**

RECUSOU-SE A TENTAR **98**

COMPLETOU O TESTE:

|____|____|,|____| kg

SEÇÃO L. MOBILIDADE E FLEXIBILIDADE

PARA CONTINUARMOS PRECISO REALIZAR ALGUNS TESTES PARA MEDIR SUA MOBILIDADE E FLEXIBILIDADE. PRIMEIRO VOU-LHE MOSTRAR COMO FAZER CADA MOVIMENTO E, EM SEGUIDA, GOSTARIA QUE O(A) SR(A) TENTASSE REPETIR OS MEUS MOVIMENTOS. SE ACHAR QUE NÃO TEM CONDIÇÕES DE FAZÊ-LO, ACHAR ARRISCADO OU SE SENTIR INSEGURO(A) DIGA-ME E PASSAREMOS PARA OUTRO TESTE.

L.1 FILTRO

INCAPACITADO PARA REALIZAR QUALQUER TESTE DE FLEXIBILIDADE E MOBILIDADE.

SIM .... **1** → ENCERRE A ENTREVISTA

NÃO **2**

Timed Up & Go Test (TUGT)

L.10

ATENÇÃO:

Se **NÃO FOR POSSÍVEL LEVAR** a cadeira padrão, **USAR UMA CADEIRA COM BRAÇO** para o teste e medir a altura do assento e braço da cadeira conforme o desenho. Não havendo cadeira com braços não faça o teste e vá para L.1a.

Usou a cadeira padrão ( ) Sim ( ) Não

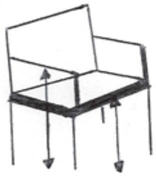

Altura do Braço:_________

Altura do Assento:_______

Nós vamos fazer um teste que consiste em se levantar da cadeira, caminhar o percurso delimitado, virar no final do percurso, retornar para a cadeira e sentar-se novamente (demonstre o teste). O(A) sr(a) pode utilizar qualquer dispositivo que esteja acostumado (bengala, andador ou muleta).
Gostaria que o(a) sr(a) se sentasse, apoiasse suas costas no encosto da cadeira, apoiasse seus pés no chão e colocasse seus braços nos braços da cadeira (aguarde o(a) idoso(a) se posicionar e auxilie-o(a) se for necessário).
Quando eu disser "já" gostaria que se levantasse e caminhasse por esse trajeto delimitado em sua velocidade habitual de caminhada, ou seja, da mesma forma que o(a) sr(a) caminharia na rua. No final do trajeto, gostaria que o(a) sr(a) virasse e retornasse para a cadeira e sentasse novamente. Podemos começar?

"**JÁ**" (acione o cronômetro)

OBSERVAÇÕES: Após **O COMANDO JÁ** o cronômetro deve ser disparado. Mesmo que o idoso demore para se levantar da cadeira, esse tempo deverá ser contado no teste. O cronômetro deve ser parado somente quando o idoso retornar e sentar-se novamente na cadeira. Se o idoso parar ao levantar, ao girar ou antes de sentar-se o teste deverá ser repetido.

TEMPO GASTO NA TAREFA

REALIZOU O TESTE EM: |_____|_____| , |_____|_____| segundos **1**

NÃO REALIZOU O TESTE **2**

L.1a **PÉS LADO A LADO**

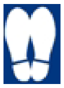

Quero que o Sr. fique em pé, com os pés juntos, um ao lado do outro, mantendo os olhos abertos. Por favor, mantenha essa posição até eu avisar (dez segundos).

Pode usar os braços, dobrar os joelhos ou mexer com o corpo, para se equilibrar; porém, tente não mexer os pés.

MANTEVE A POSIÇÃO POR 10 SEGUNDOS .... **1 (1 PONTO)** → **VÁ PARA L.1c**

NÃO MANTEVE A POSIÇÃO POR 10 SEGUNDOS .... **2 (O PONTOS)**

NÃO TENTOU .... **98 (O PONTOS)**

REALIZOU O TESTE EM: segundos |_____|_____|

ESCORE: |_____|pontos

SEÇÃO L. MOBILIDADE E FLEXIBILIDADE

L. 1b

se o(a) idoso(a) **não tentou ou falhou** assinale o por que e depois vá para a questão L.3a

TENTOU MAS NÃO CONSEGUIU	95	**VÁ PARA L..3a**
NÃO PODE MANTER A POSIÇÃO SEM AUXÍLIO	90	
NÃO TENTOU, ENTREVISTADORA SENTIU-SE INSEGURA .	91	
NÃO TENTOU, IDOSO(A) SENTIU-SE INSEGURO(A)	96	
IDOSO(A) INCAPAZ DE COMPREENDER AS INSTRUÇÕES .	92	
OUTRO .... *ESPECIFIQUE*	93	

L.1c UM PÉ UM POUCO À FRENTE DO OUTRO

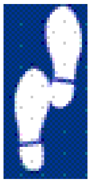

Agora, quero que o(a) Sr(a) tente ficar em pé, com o calcanhar de um dos pés encostado na lateral do dedão do pé oposto, por uns dez segundos. O Sr. pode usar qualquer pé, aquele que lhe dê mais segurança. Pode usar os braços, dobrar os joelhos ou mexer o corpo para se equilibrar; porém, tente não mexer os pés. Por favor, mantenha essa posição até eu avisar (dez segundos).

MANTEVE A POSIÇÃO POR 10 SEGUNDOS .... **1 (1 PONTO)** → **VÁ PARA L.2**

NÃO MANTEVE A POSIÇÃO POR 10 SEGUNDOS .... **2 (O PONTOS)**

NÃO TENTOU .... **98 (O PONTOS)**

REALIZOU O TESTE EM: segundos |_____|_____|

ESCORE: |_____|pontos

L. 1d

Se o(a) idoso(a) **não tentou ou falhou** assinale o por que e depois vá para a questão L.3a:

TENTOU MAS NÃO CONSEGUIU	95	**VÁ PARA L..3a**
NÃO PODE MANTER A POSIÇÃO SEM AUXÍLIO	90	
NÃO TENTOU, ENTREVISTADORA SENTIU-SE INSEGURA .	91	
NÃO TENTOU, IDOSO(A) SENTIU-SE INSEGURO(A)	96	
IDOSO(A) INCAPAZ DE COMPREENDER AS INSTRUÇÕES .	92	
OUTRO .... *ESPECIFIQUE*	93	

L.2 UM PÉ ATRÁS DO OUTRO

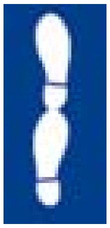

Agora, quero que o(a) Sr(a) tente ficar em pé, com o calcanhar de um dos pés na frente do outro pé, por uns dez segundos. O(A) Sr(a) pode usar qualquer pé, aquele que lhe dê mais segurança.

Pode usar os braços, dobrar os joelhos ou mexer o corpo para se equilibrar; porém, tente não mexer os pés. Por favor, mantenha essa posição até eu avisar (dez segundos).

MANTEVE A POSIÇÃO POR 10 SEGUNDOS .... **1 (2 PONTOS)** → VÁ PARA L.3

MANTEVE A POSIÇÃO DE 3 A 9.99 SEGUNDOS .... **2 (1 PONTO)** → VÁ PARA L.3

MANTEVE A POSIÇÃO POR MENOS DE 3 SEGUNDOS ..... **3 (O PONTOS)**

NÃO TENTOU .... **98 (O PONTOS)**

REALIZOU O TESTE EM: segundos |_____|_____|

ESCORE: |_____|pontos

L. 2a

Se o(a) idoso(a) **NÃO TENTOU OU FALHOU** assinale o por que e depois vá para a questão L.3a:

TENTOU MAS NÃO CONSEGUIU	95	**VÁ PARA L.3a**
NÃO PODE MANTER A POSIÇÃO SEM AUXÍLIO	90	
NÃO TENTOU, ENTREVISTADORA SENTIU-SE INSEGURA	91	
NÃO TENTOU, IDOSO(A) SENTIU-SE INSEGURO(A)	96	
IDOSO(A) INCAPAZ DE COMPREENDER AS INSTRUÇÕES	92	
OUTRO ______________________________ *ESPECIFIQUE*	93	

SEÇÃO L. MOBILIDADE E FLEXIBILIDADE

L.3

Ficando de pé, gostaria que o(a) Sr(a) tentasse se equilibrar em um pé só, sem se apoiar em nada. Tente primeiro com qualquer um dos pés, depois tentaremos com o outro.

Eu contarei o tempo e vou lhe dizer quando começar e terminar (dez segundos). Podemos parar a qualquer momento que o(a) Sr(a) sinta que está perdendo o equilíbrio.

PÉ DIREITO

TENTOU MAS NÃO CONSEGUIU 95
NÃO TENTOU, POR ACHAR ARRISCADO 96
RECUSOU-SE A TENTAR 98

REALIZOU O TESTE EM: SEGUNDOS |____|____|

PÉ ESQUERDO

TENTOU MAS NÃO CONSEGUIU 95
NÃO TENTOU, POR ACHAR ARRISCADO 96
RECUSOU-SE A TENTAR 98

REALIZOU O TESTE EM: SEGUNDOS |____|____|

L.3a

ESCORE DE EQUILÍBRIO

TOTAL DE PONTOS |____|____|

(Some os pontos das questões L1a, L1c e L2)

L.3c CAMINHADA NÚMERO 1

DELIMITE O TRAJETO

Esse é o trajeto de caminhada. Eu gostaria que o(a) Sr(a) andasse de um ponto a outro desse percurso na sua velocidade habitual de caminhada ou seja, da mesma forma que o(a) Sr(a) caminharia numa rua para ir a uma loja.

REALIZOU O TESTE EM: |_____|_____| , |_____|_____| segundos 1
NÃO REALIZOU O TESTE .... 2 → **VÁ PARA L.3e**

L.3d CAMINHADA NÚMERO 1

Para realizar a primeira caminhada o(a) idoso precisou utilizar algum dispositivo de ajuda?

SIM, BENGALA 1
SIM, OUTRO 2
ESPECIFIQUE
NÃO 3

L. 3e

Se o(a) idoso(a) **não tentou ou falhou** assinale o por que e depois vá para a questão L.3i:

TENTOU MAS NÃO CONSEGUIU 90
NÃO PODE ANDAR SEM AUXÍLIO 91
NÃO TENTOU, ENTREVISTADORA SENTIU-SE INSEGURA . 92
NÃO TENTOU, IDOSO(A) SENTIU-SE INSEGURO(A) 93
IDOSO(A) INCAPAZ DE COMPREENDER AS INSTRUÇÕES . 94
OUTRO 95
ESPECIFIQUE
RECUSOU-SE 96

(90–96) → **VÁ PARA L.3i**

L.3f CAMINHADA NÚMERO 2

Agora, eu gostaria que o(a) Sr(a) repetisse a mesma caminhada. Lembre-se que é preciso andar de um ponto a outro do percurso na sua velocidade habitual de caminhada.

REALIZOU O TESTE EM: |_____|_____| , |_____|_____| segundos .. 1
NÃO REALIZOU O TESTE .... 2 → **VÁ PARA L.3h**

L.3g CAMINHADA NÚMERO 2

Para realizar a segunda caminhada o(a) idoso precisou utilizar algum dispositivo de ajuda?

SIM, BENGALA 1
SIM, OUTRO 2
ESPECIFIQUE
NÃO 3

SEÇÃO L. MOBILIDADE E FLEXIBILIDADE

L. 3h

Se o(a) idoso(a) **não tentou ou falhou** assinale o por que e depois vá para a questão L.3i:

TENTOU MAS NÃO CONSEGUIU	90	VÁ PARA L. 3i
NÃO PODE ANDAR SEM AUXÍLIO	91	
NÃO TENTOU, ENTREVISTADORA SENTIU-SE INSEGURA	92	
NÃO TENTOU, IDOSO(A) SENTIU-SE INSEGURO(A)	93	
IDOSO(A) INCAPAZ DE COMPREENDER AS INSTRUÇÕES	94	
OUTRO ______ *ESPECIFIQUE*	95	
RECUSOU-SE	96	

L. 3i

TEMPO DE CAMINHADA E ESCORE

REGISTRE O MENOR TEMPO OBTIDO ENTRE AS DUAS CAMINHADAS. SE FEZ SÓ UMA, ANOTE ESSE TEMPO DE PERCURSO:

|_____|_____| .|_____|_____| segundos

Escore = |_____|_____| pontos

PARA 3 METROS

Não foi capaz de fazer o teste	1	**(0 pontos)**
Tempo maior que 6,52 segundos	2	**(1 pontos)**
Tempo entre 4,66 e 6,52 segundos	3	**(2 pontos)**
Tempo entre 3,62 e 4,65 segundos	4	**(3 pontos)**
Tempo menor que 3,62 segundos	5	**(4 pontos)**

O PROXIMO TESTE VAI MEDIR A FORÇA DE SUAS PERNAS.

L.4

O(A) Sr(a) se sente seguro(a) para tentar se levantar, rapidamente, da cadeira, sem utilizar seus braços?

SIM	1	
NÃO	2	→ VÁ PARA L.5a

L.5

Agora, quero que o(a) Sr(a) tente levantar e sentar da cadeira, **UMA ÚNICA VEZ** sem utilizar seus braços como apoio.

REALIZOU O TESTE SEM UTILIZAR OS BRAÇOS	1	→ VÁ PARA L.6
REALIZOU O TESTE UTILIZANDO OS BRAÇOS	2	
NÃO TENTOU	98	

L. 5a

se o(a) idoso(a) **não tentou ou falhou** assinale o por que e depois vá para a questão L.7b:

TENTOU MAS NÃO CONSEGUIU	95	VÁ PARA L 7b
NÃO PODE REALIZAR O TESTE SEM AUXÍLIO	90	
NÃO TENTOU, ENTREVISTADORA SENTIU-SE INSEGURA	91	
NÃO TENTOU, IDOSO(A) SENTIU-SE INSEGURO(A)	96	
IDOSO(A) INCAPAZ DE COMPREENDER AS INSTRUÇÕES	92	
OUTRO ______ *ESPECIFIQUE*	93	

SEÇÃO L. MOBILIDADE E FLEXIBILIDADE

**L.6** O Sr. se sente confiante para tentar levantar-se da cadeira, com os braços cruzados cinco vezes seguidas?	SIM .... **1**<br>NÃO .... **2** → VÁ PARA L.7a
**L.7** Agora, mantendo os braços cruzados sobre o peito, quero que o(a) Sr(a) se levante da cadeira, o mais rapidamente possível, cinco vezes sem fazer nenhuma pausa. Cada vez que o Sr. conseguir ficar em pé, sente-se de novo e, levante-se novamente (60 segundos).	REALIZOU O TESTE SEM UTILIZAR OS BRAÇOS .... **1** → VÁ PARA L 7b<br>.. EM: |_____|_____| segundos<br>ANOTE A ALTURA DO ASSENTO DA CADEIRA |____|____|cm<br>REALIZOU O TESTE UTILIZANDO OS BRAÇOS .... **2 (0 PONTOS)**<br>NÃO TENTOU .... **98 (0 PONTOS)**
**L. 7a** Se o(a) idoso(a) **<u>não tentou ou falhou</u>** assinale o por que e depois vá para a questão L.7b:	TENTOU MAS NÃO CONSEGUIU .... **95**<br>NÃO PODE REALIZAR O TESTE SEM AUXÍLIO .... **90**<br>NÃO TENTOU, ENTREVISTADORA SENTIU-SE INSEGURA . **91**<br>NÃO TENTOU, IDOSO(A) SENTIU-SE INSEGURO(A) .... **96**<br>IDOSO(A) INCAPAZ DE COMPREENDER AS INSTRUÇÕES . **92**<br>OUTRO .... **93**<br>*ESPECIFIQUE*<br>} VÁ PARA L. 7b
**L. 7b** **ESCORE**	Não foi capaz de fazer o teste - não realizou os 5 movimentos ou utilizou um tempo > que 60 segundos para completar o teste .... **1 (0 pontos)**<br>Tempo utilizado no teste = 16,70 segundos ou mais .... **2 (1 pontos)**<br>Tempo utilizado no teste entre 13,70 e 16,69 segundos ...... **3 (2 pontos)**<br>Tempo utilizado no teste entre 11,20 e 13,69 segundos ...... **4 (3 pontos)**<br>Tempo utilizado no teste = 11,19 segundos ou menos .... **5 (4 pontos)**<br>**TOTAL DE PONTOS** |_____|_____|
**L. 7b** **ESCORE DA BATERIA DE TESTE DE DESEMPENHO FÍSICO**	ESCORE TOTAL DOS TESTES DE EQUILÍBRIO .... |_____|_____| ESCORE TOTAL DOS TESTES DE VELOCIDADE .... |_____|_____| ESCORE TOTAL DOS TESTES DE FORÇA DE MEMBROS INFERIORES |_____|_____| **ESCORE TOTAL** |_____|_____| (some os pontos acima)

O PROXIMO TESTE VAI MEDIR SUA FLEXIBILIDADE

L.8

Nas últimas seis semanas, o Sr. fez cirurgia de catarata ou qualquer outro procedimento em seus olhos?

SIM	1	→ ENCERRE A ENTREVISTA
NÃO	2	
NS	8	
NR	9	

L.9

Para este próximo teste, o(a) Sr(a). terá que se agachar e apanhar um lápis do chão.

O(A) Sr(a) só poderá realizar esse teste se NÃO sofreu cirurgia de catarata nas últimas seis semanas.

Começando, fique em pé , agache-se, apanhe este lápis, e fique novamente em pé.

COLOQUE O LÁPIS NO CHÃO, NA FRENTE DO ENTREVISTADO E AVISE-O QUANDO COMEÇAR. SE O ENTREVISTADO NÃO CONSEGUIR REALIZAR O TESTE EM MENOS DE 30 SEGUNDOS, NÃO O DEIXE CONTINUAR.

TENTOU MAS NÃO CONSEGUIU	**95**
NÃO PODE MANTER A POSIÇÃO SEM AUXÍLIO	**90**
NÃO TENTOU, ENTREVISTADORA SENTIU-SE INSEGURA	**91**
NÃO TENTOU, IDOSO(A) SENTIU-SE INSEGURO(A)	**96**
IDOSO(A) INCAPAZ DE COMPREENDER AS INSTRUÇÕES	**92**
OUTRO ______ *ESPECIFIQUE*	**93**
RECUSOU-SE A TENTAR	**98**

COMPLETOU O TESTE EM:

segundos |_____|_____|

HORA DE TÉRMINO: |__|__| : |__|__|
